# Supplementary material for: Novel CF₃-Substituted Pyridine- and Pyrimidine-Based Fluorescent Probes for Lipid Droplet Bioimaging
Source: Int J Mol Sci. 2025 May 30;26(11):5271. doi: 10.3390/ijms26115271 (PMC12154354; doi:10.3390/ijms26115271)
Supplement: Supplementary file 1 [file ijms-26-05271-s001.zip › ijms-3620137-supplementary.pdf]

Supplementary Material

Novel CF_3-Substituted Pyridine- and Pyrimidine-based Fluorescent Probes for Lipid Droplets Bioimaging

Dmitrii L. Chizhov[a], Yuriy A. Kvashnin[a], Nadezhda S. Demina[a], Ekaterina F. Zhilina[a], Artem S. Minin[b], Natalia A. Verbitskaia[b], Gennady L. Rusinov[a], Egor V. Verbitskiy[a,b,*], Valery N. Charushin[a,b]

[a]*I.Ya. Postovsky Institute of Organic Synthesis, Ural Branch of the Russian Academy of Sciences, S. Kovalevskaya Str., 22, Ekaterinburg, 620066, Russia*

[b]*Ural Federal University, Mira St. 19, Ekaterinburg, 620002, Russia*

Table of contents

General Information. 6

Figure S1. ^{1}H NMR (500 MHz, $CDCl_3$) spectrum of **3**. 7

Figure S2. ^{19}F NMR (471 MHz, $CDCl_3$) spectrum of **3**. 7

Figure S3. ^{13}C NMR (126 MHz, $CDCl_3$) spectrum of **3**. 8

Figure S4. ^{1}H NMR (500 MHz, DMSO-d_6) spectrum of **4**. 8

Figure S5. ^{19}F NMR (471 MHz, DMSO-d_6) spectrum of **4**. 9

Figure S6. ^{13}C NMR (126 MHz, DMSO-d_6) spectrum of **4**. 9

Figure S7. ^{1}H NMR (500 MHz, $CDCl_3$) spectrum of **5**. 10

Figure S8. ^{19}F NMR (471 MHz, $CDCl_3$) spectrum of **5**. 10

Figure S9. ^{13}C NMR (126 MHz, $CDCl_3$) spectrum of **5**. 11

Figure S10. ^{1}H NMR (500 MHz, DMSO-d_6) spectrum of **6**. 11

Figure S11. ^{19}F NMR (471 MHz, DMSO-d_6) spectrum of **6**. 12

Figure S12. ^{13}C NMR (126 MHz, DMSO-d_6) spectrum of **6**. 12

Figure S13. ^{1}H NMR (500 MHz, $CDCl_3$) spectrum of **7**. 13

Figure S14. ^{19}F NMR (471 MHz, $CDCl_3$) spectrum of **7**. 13

Figure S15. ^{13}C NMR (126 MHz, $CDCl_3$) spectrum of **7**. 14

Figure S16. ^{1}H NMR (600 MHz, DMSO-d_6) spectrum of **9a**. 14

Figure S17. ^{19}F NMR (376 MHz, DMSO-d_6) spectrum of **9a**. 15

Figure S18. ^{13}C NMR (151 MHz, DMSO-d_6) spectrum of **9a**. 15

Figure S19. ^{1}H NMR (600 MHz, DMSO-d_6) spectrum of **9b**. 16

Figure S20. ^{19}F NMR (376 MHz, $CDCl_3$) spectrum of **9b**. 16

Figure S21. ^{13}C NMR (151 MHz, DMSO-d_6) spectrum of **9b**. 17

Figure S22. ^{1}H NMR (600 MHz, DMSO-d_6) spectrum of **9c**. 17
Figure S23. ^{19}F NMR (471 MHz, DMSO-d_6) spectrum of **9c**. 18
Figure S24. ^{13}C NMR (151 MHz, DMSO-d_6) spectrum of **9c**. 18
Figure S25. ^{1}H NMR (600 MHz, DMSO-d_6) spectrum of **9d**. 19
Figure S26. ^{19}F NMR (376 MHz, DMSO-d_6) spectrum of **9d**. 19
Figure S27. ^{13}C NMR (151 MHz, DMSO-d_6) spectrum of **9d**. 20
Figure S28. ^{1}H NMR (600 MHz, DMSO-d_6) spectrum of **9e**. 20
Figure S29. ^{19}F NMR (376 MHz, DMSO-d_6) spectrum of **9e**. 21
Figure S30. ^{13}C NMR (151 MHz, DMSO-d_6) spectrum of **9e**. 21
Figure S31. ^{1}H NMR (500 MHz, $CDCl_3$) spectrum of **10a**. 22
Figure S32. ^{19}F NMR (471 MHz, DMSO-d_6) spectrum of **10a**. 22
Figure S33. ^{13}C NMR (126 MHz, $CDCl_3$) spectrum of **10a**. 23
Figure S34. ^{1}H NMR (500 MHz, $CDCl_3$) spectrum of **10b**. 23
Figure S35. ^{19}F NMR (471 MHz, $CDCl_3$) spectrum of **10b**. 24
Figure S36. ^{13}C NMR (126 MHz, $CDCl_3$) spectrum of **10b**. 24
Figure S37. ^{1}H NMR (400 MHz, $CDCl_3$) spectrum of **10c**. 25
Figure S38. ^{19}F NMR (475 MHz, $CDCl_3$) spectrum of **10c**. 25
Figure S39. ^{13}C NMR (126 MHz, $CDCl_3$) spectrum of **10c**. 26
Figure S40. ^{1}H NMR (400 MHz, $CDCl_3$) spectrum of **10d**. 26
Figure S41. ^{19}F NMR (376 MHz, $CDCl_3$) spectrum of **10d**. 27
Figure S42. ^{13}C NMR (126 MHz, $CDCl_3$) spectrum of **10d**. 27
Figure S43. ^{1}H NMR (400 MHz, $CDCl_3$) spectrum of **10e**. 28
Figure S44. ^{19}F NMR (376 MHz, $CDCl_3$) spectrum of **10e**. 28
Figure S45. ^{13}C NMR (126 MHz, $CDCl_3$) spectrum of **10e**. 29
Figure S46. Absorption spectrum (--), excitation (–) and emission (–) spectra of **9a** in MeCN. 29
Figure S47. Absorption spectrum (--), excitation (–) and emission (–) spectra of **9b** in MeCN. 29
Figure S48. Absorption spectrum (--), excitation (–) and emission (–) spectra of **9c** in MeCN. 29
Figure S49. Absorption spectrum (--), excitation (–) and emission (–) spectra of **9d** in MeCN. 29
Figure S50. Absorption spectrum (--), excitation (–) and emission (–) spectra of **9e** in MeCN. 30
Figure S51. Absorption spectrum (--), excitation (–) and emission (–) spectra of **10a** in MeCN. 30
Figure S52. Absorption spectrum (--), excitation (–) and emission (–) spectra of **10b** in MeCN. 30
Figure S53. Absorption spectrum (--), excitation (–) and emission (–) spectra of **10c** in MeCN. 30
Figure S54. Absorption spectrum (--), excitation (–) and emission (–) spectra of **10d** in MeCN. 30
Figure S55. Absorption spectrum (--), excitation (–) and emission (–) spectra of **10e** in MeCN. 30
Figure S56. Emission spectra of **9a-e** in solid state. 30

Table S1. Detailed data of the fluorescence lifetime measurements, k_r – radiative transition rate constant ($k_r = \Phi_F/\tau_{avg}$) and k_{nr} – nonradiative transition rate constant ($k_{nr} = 1/\tau_{avg} - k_r$) values of **9a-e** and **10a-e** in MeCN: τ – lifetime, *f* - fractional contribution, τ_{avg} – average lifetime, χ^2 - chi-squared distribution.........30
Figure S57. Time-resolved fluorescence lifetime decay profile of solution **9a** in MeCN (green), instrumental response function (IRF, blue). λ_{ex} = 300 nm, λ_{em} = 551 nm. 31
Figure S58. Time-resolved fluorescence lifetime decay profile of solution **9b** in MeCN (green), instrumental response function (IRF, blue). λ_{ex} = 300 nm, λ_{em} = 556 nm. 31
Figure S59. Time-resolved fluorescence lifetime decay profile of solution **9c** in MeCN (green), instrumental response function (IRF, blue). λ_{ex} = 300 nm, λ_{em} = 550 nm. 31
Figure S60. Time-resolved fluorescence lifetime decay profile of solution **9d** in MeCN (green), instrumental response function (IRF, blue). λ_{ex} = 300 nm, λ_{em} = 546 nm. 31
Figure S61. Time-resolved fluorescence lifetime decay profile of solution **9e** in MeCN (green), instrumental response function (IRF, blue). λ_{ex} = 300 nm, λ_{em} = 564 nm. 31
Figure S62. Time-resolved fluorescence lifetime decay profile of solution **10a** in MeCN (green), instrumental response function (IRF, blue). λ_{ex} = 375 nm, λ_{em} = 506 nm. 31
Figure S63. Time-resolved fluorescence lifetime decay profile of solution **10b** in MeCN (green), instrumental response function (IRF, blue). λ_{ex} = 375 nm, λ_{em} = 533 nm. 32
Figure S64. Time-resolved fluorescence lifetime decay profile of solution **10c** in MeCN (green), instrumental response function (IRF, blue). λ_{ex} = 375 nm, λ_{em} = 495 nm. 32
Figure S65. Time-resolved fluorescence lifetime decay profile of solution **10d** in MeCN (green), instrumental response function (IRF, blue). λ_{ex} = 375 nm, λ_{em} = 513 nm. 32
Figure S66. Time-resolved fluorescence lifetime decay profile of solution **10e** in MeCN (green), instrumental response function (IRF, blue). λ_{ex} = 375 nm, λ_{em} = 620 nm. 32
Table S2. Detailed data of the fluorescence lifetime measurements of solid **9a-e** and **10a-e**: τ – lifetime, *f* - fractional contribution, τ_{avg} – average lifetime, χ^2 - chi-squared distribution. 32
Figure S67. Time-resolved fluorescence lifetime decay profile of solid **9a** (green), instrumental response function (IRF, blue). λ_{ex} = 375 nm, λ_{em} = 518 nm. 33
Figure S68. Time-resolved fluorescence lifetime decay profile of solid **9b** (green), instrumental response function (IRF, blue). λ_{ex} = 375 nm, λ_{em} = 536 nm. 33
Figure S69. Time-resolved fluorescence lifetime decay profile of solid **9c** (green), instrumental response function (IRF, blue). λ_{ex} = 375 nm, λ_{em} = 515 nm. 33
Figure S70. Time-resolved fluorescence lifetime decay profile of solid **9d** (green), instrumental response function (IRF, blue). λ_{ex} = 375 nm, λ_{em} = 535 nm. 33
Figure S71. Time-resolved fluorescence lifetime decay profile of solid **9e** (green), instrumental response function (IRF, blue). λ_{ex} = 375 nm, λ_{em} = 525 nm. 33

Figure S72. Time-resolved fluorescence lifetime decay profile of solid **10a** (green), instrumental response function (IRF, blue). λ_{ex} = 375 nm, λ_{em} = 463 nm. 33
Figure S73. Time-resolved fluorescence lifetime decay profile of solid **10b** (green), instrumental response function (IRF, blue). λ_{ex} = 375 nm, λ_{em} = 470 nm. 33
Figure S74. Time-resolved fluorescence lifetime decay profile of solid **10c** (green), instrumental response function (IRF, blue). λ_{ex} = 375 nm, λ_{em} = 436 nm. 33
Figure S75. Time-resolved fluorescence lifetime decay profile of solid **10d** (green), instrumental response function (IRF, blue). λ_{ex} = 375 nm, λ_{em} = 456 nm. 34
Figure S76. Time-resolved fluorescence lifetime decay profile of solid **10e** (green), instrumental response function (IRF, blue). λ_{ex} = 375 nm, λ_{em} = 470 nm. 34
Figure S77. The fluorescence spectra of 10 μM **9a** in MeCN/H_2O mixtures with different water fractions (f_w). 35
Figure S78. A plot of I/I_0 versus the composition of the MeCN/H_2O mixture for **9a** at λ = 530 nm. 35
Figure S79. The fluorescence spectra of 10 μM **9b** in MeCN/H_2O mixtures with different water fractions (f_w). 35
Figure S80. A plot of I/I_0 versus the composition of the MeCN/H_2O mixture for **9b** at λ = 527 nm. 35
Figure S81. The fluorescence spectra of 10 μM **9c** in MeCN/H_2O mixtures with different water fractions (f_w). 35
Figure S82. A plot of I/I_0 versus the composition of the MeCN/H_2O mixture for **9c** at λ = 524 nm. 35
Figure S83. The fluorescence spectra of 10 μM **9d** in MeCN/H_2O mixtures with different water fractions (f_w). 35
Figure S84. A plot of I/I_0 versus the composition of the MeCN/H_2O mixture for **9d** at λ = 538 nm. 35
Figure S85. The fluorescence spectra of 10 μM **10a** in MeCN/H_2O mixtures with different water fractions (f_w). 36
Figure S86. A plot of I/I_0 versus the composition of the MeCN/H_2O mixture for **10a** at λ = 472 nm. 36
Figure S87. The fluorescence spectra of 10 μM **10b** in MeCN/H_2O mixtures with different water fractions (f_w). 36
Figure S88. A plot of I/I_0 versus the composition of the MeCN/H_2O mixture for **10b** at λ = 475 nm. 36
Figure S89. The fluorescence spectra of 10 μM **10c** in MeCN/H_2O mixtures with different water fractions (f_w). 36
Figure S90. A plot of I/I_0 versus the composition of the MeCN/H_2O mixture for **10c** at λ = 475 nm. 36
Figure S91. The fluorescence spectra of 10 μM **10d** in MeCN/H_2O mixtures with different water fractions (f_w). 36
Figure S92. A plot of I/I_0 versus the composition of the MeCN/H_2O mixture for **10d** at λ = 475 nm. 36
Biological investigation 37

Cell cultivation 37
The staining protocol 37
Cell cytotoxicity and phototoxicity...38
Figure S93. HRMS spectrum of **9a**...39
Figure S94. HRMS spectrum of **9b**. 39
Figure S95. HRMS spectrum of **9c**..40
Figure S96. HRMS spectrum of **9d**. 40
Figure S97. HRMS spectrum of **9e**..41

General Information.

All reagents were purchased from commercial sources and were used without further purification. 1,4-Dioxane for the microwave-assisted Suzuki cross-coupling reaction were deoxygenated by bubbling argon for 1 h.

The ^{1}H and ^{13}C NMR spectra were recorded on a Bruker DRX-400, AVANCE-500 and AVANCE-600 instruments using Me_4Si as an internal standard. Elemental analysis was carried on a Eurovector EA 3000 automated analyzer. High resolution mass spectrometry was performed using a Bruker maXis Impact HD spectrometer. Melting points were determined on Boetius combined heating stages and were not corrected.

Flash-column chromatography was carried out using Alfa Aesar silica gel 0.040-0.063 mm (230-400 mesh), eluting with chloroform. The progress of reactions and the purity of compounds were checked by TLC on Sorbfil plates (Russia), in which the spots were visualized with UV light (λ 254 or 365 nm).

UV/vis spectra were recorded with Shimadzu UV-2600 spectrophotometer. Photoluminescent spectra were recorded on a FS5 Edinburgh Instruments spectrofluorometer. UV/vis and fluorescence spectra of solutions were recorded using standard 1 cm quartz cells at room temperature. Fluorescence spectra of solid powders were recorded using SC-10 module at room temperature. Absolute quantum yields have been measured using integrating sphere SC-30.

The emission lifetimes have been measured using TCSPC option of FS5 Edinburgh Instruments spectrofluorometer. The sample has been excited by EPL-375 picosecond pulsed diode centered at 375 nm. The instrument response function (IRF) has been recorded under described conditions by replacing the sample with a silica diffuser. The time decay data have been analyzed by nonlinear least-squares fitting with deconvolution of the IRF using the Fluoracle software package.

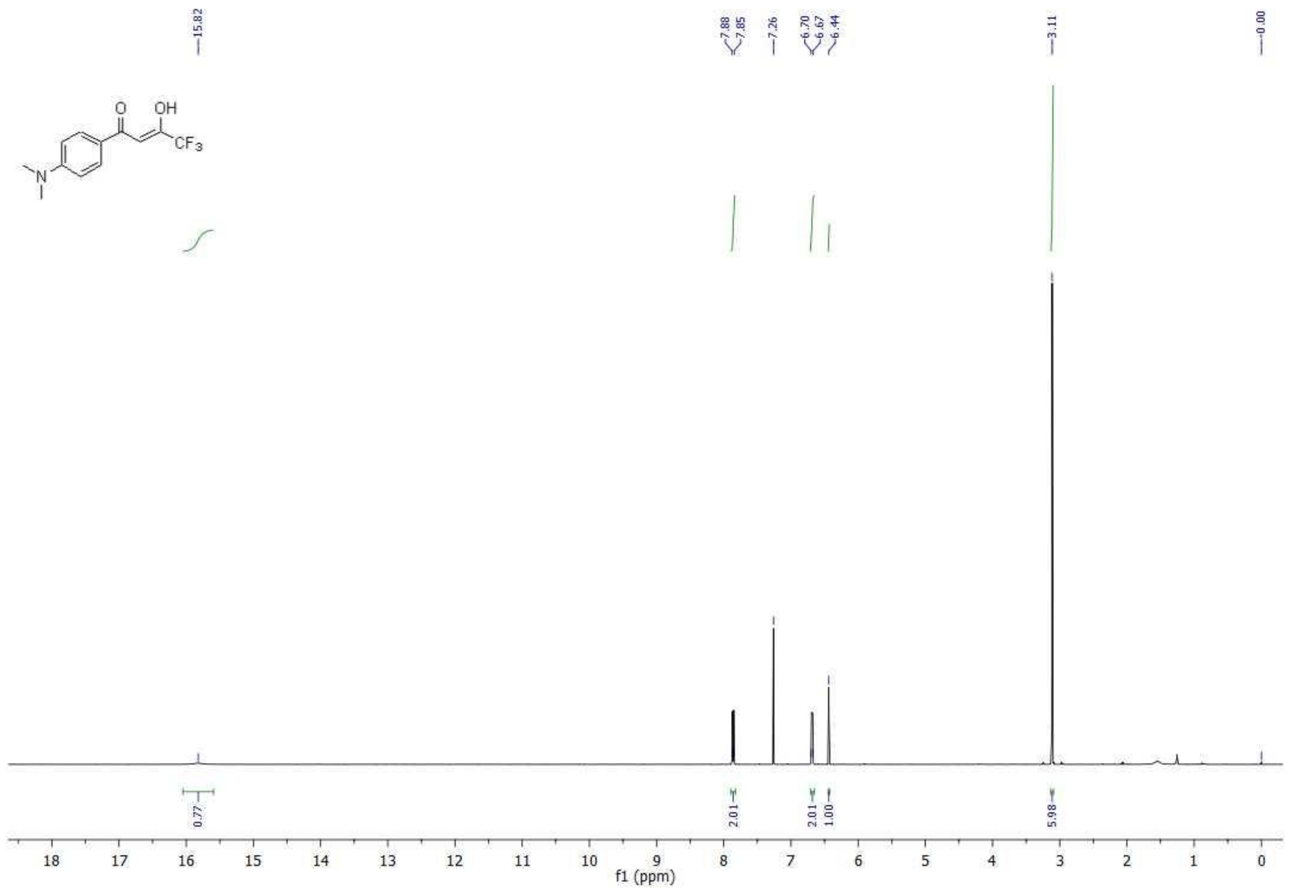

Figure S1. ^{1}H NMR (500 MHz, $CDCl_3$) spectrum of **3**.

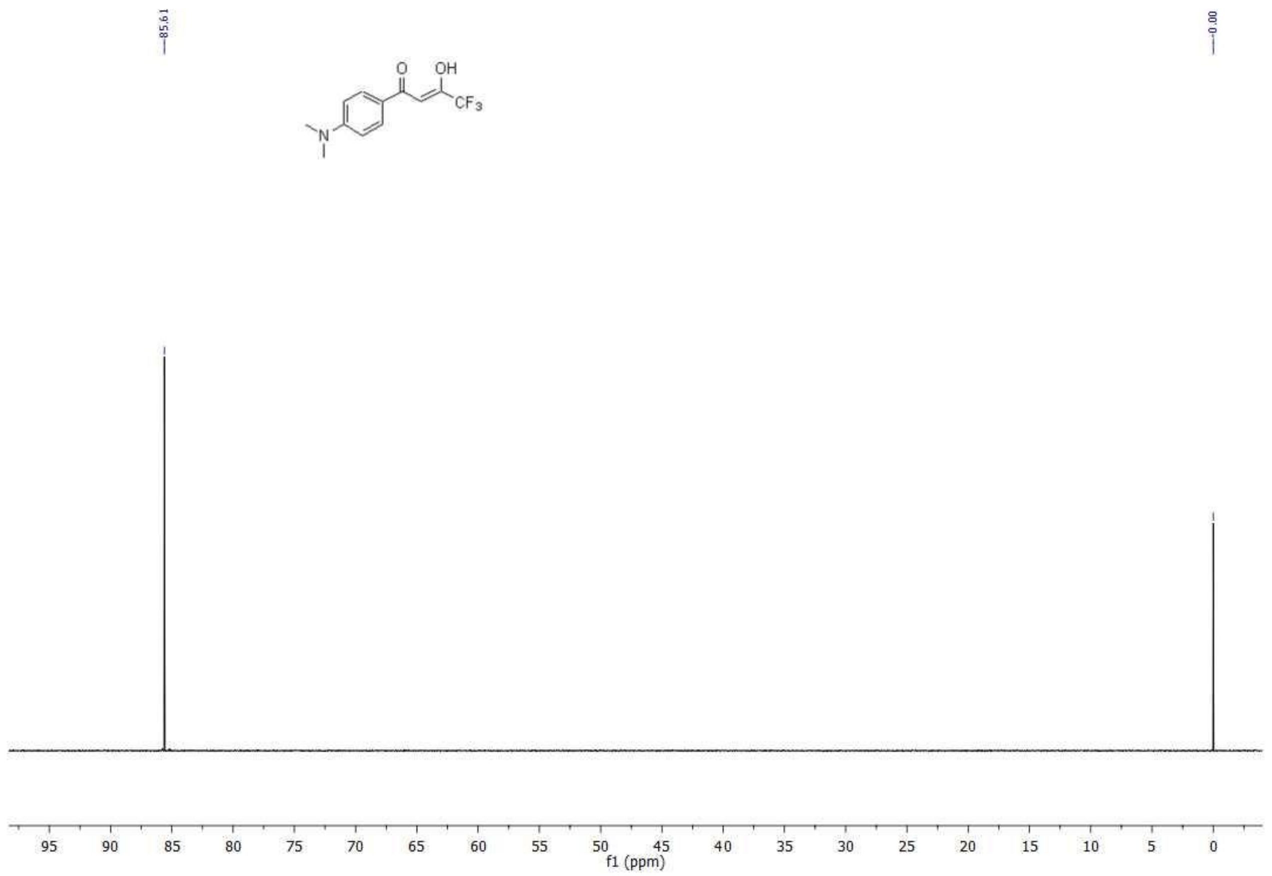

Figure S2. ^{19}F NMR (471 MHz, $CDCl_3$) spectrum of **3**.

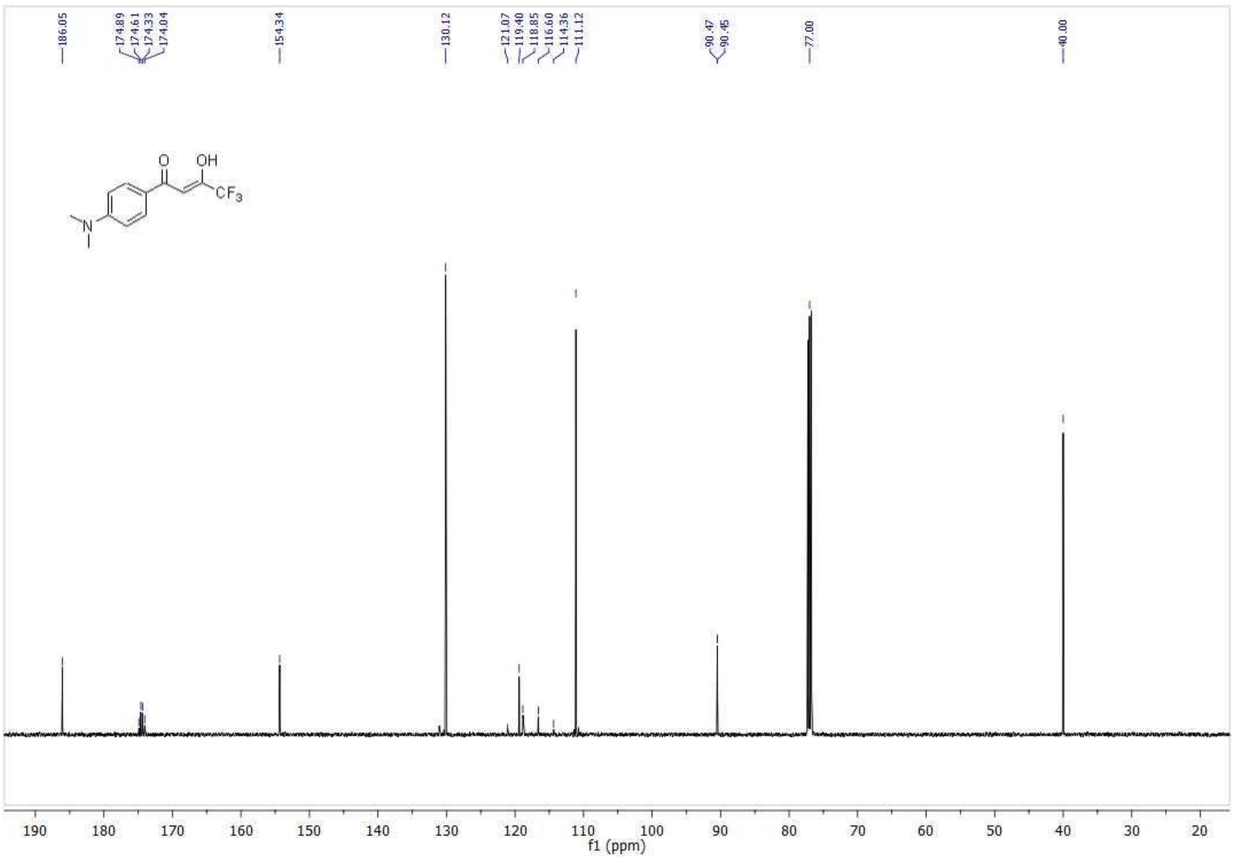

Figure S3. ^{13}C NMR (126 MHz, $CDCl_3$) spectrum of **3**.

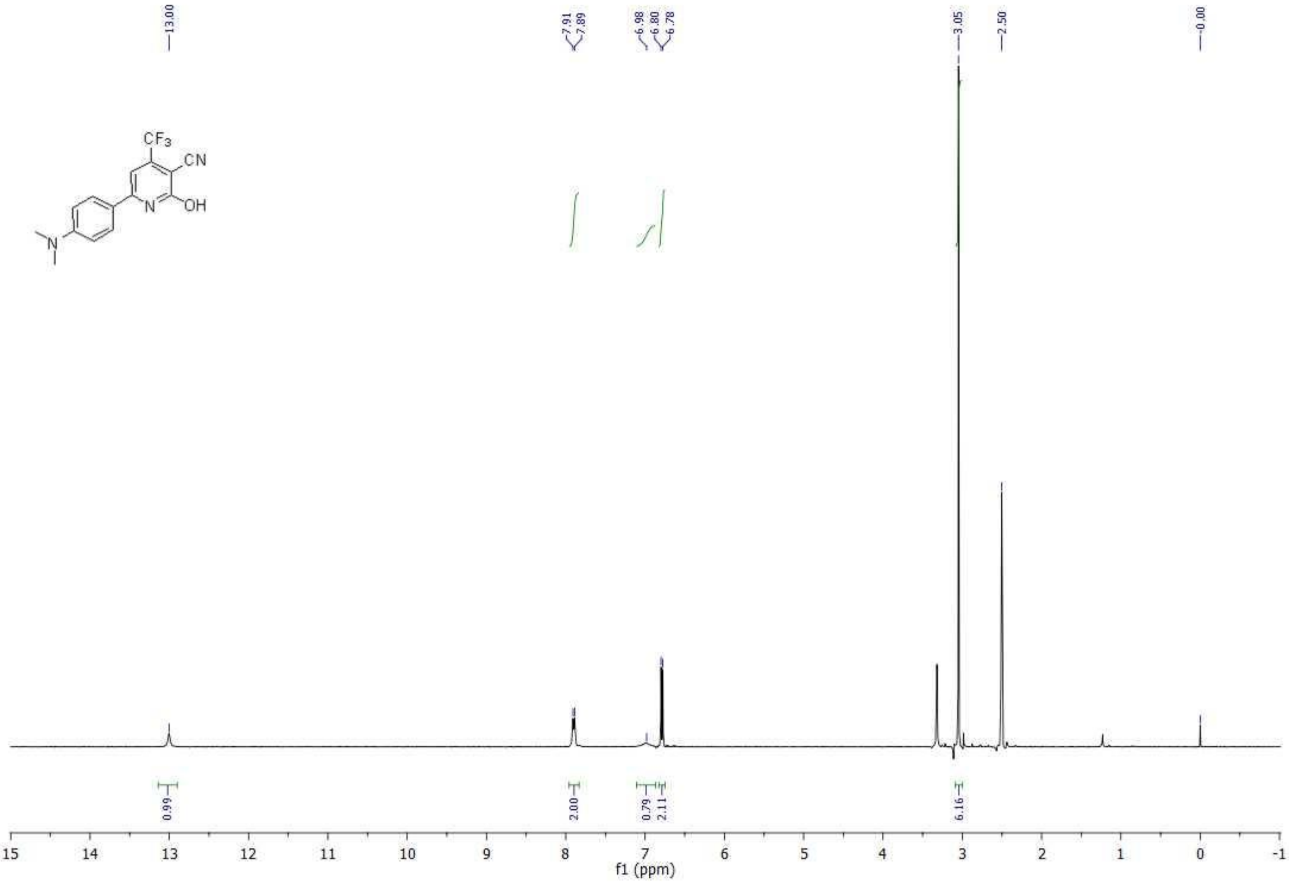

Figure S4. ^{1}H NMR (500 MHz, DMSO-d_6) spectrum of **4**.

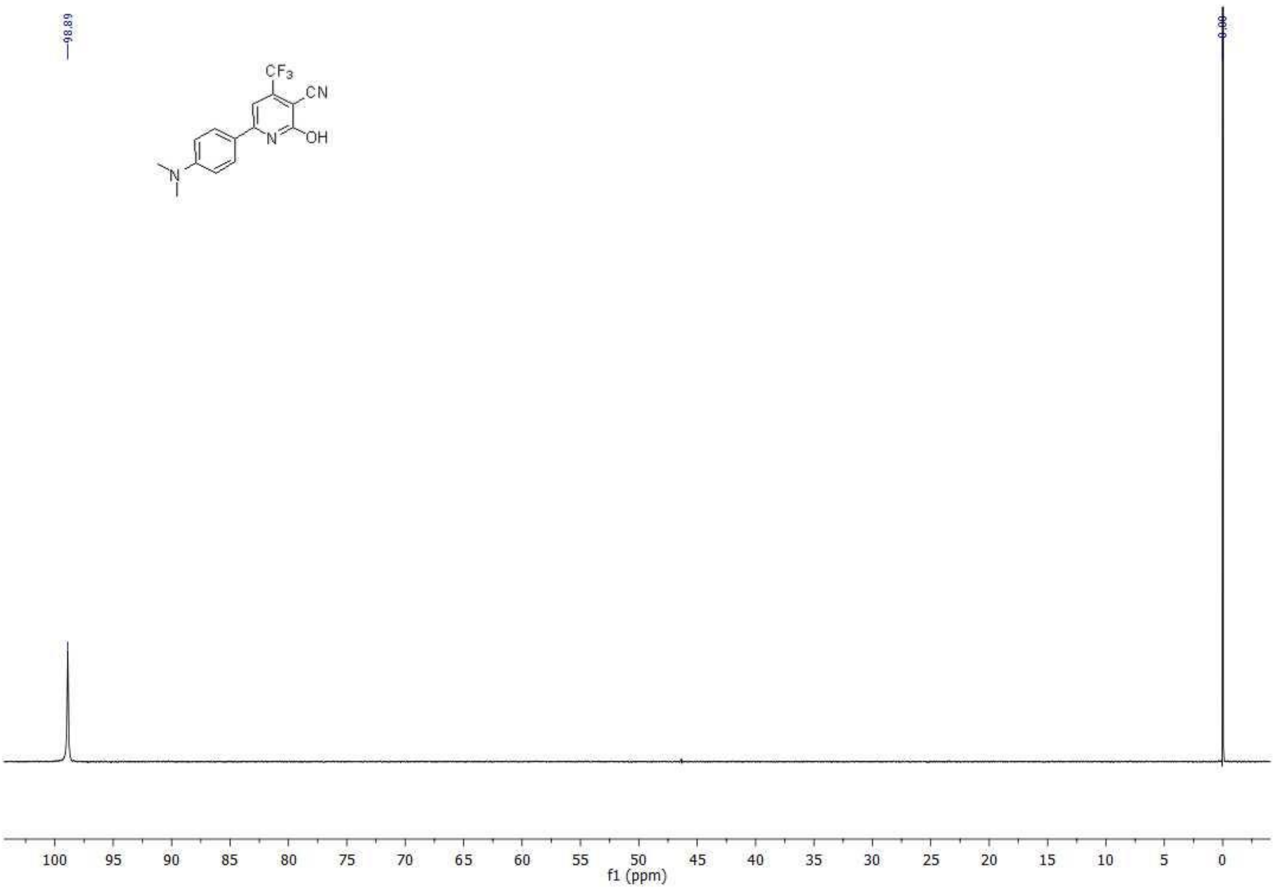

Figure S5. ^{19}F NMR (471 MHz, DMSO-d_6) spectrum of **4**.

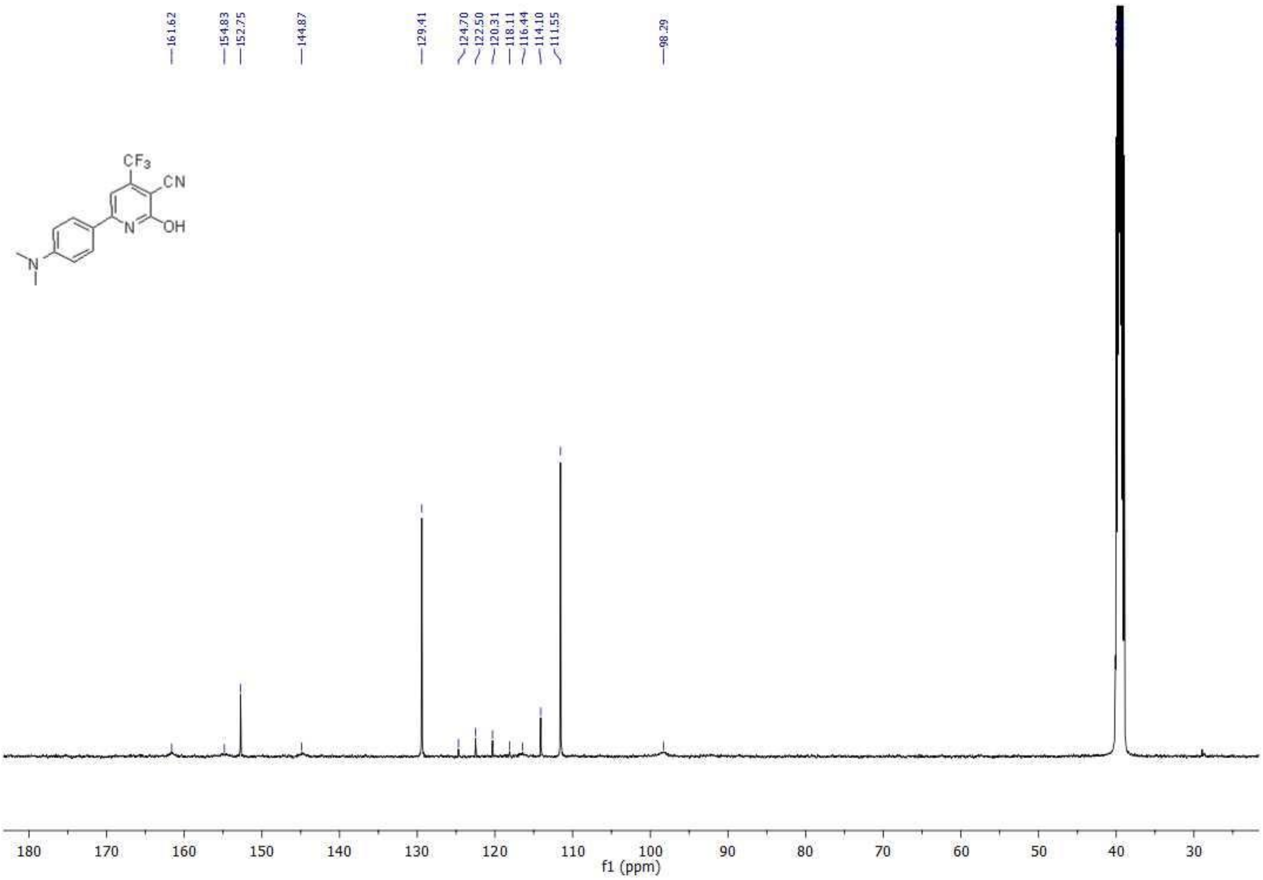

Figure S6. ^{13}C NMR (126 MHz, DMSO-d_6) spectrum of **4**.

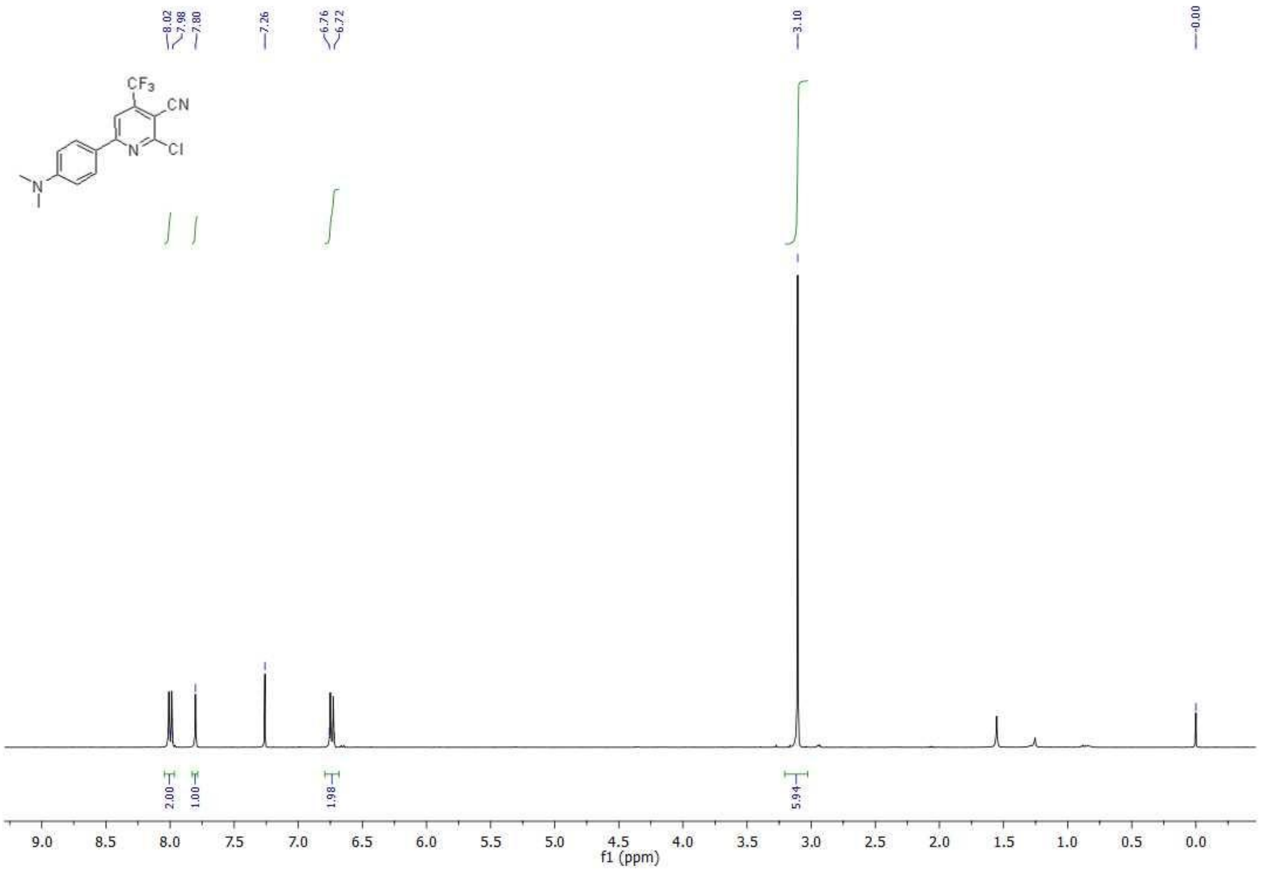

Figure S7. ^{1}H NMR (500 MHz, $CDCl_3$) spectrum of **5**.

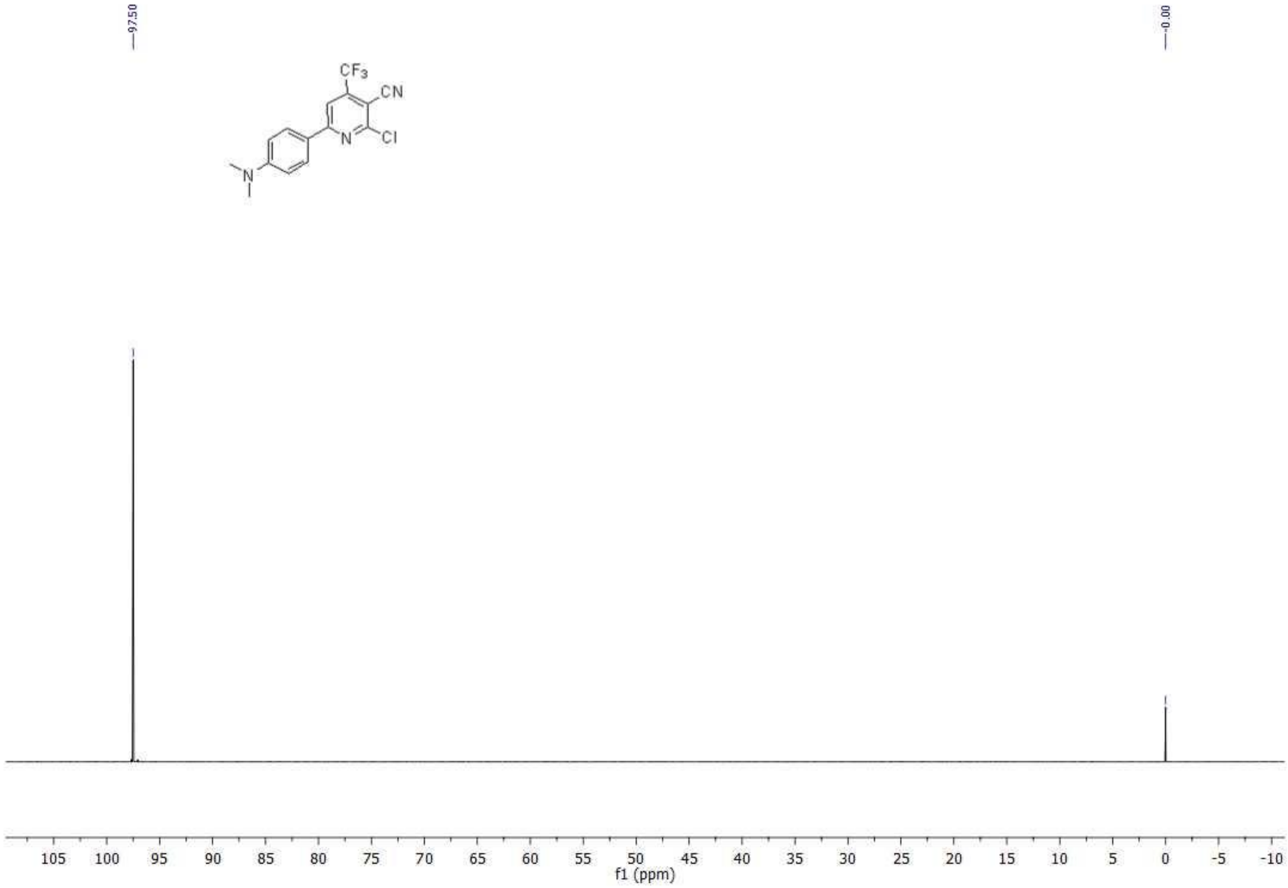

Figure S8. ^{19}F NMR (471 MHz, $CDCl_3$) spectrum of **5**.

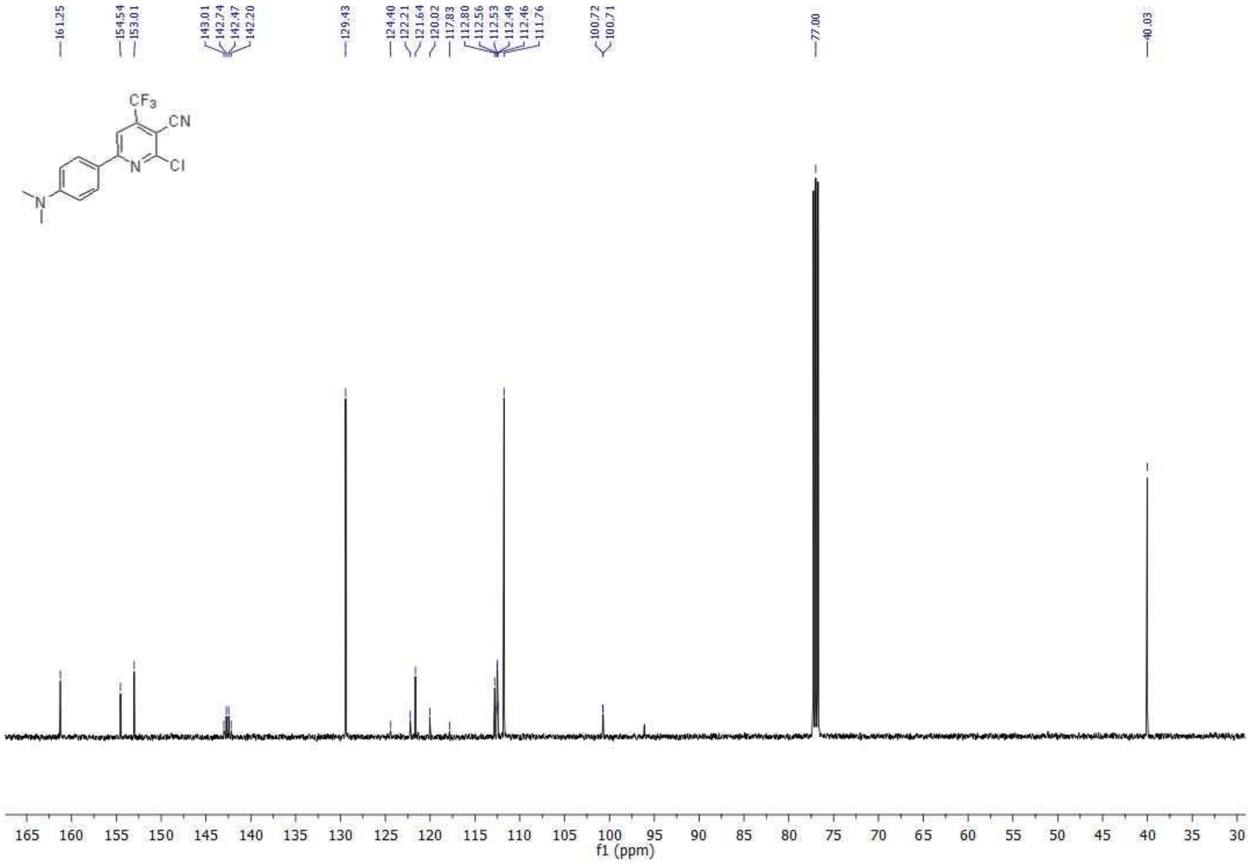

Figure S9. ^{13}C NMR (126 MHz, $CDCl_3$) spectrum of **5**.

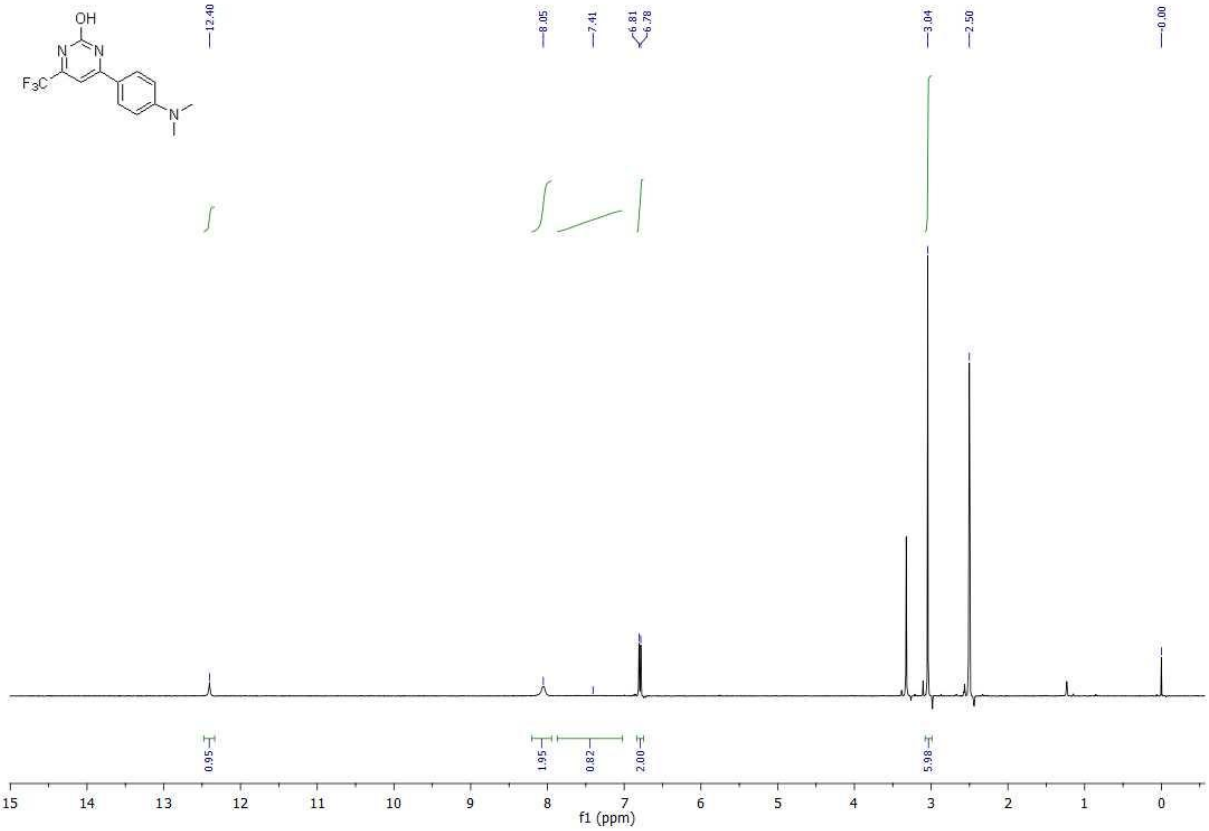

Figure S10. ^{1}H NMR (500 MHz, DMSO-d_6) spectrum of **6**.

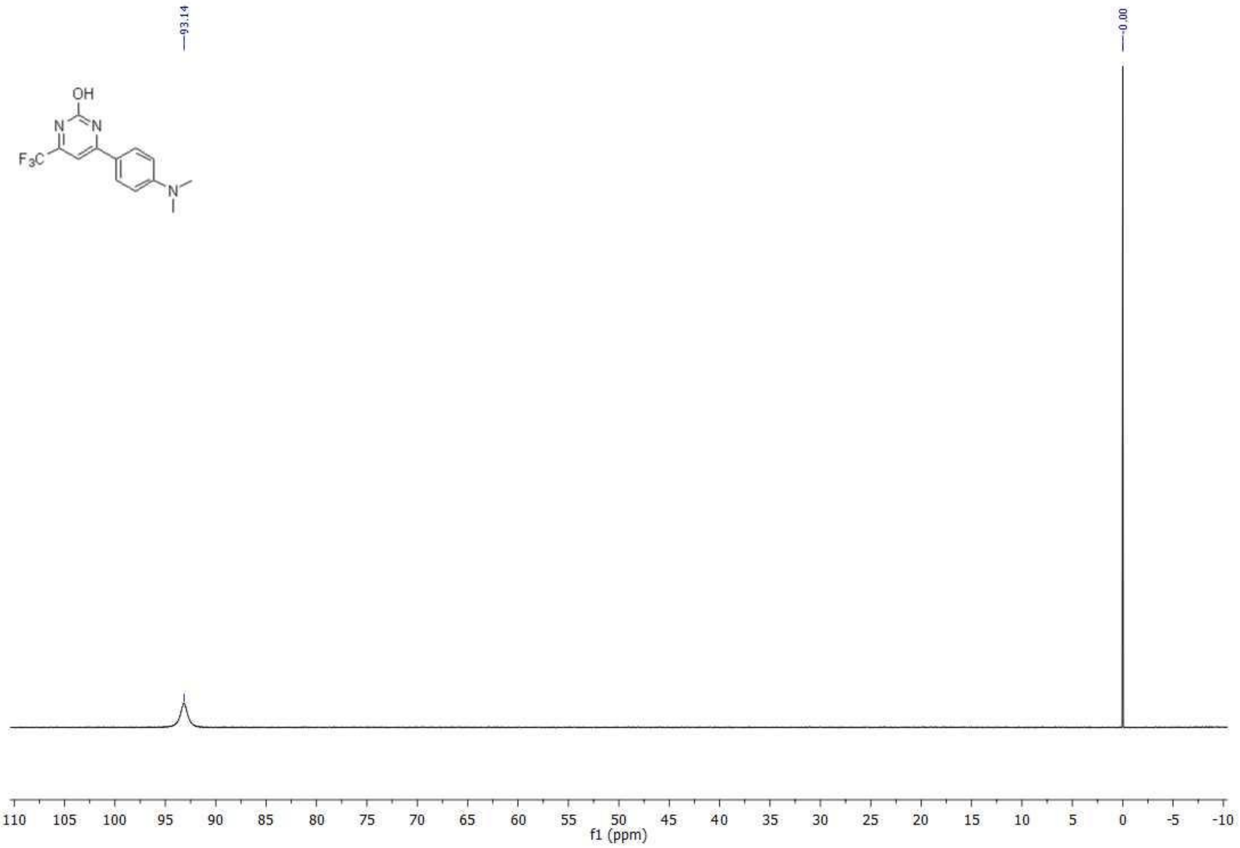

Figure S11. ^{19}F NMR (471 MHz, DMSO-d_6) spectrum of **6**.

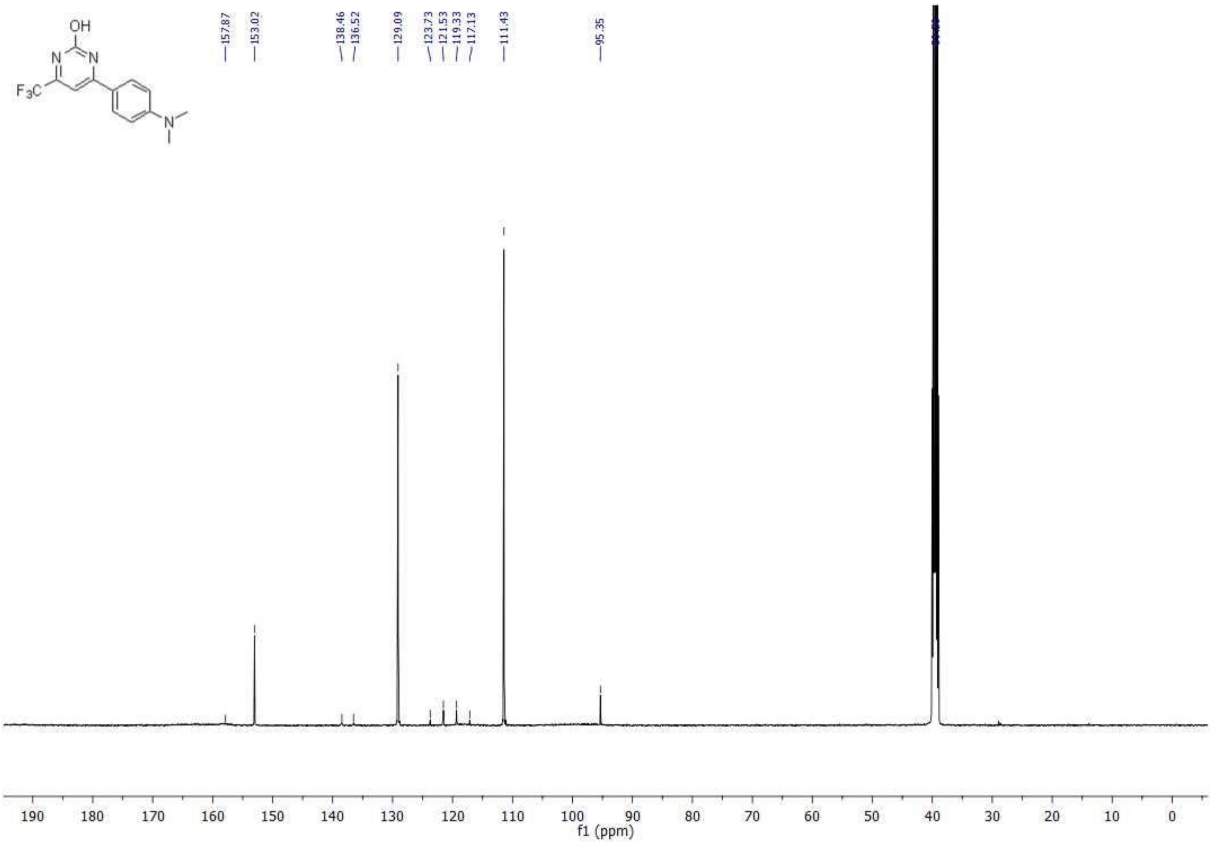

Figure S12. ^{13}C NMR (126 MHz, DMSO-d_6) spectrum of **6**.

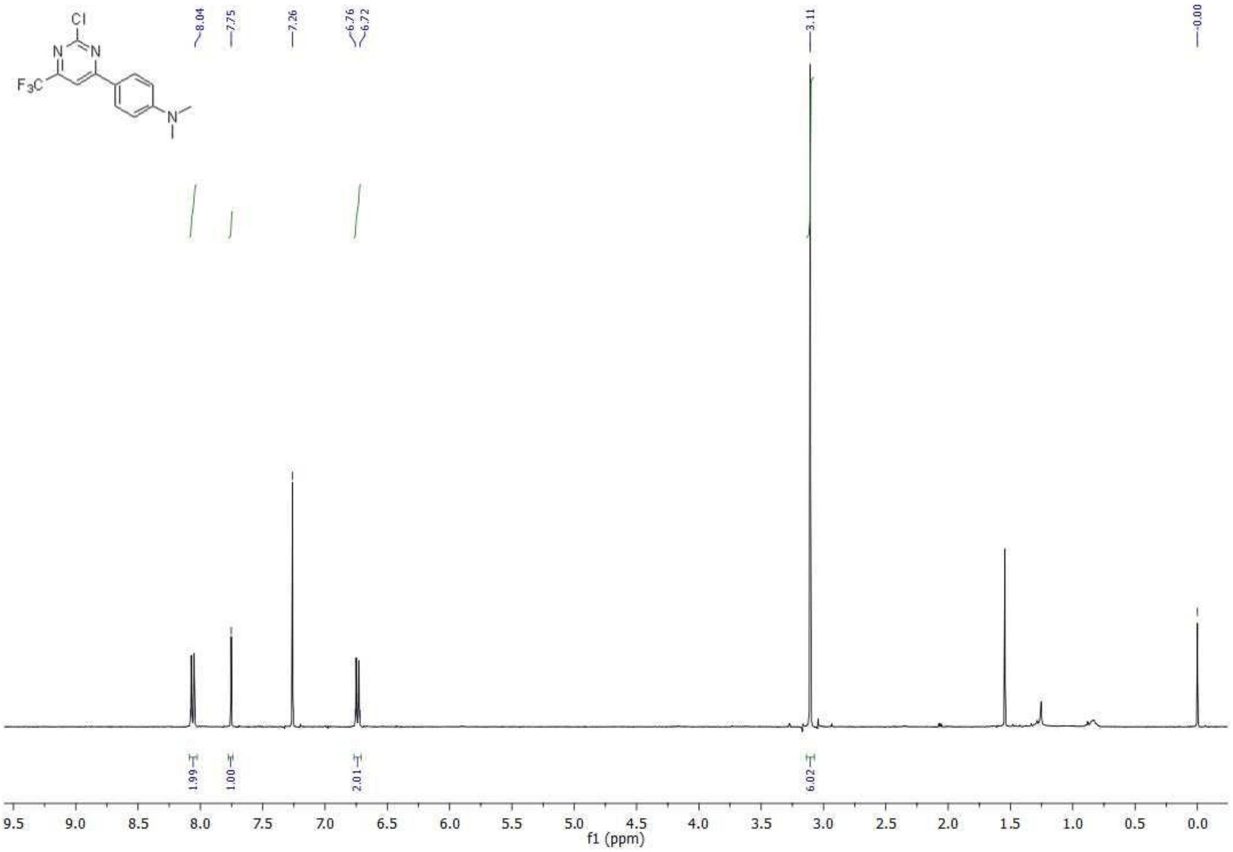

Figure S13. ^{1}H NMR (500 MHz, $CDCl_3$) spectrum of **7**.

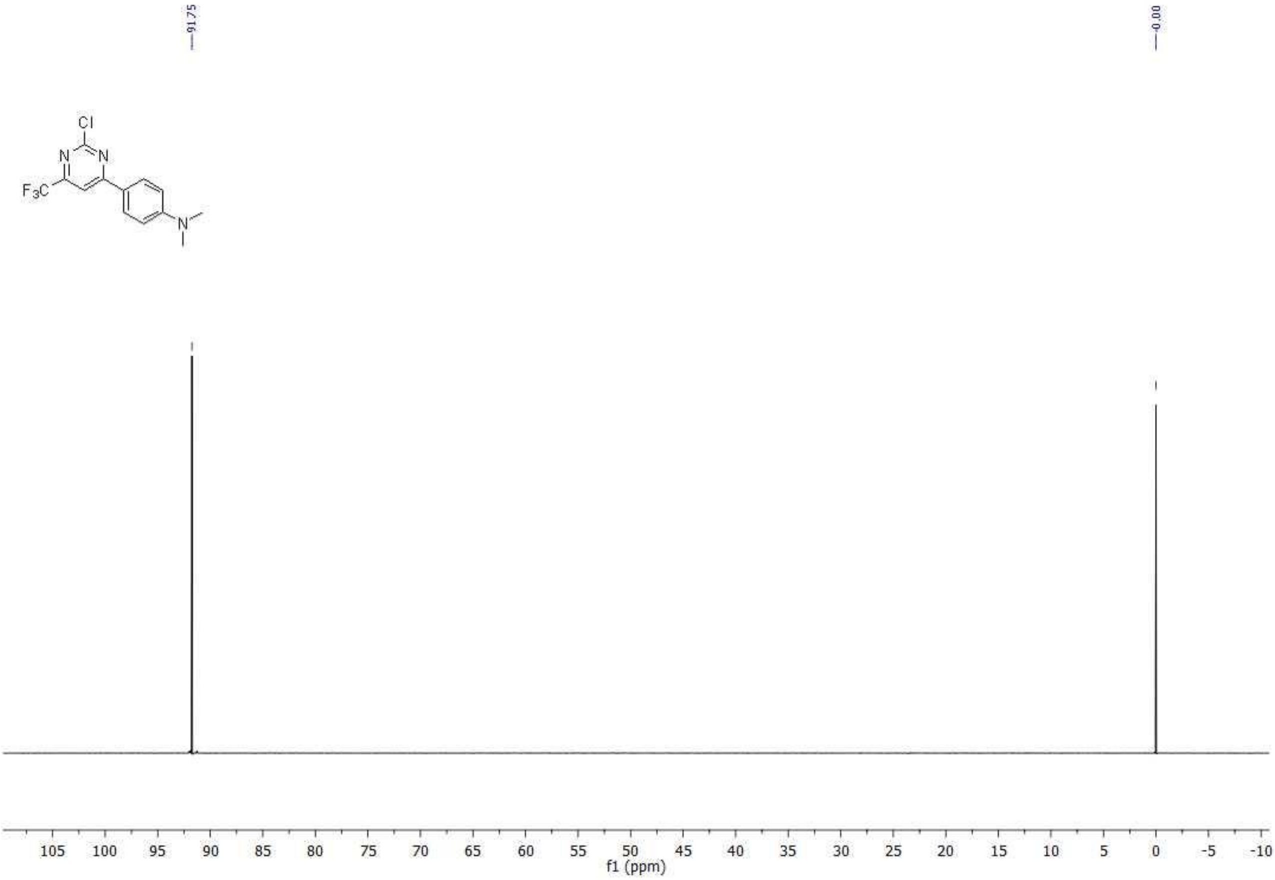

Figure S14. ^{19}F NMR (471 MHz, $CDCl_3$) spectrum of **7**.

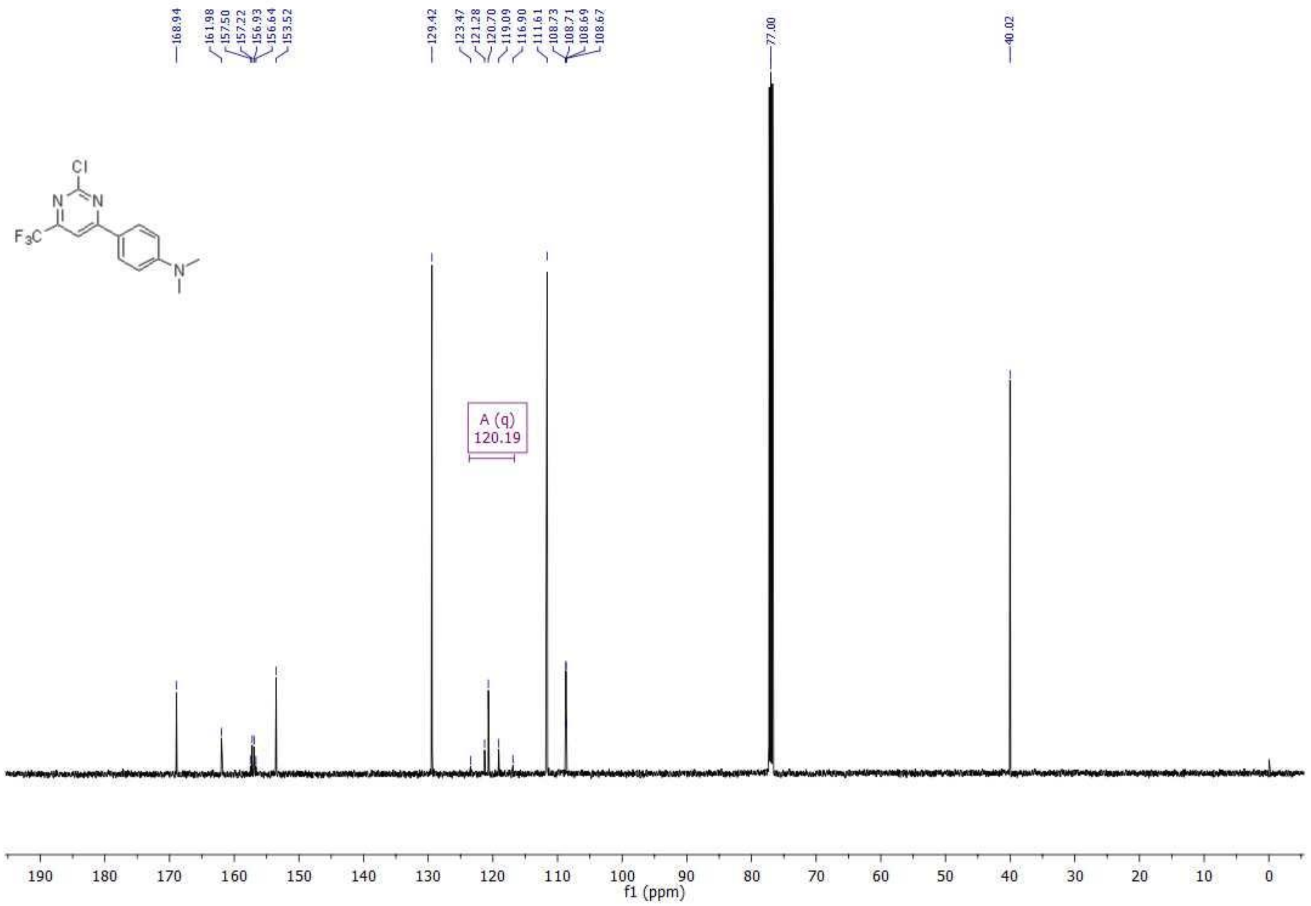

Figure S15. ^{13}C NMR (126 MHz, $CDCl_3$) spectrum of **7**.

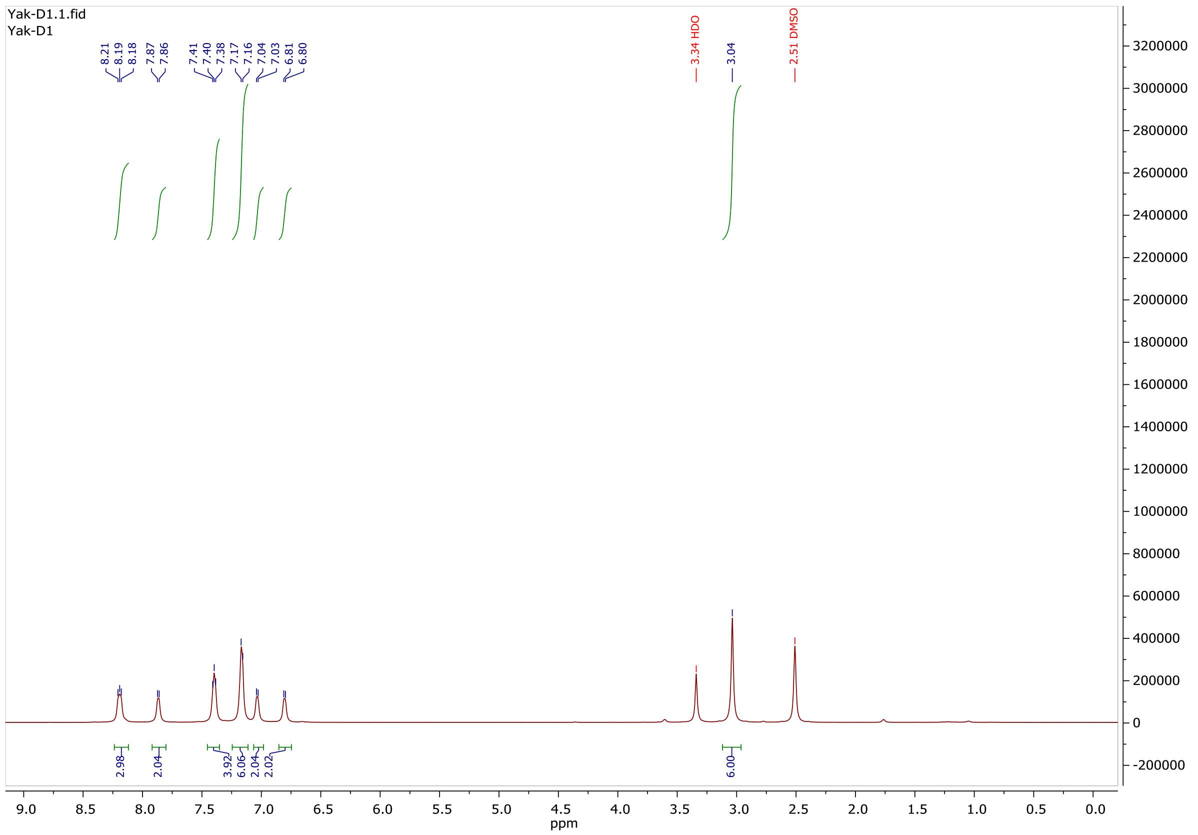

Figure S16. ^{1}H NMR (600 MHz, DMSO-d_6) spectrum of **9a**.

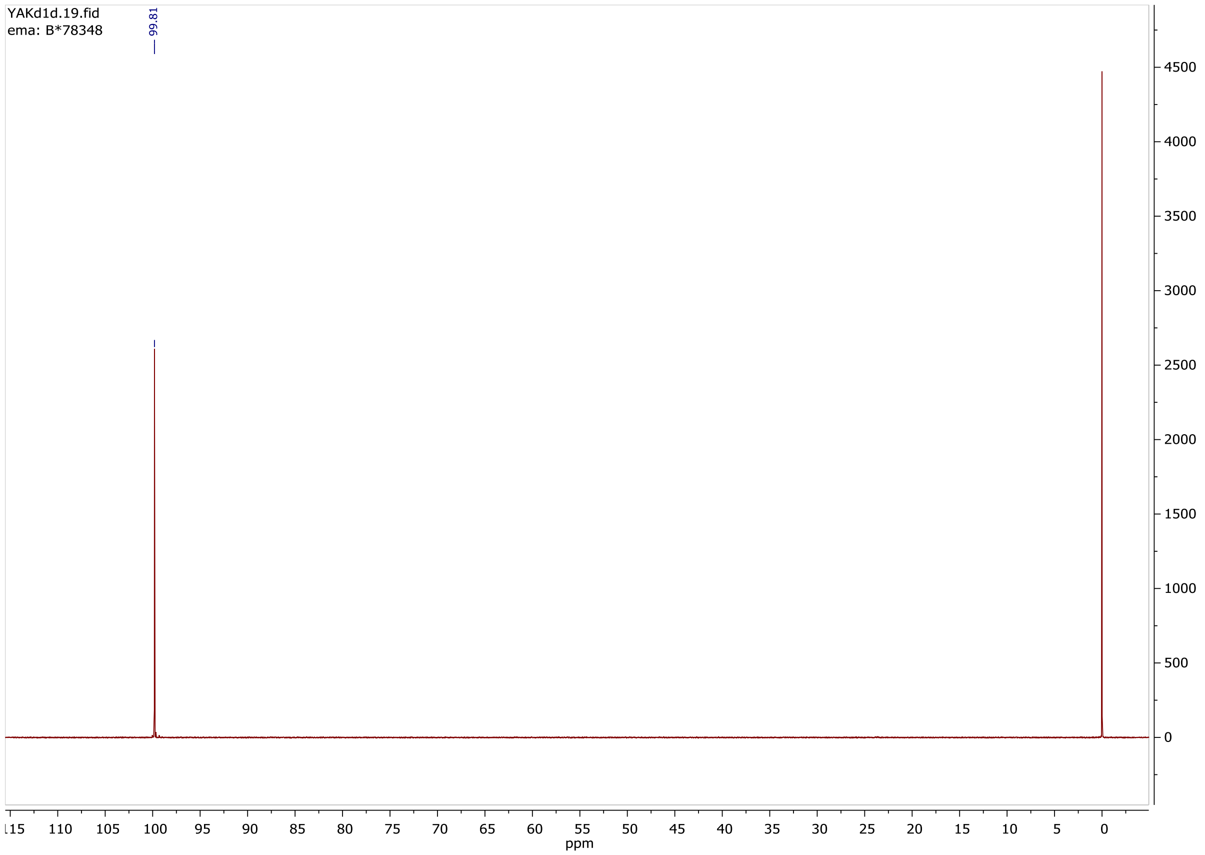

Figure S17. ^{19}F NMR (376 MHz, DMSO-d_6) spectrum of **9a**.

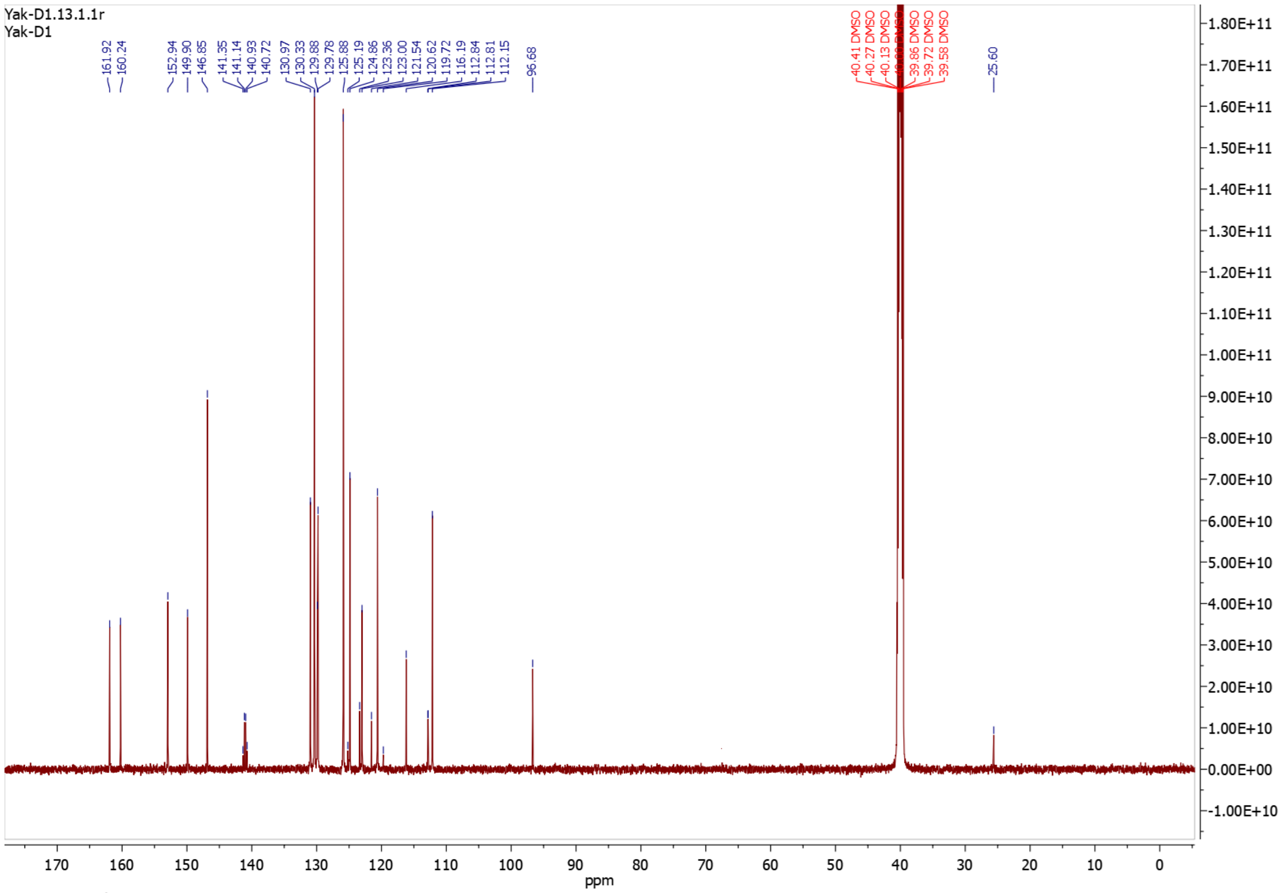

Figure S18. ^{13}C NMR (151 MHz, DMSO-d_6) spectrum of **9a**.

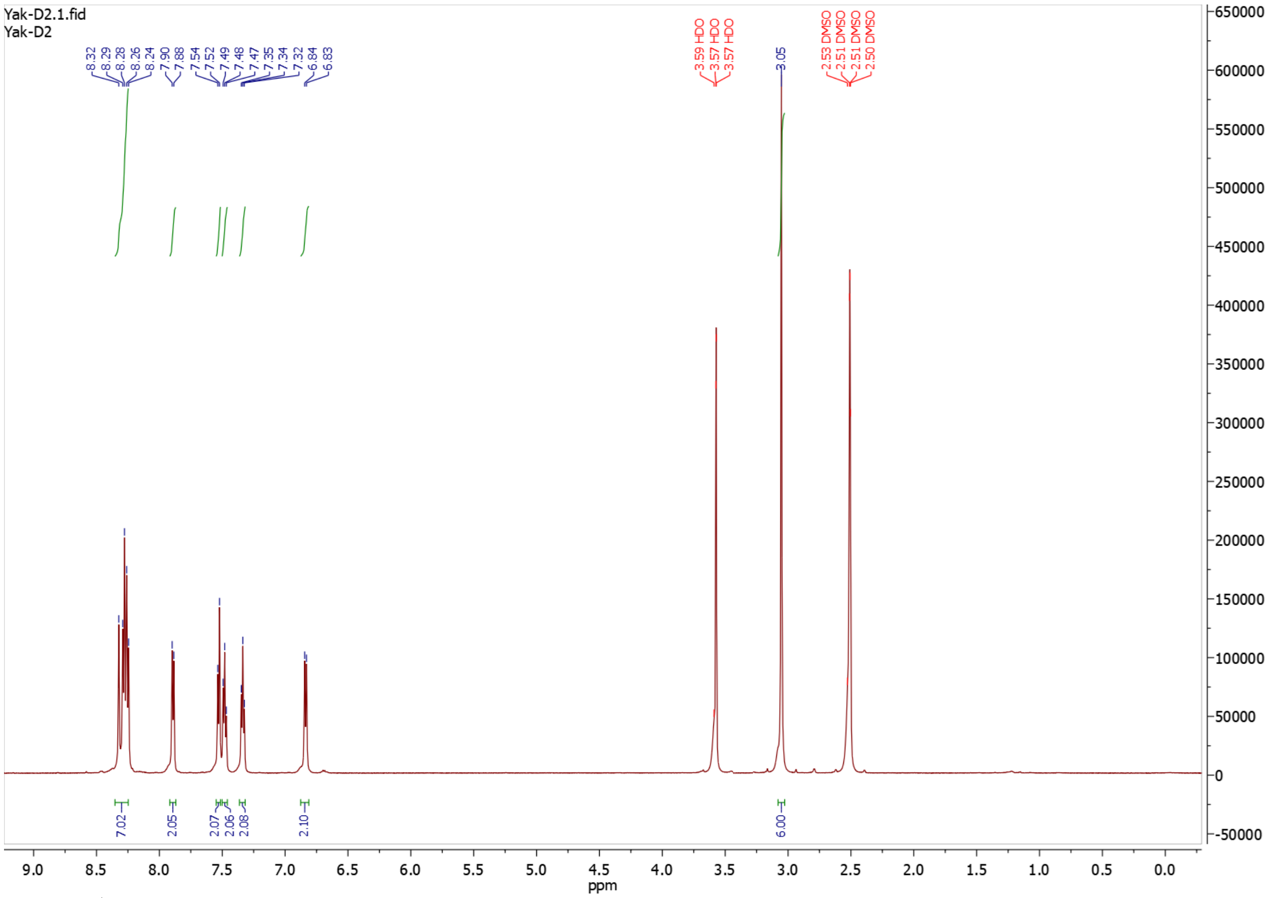

Figure S19. ^{1}H NMR (600 MHz, DMSO-d_6) spectrum of **9b**.

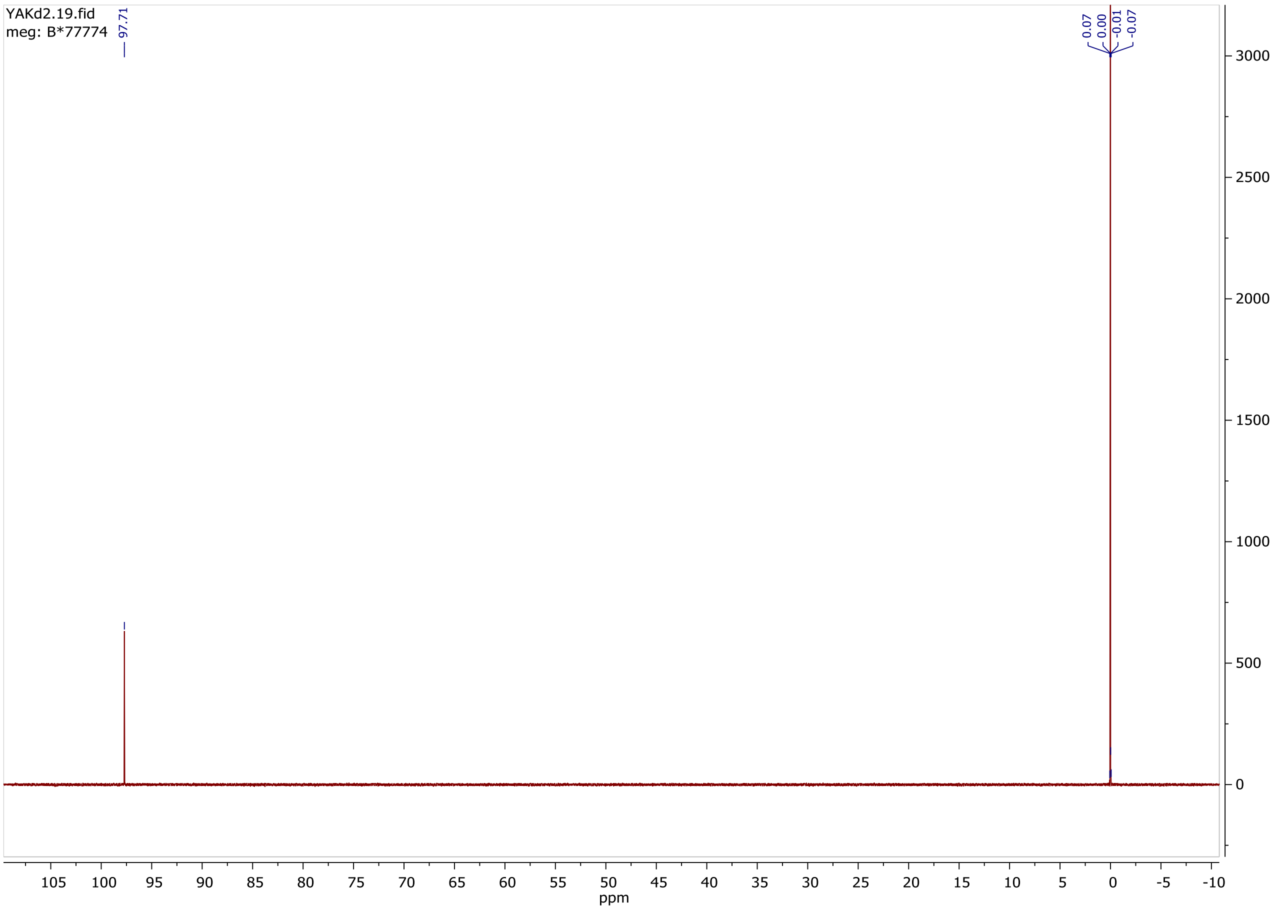

Figure S20. ^{19}F NMR (376 MHz, $CDCl_3$) spectrum of **9b**.

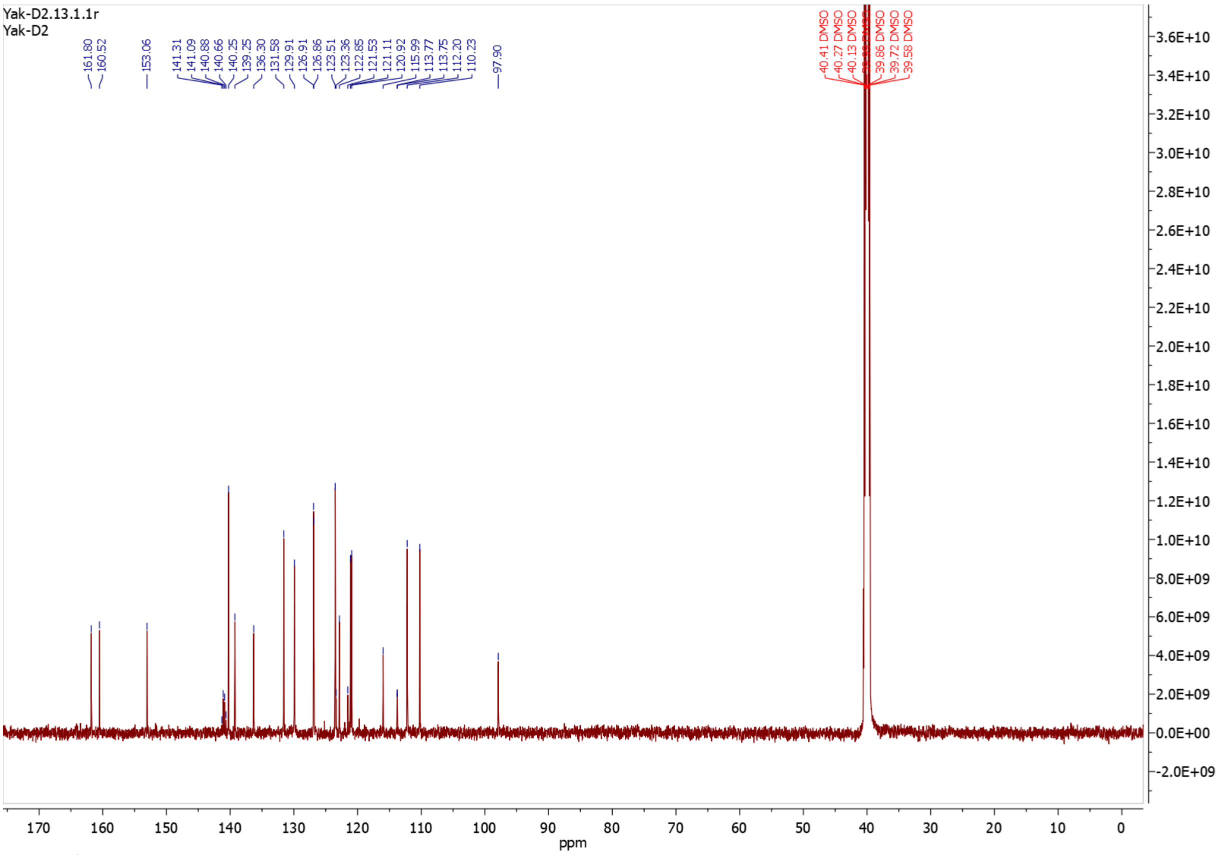

Figure S21. ^{13}C NMR (151 MHz, DMSO-d_6) spectrum of **9b**.

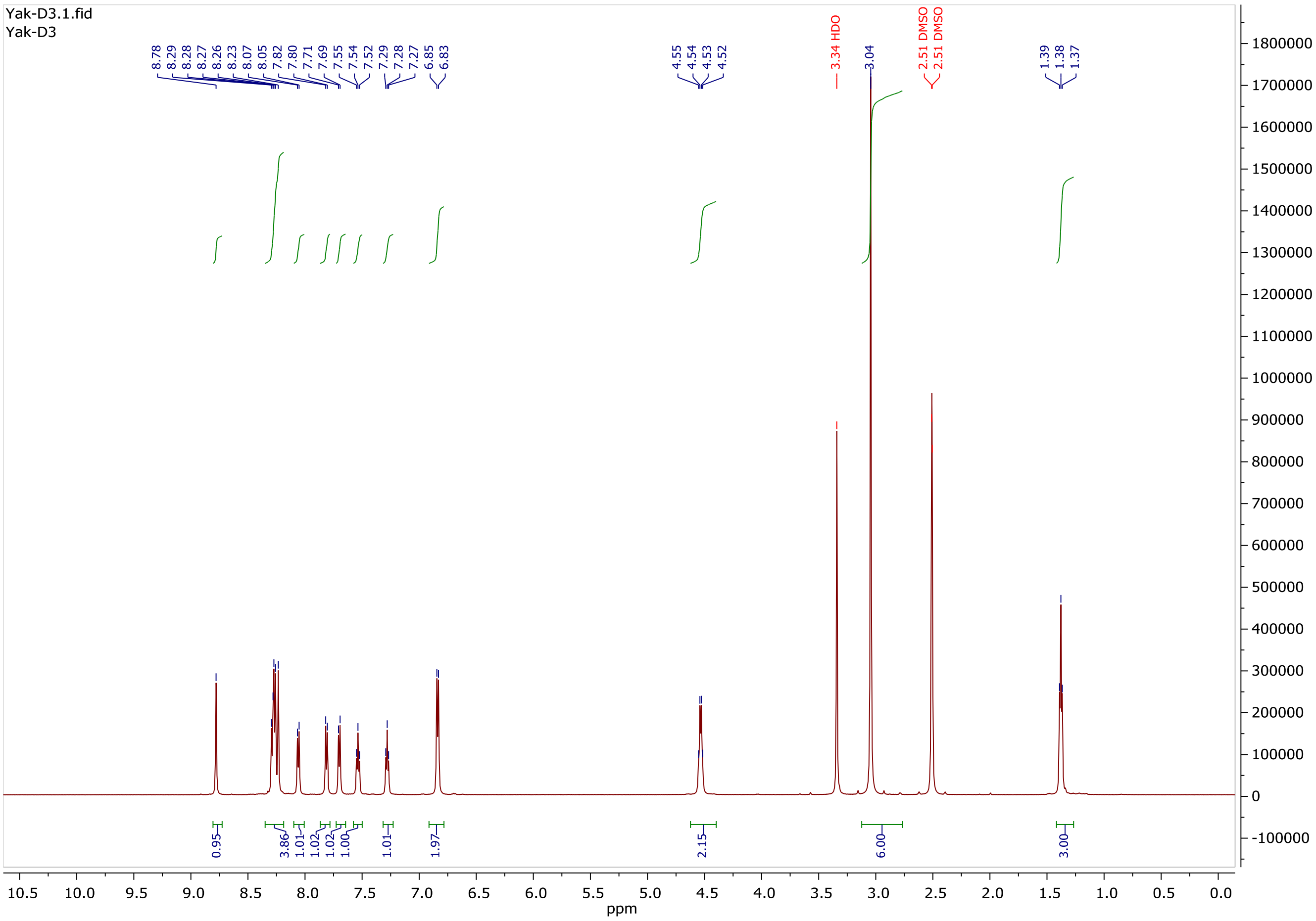

Figure S22. ^{1}H NMR (600 MHz, DMSO-d_6) spectrum of **9c**.

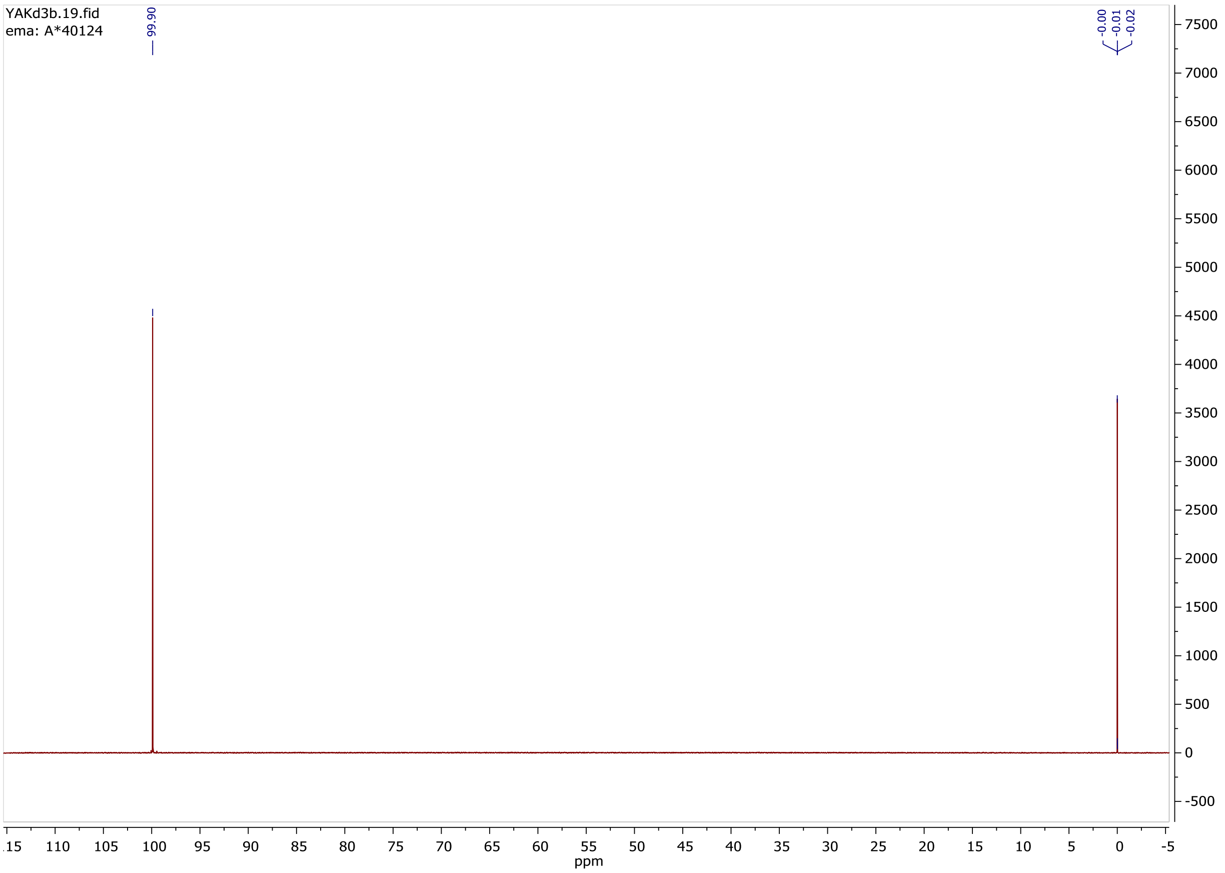

Figure S23. ^{19}F NMR (471 MHz, DMSO-d_6) spectrum of **9c**.

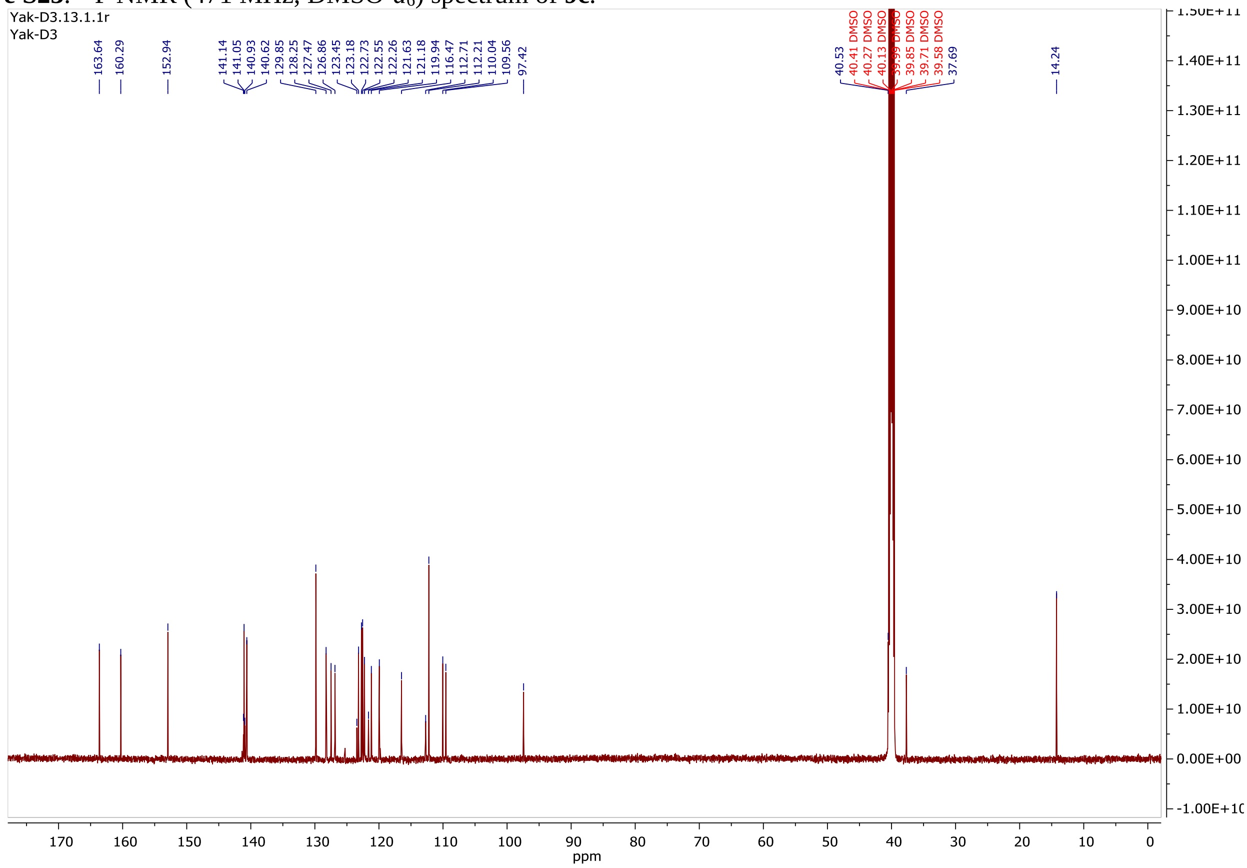

Figure S24. ^{13}C NMR (151 MHz, DMSO-d_6) spectrum of **9c**.

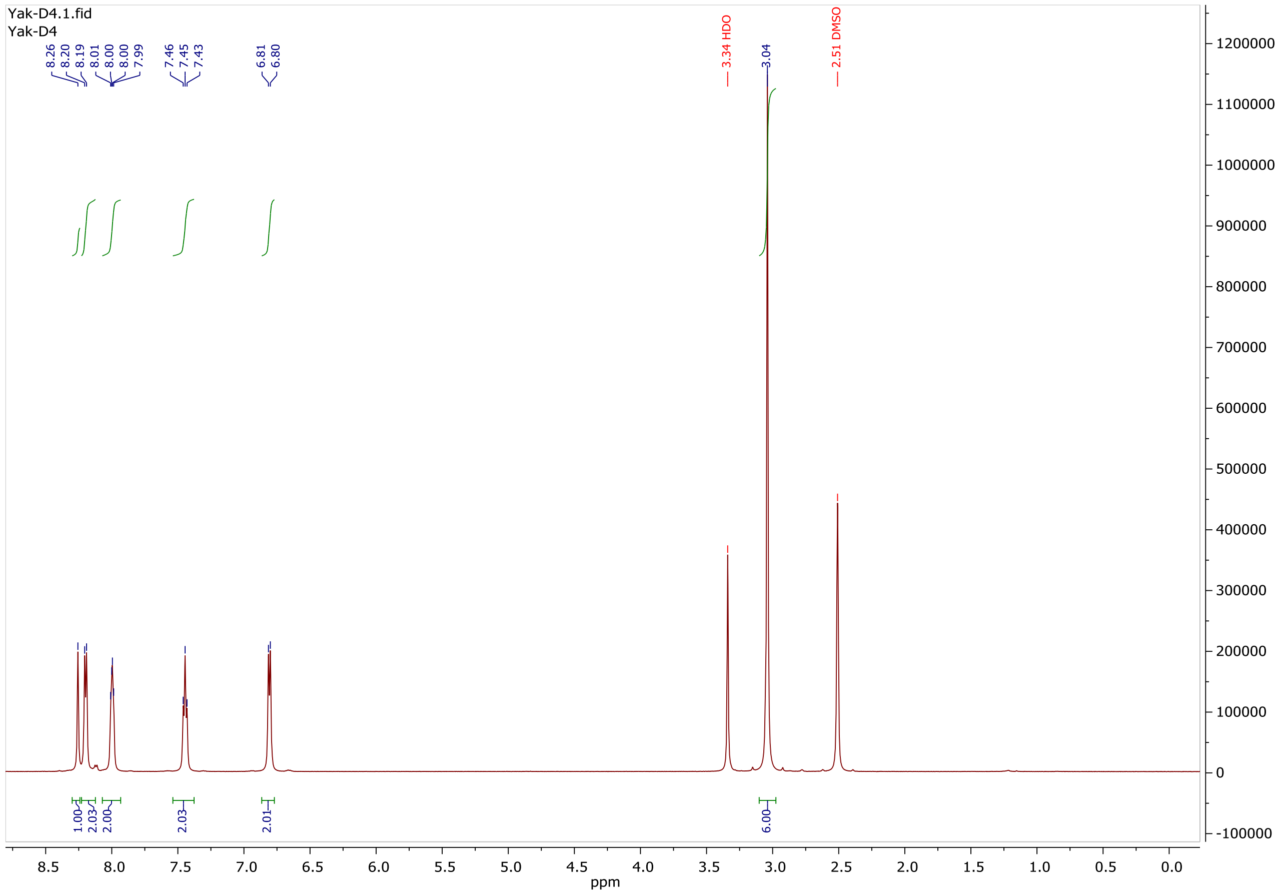

Figure S25. ^{1}H NMR (600 MHz, DMSO-d_6) spectrum of **9d**.

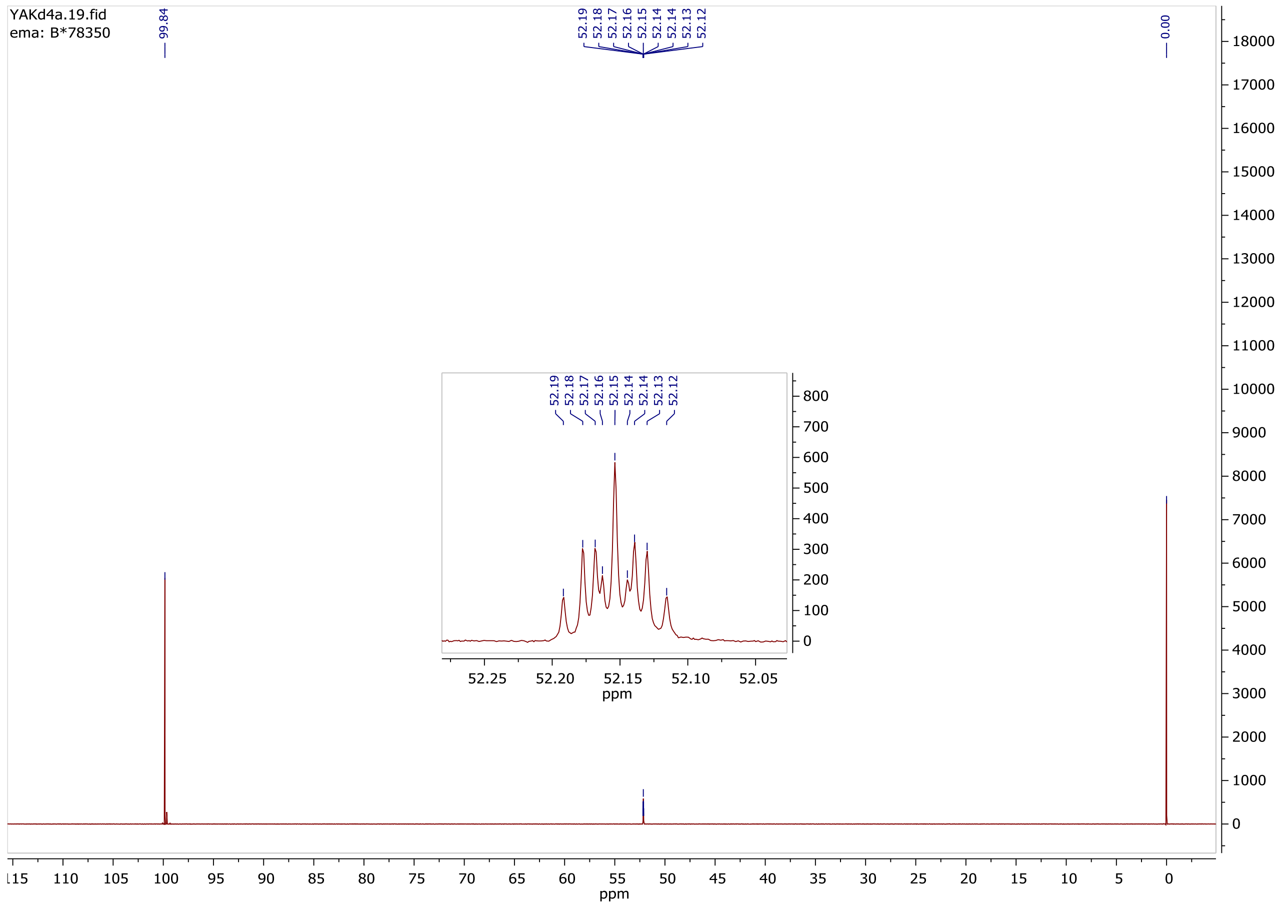

Figure S26. ^{19}F NMR (376 MHz, DMSO-d_6) spectrum of **9d**.

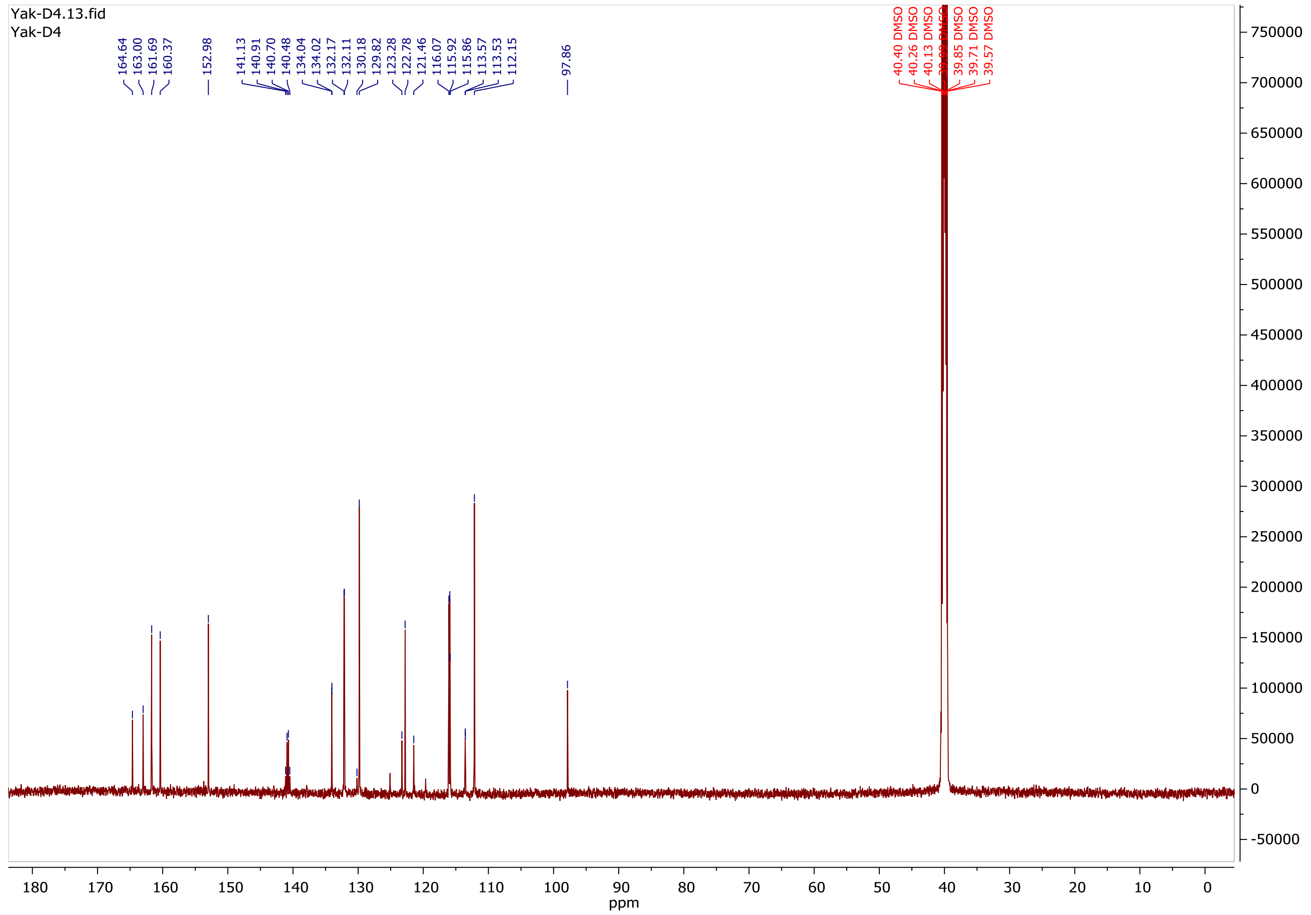

Figure S27. ^{13}C NMR (151 MHz, DMSO-d_6) spectrum of **9d**.

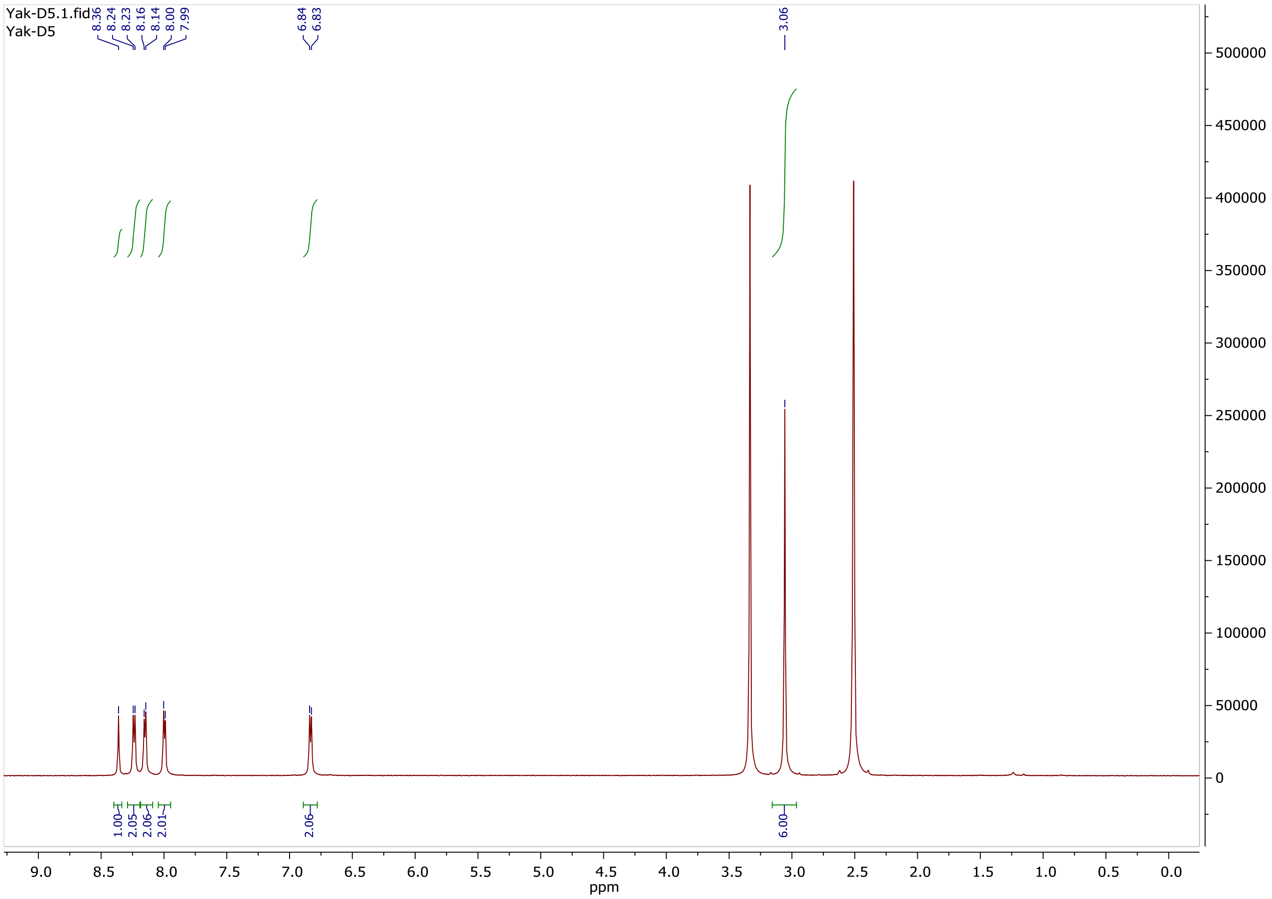

Figure S28. ^{1}H NMR (600 MHz, DMSO-d_6) spectrum of **9e**.

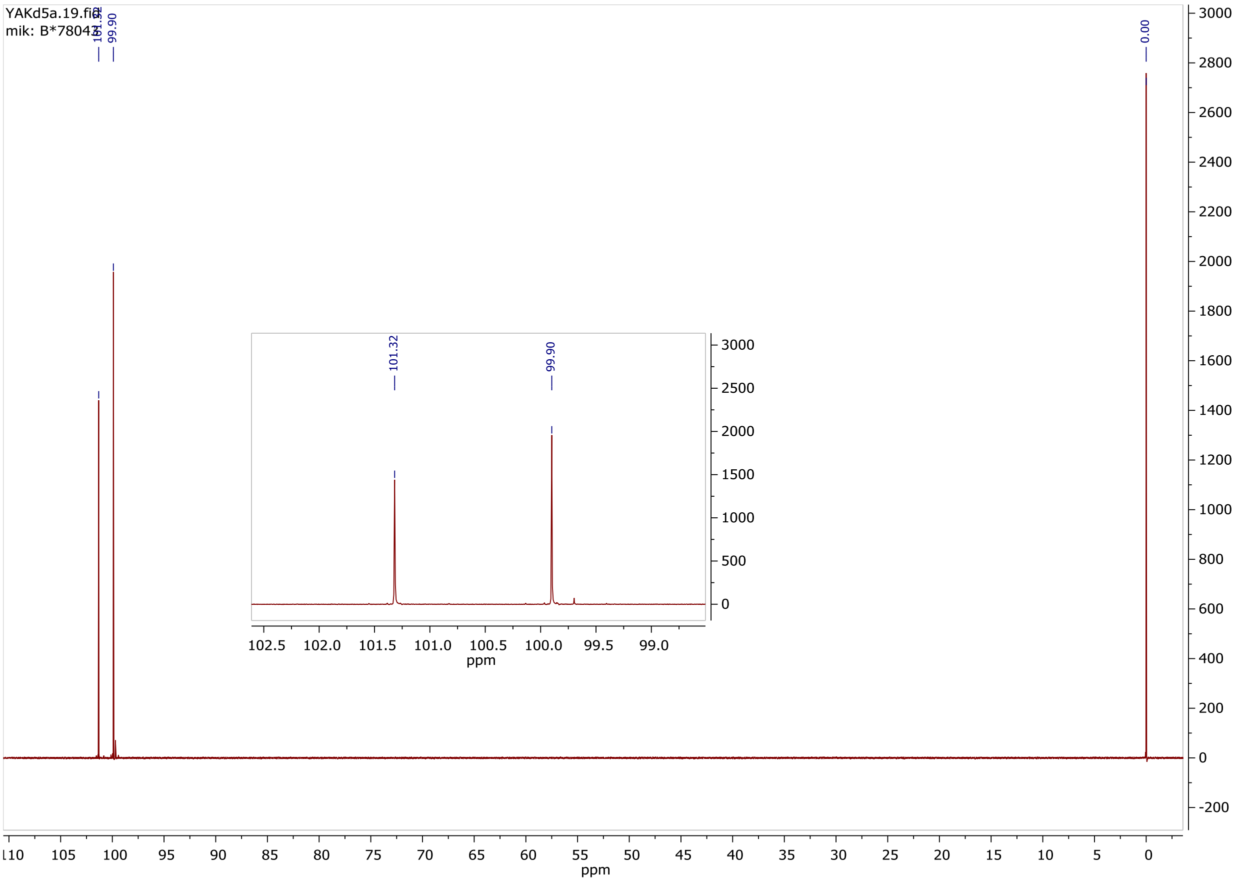

Figure S29. ^{19}F NMR (376 MHz, DMSO-d_6) spectrum of **9e**.

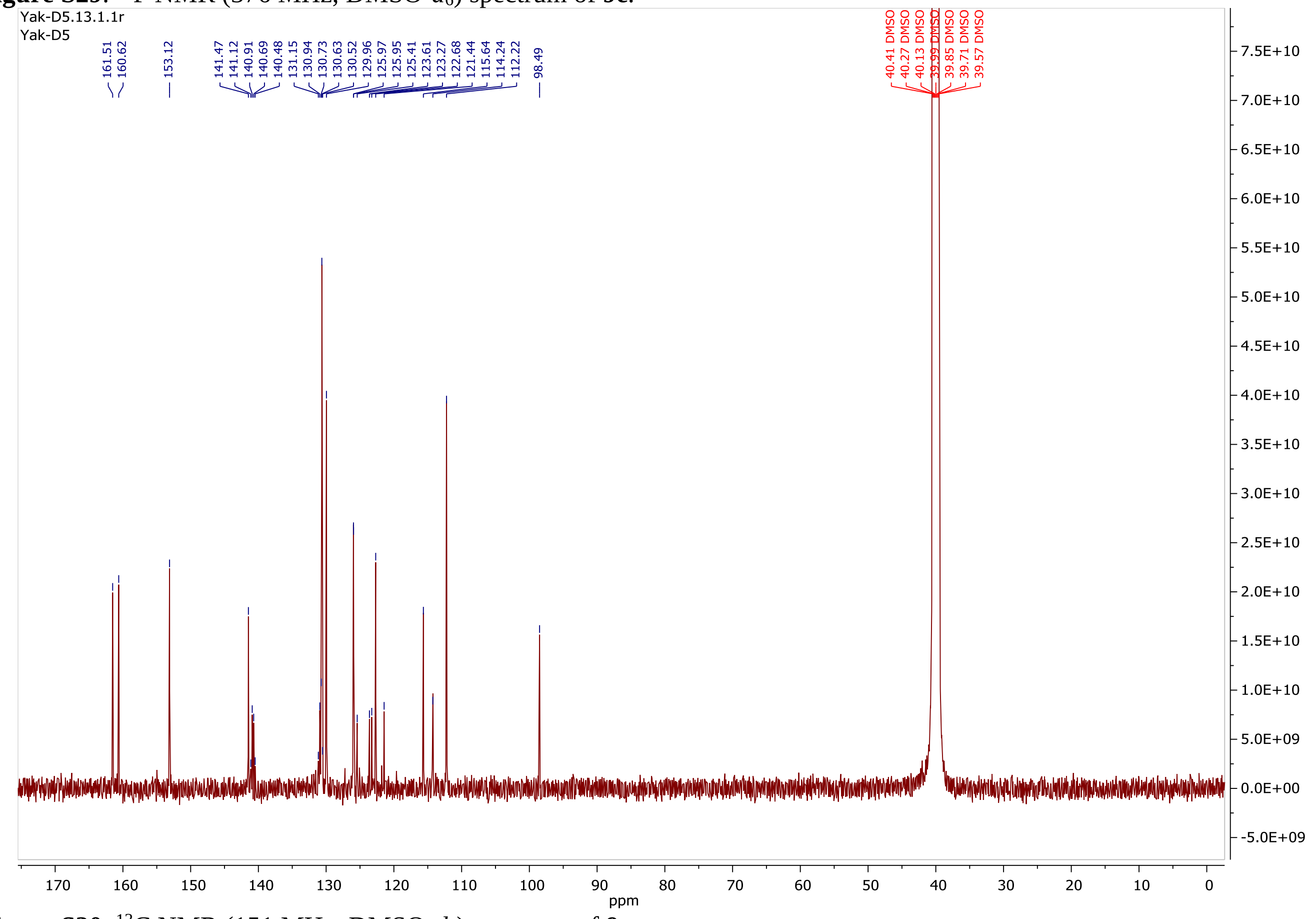

Figure S30. ^{13}C NMR (151 MHz, DMSO-d_6) spectrum of **9e**.

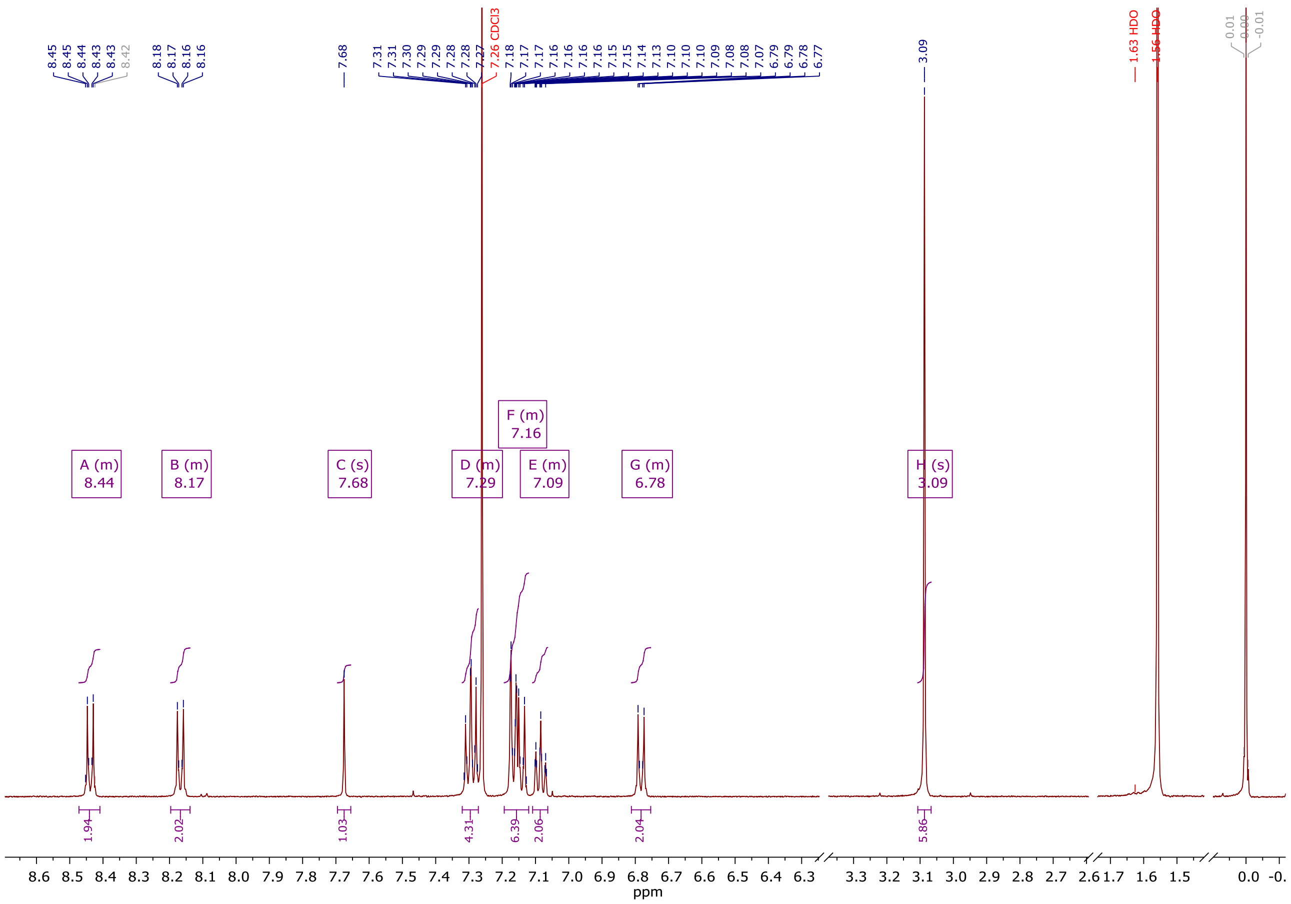

Figure S31. ^{1}H NMR (500 MHz, $CDCl_3$) spectrum of **10a**.

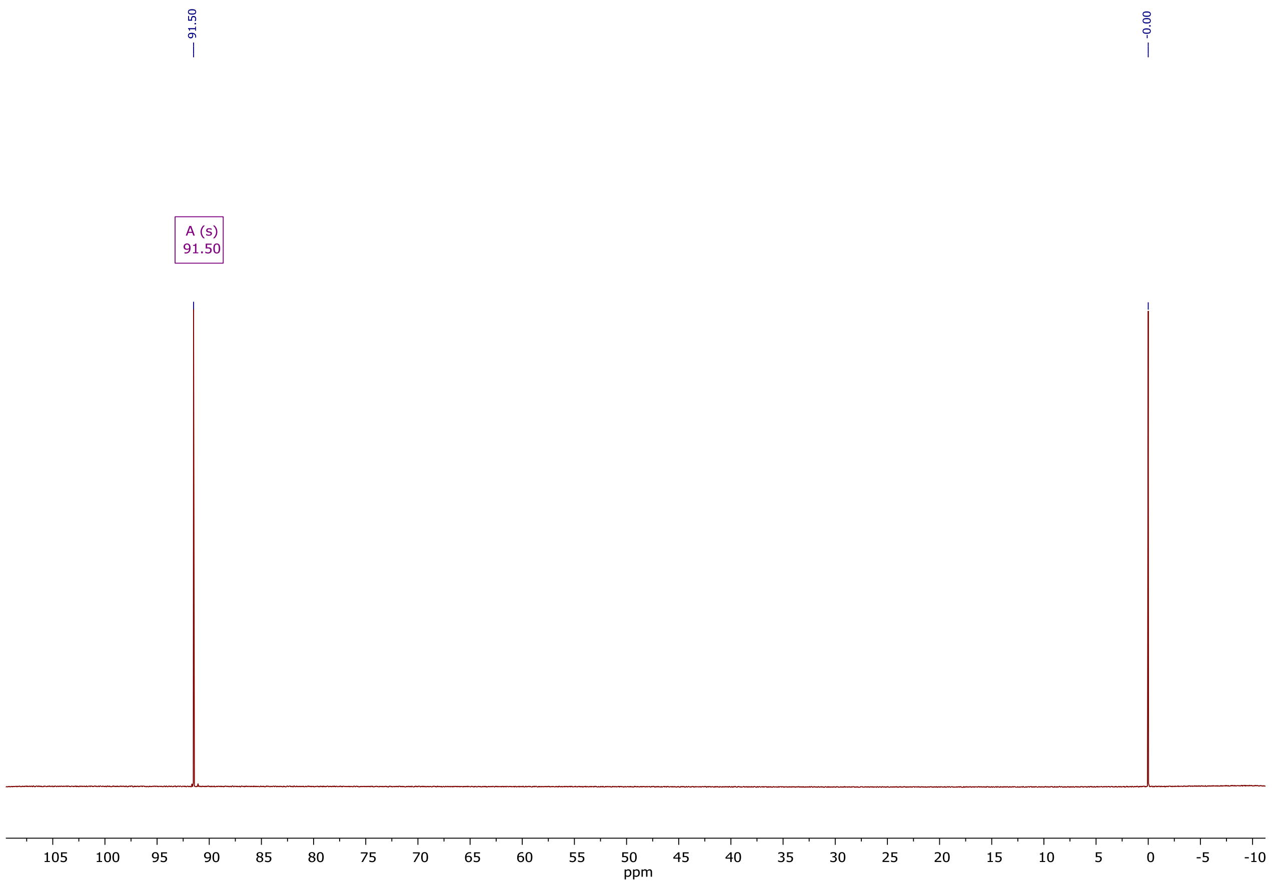

Figure S32. ^{19}F NMR (471 MHz, DMSO-d_6) spectrum of **10a**.

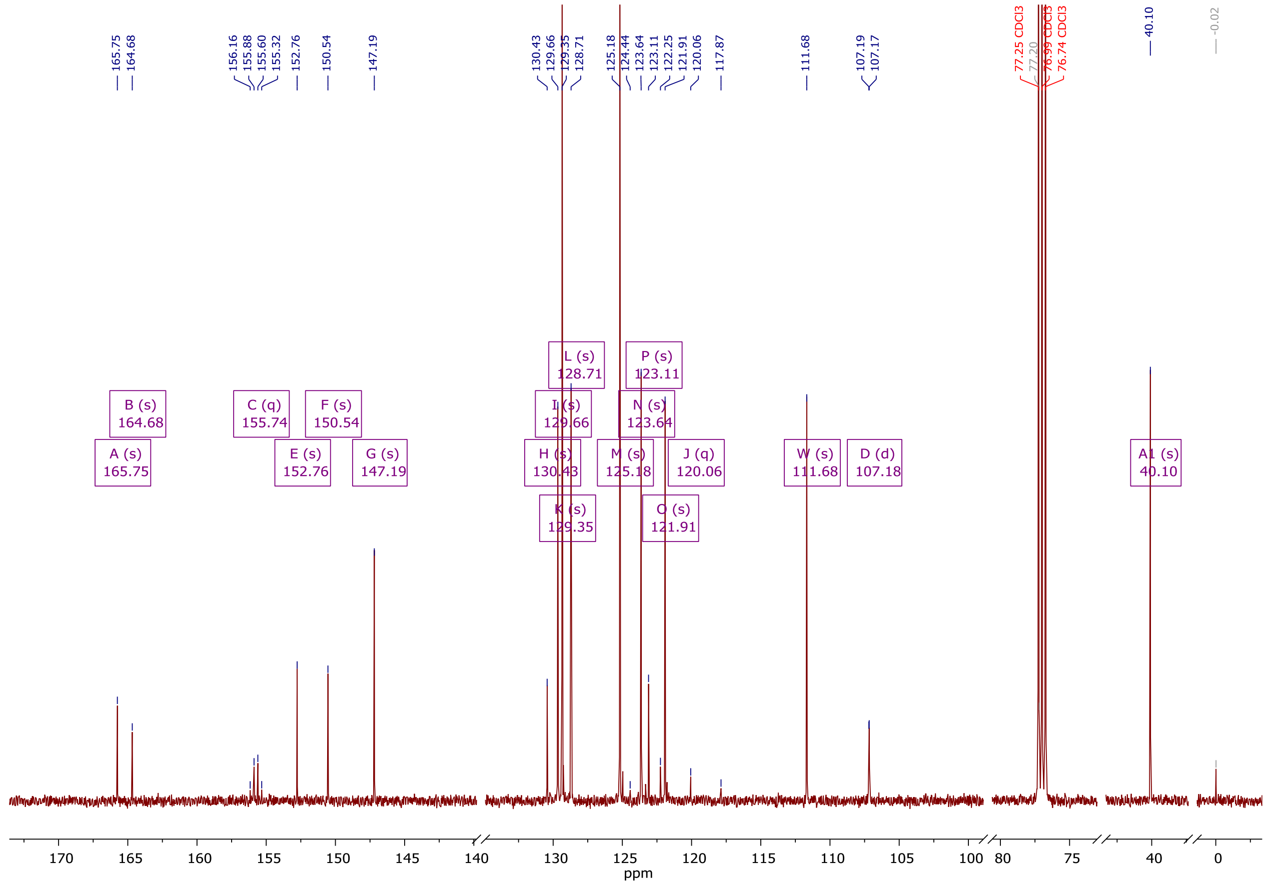

Figure S33. ^{13}C NMR (126 MHz, $CDCl_3$) spectrum of **10a**.

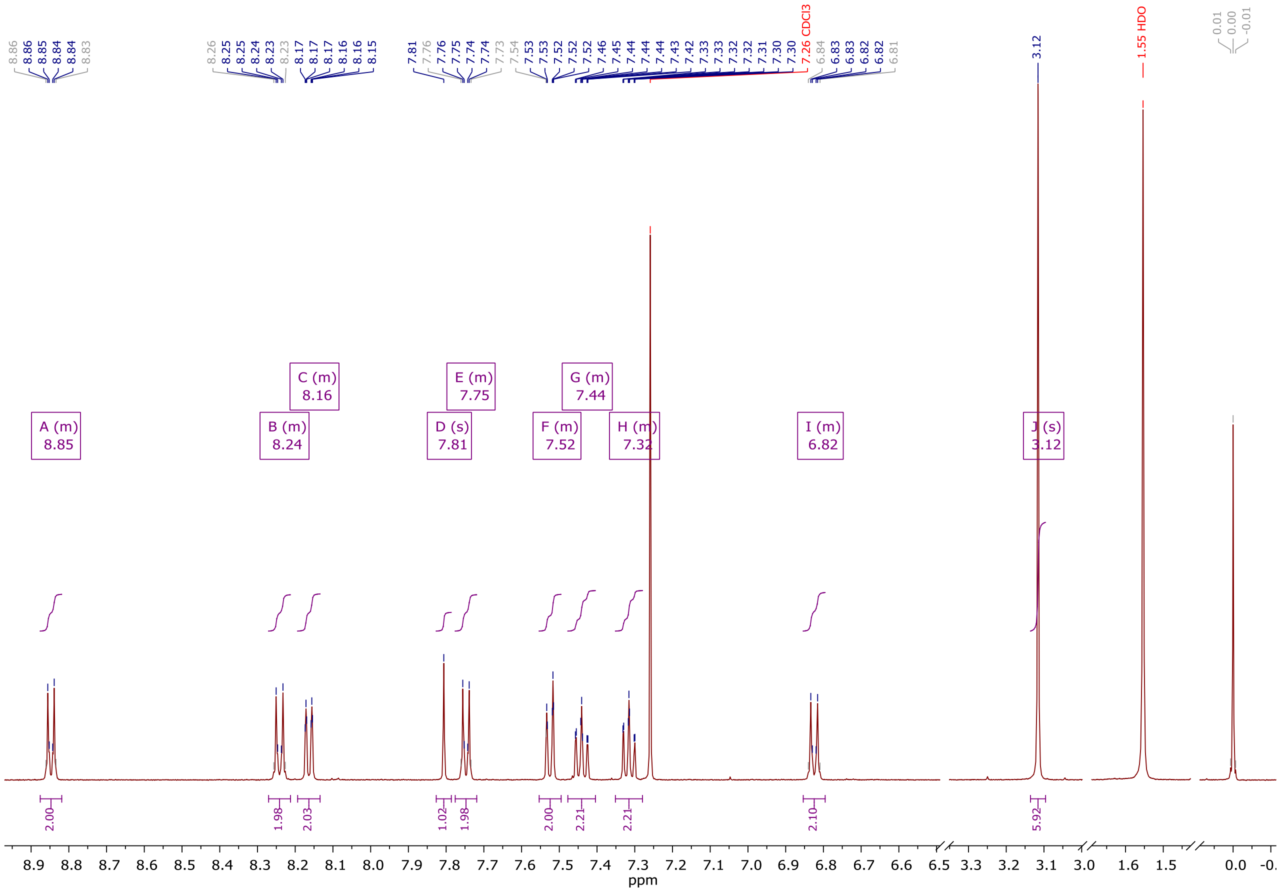

Figure S34. ^{1}H NMR (500 MHz, $CDCl_3$) spectrum of **10b**.

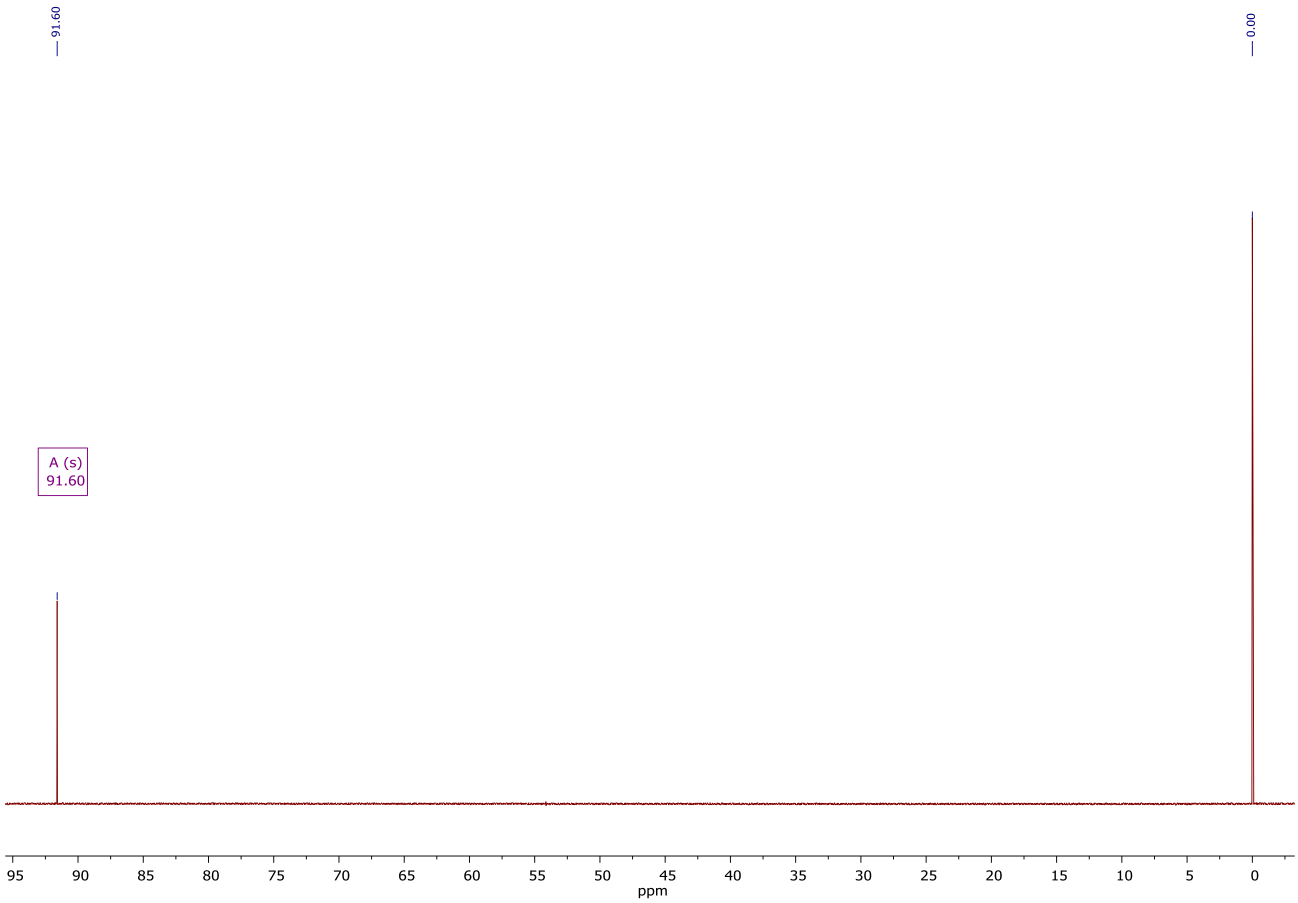

Figure S35. ^{19}F NMR (471 MHz, $CDCl_3$) spectrum of **10b**.

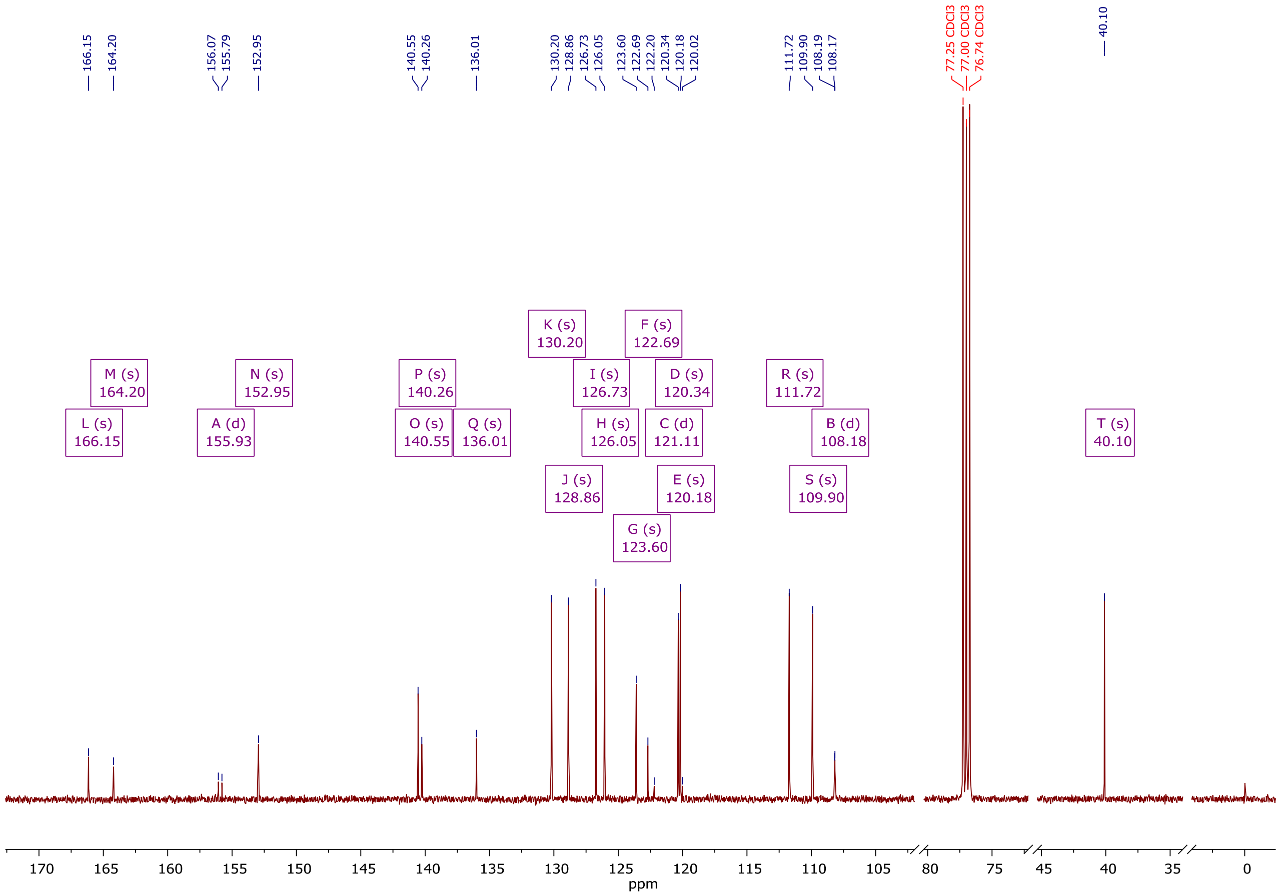

Figure S36. ^{13}C NMR (126 MHz, $CDCl_3$) spectrum of **10b**.

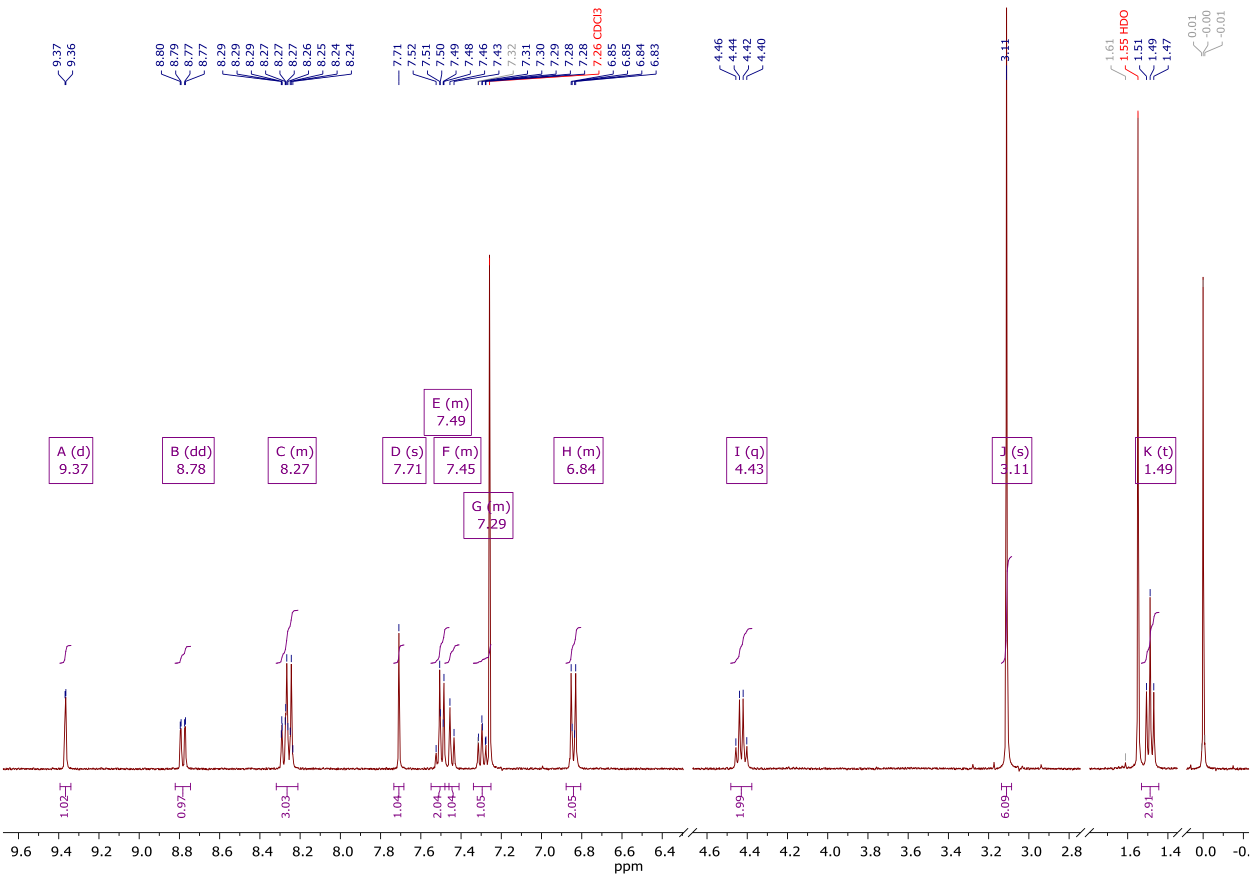

Figure S37. ^{1}H NMR (400 MHz, $CDCl_3$) spectrum of **10c**.

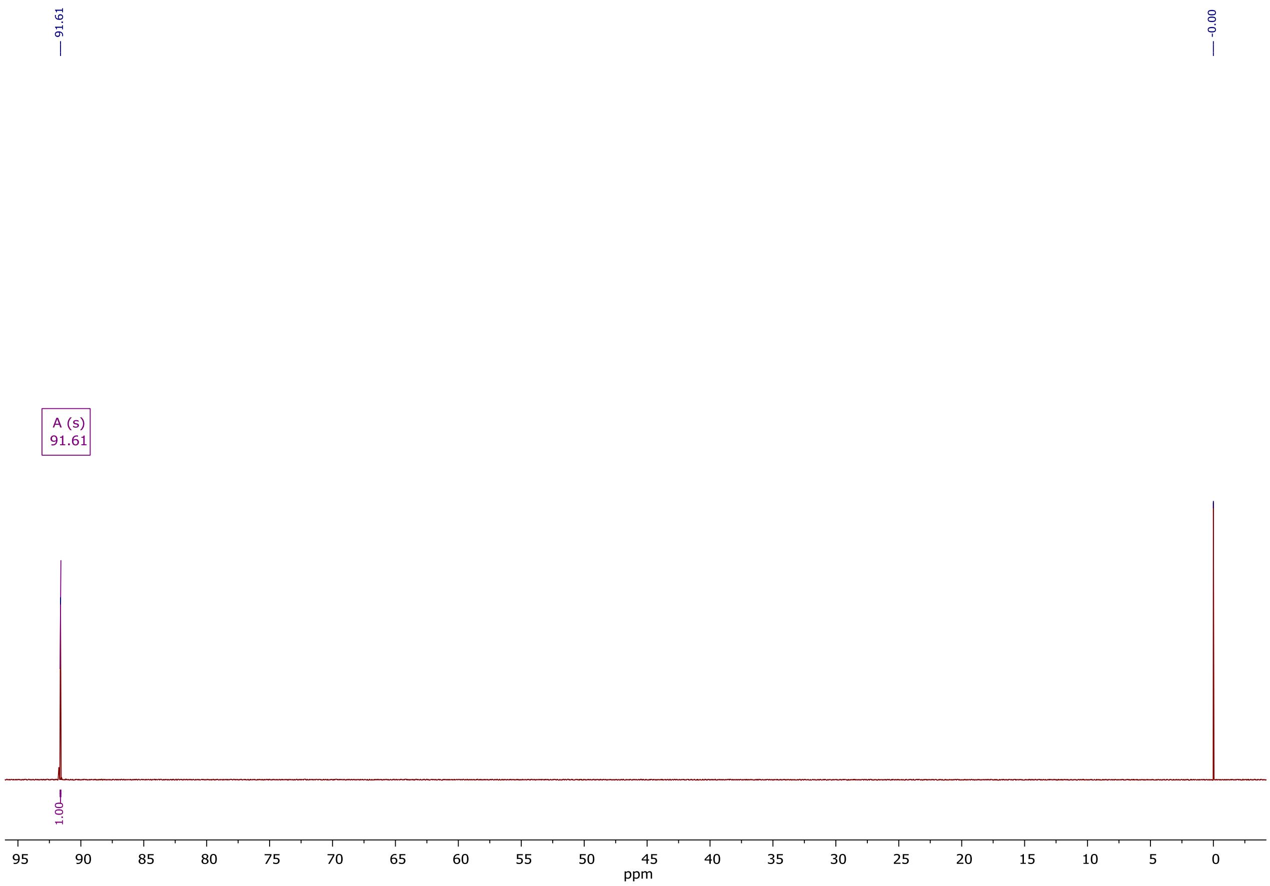

Figure S38. ^{19}F NMR (475 MHz, $CDCl_3$) spectrum of **10c**.

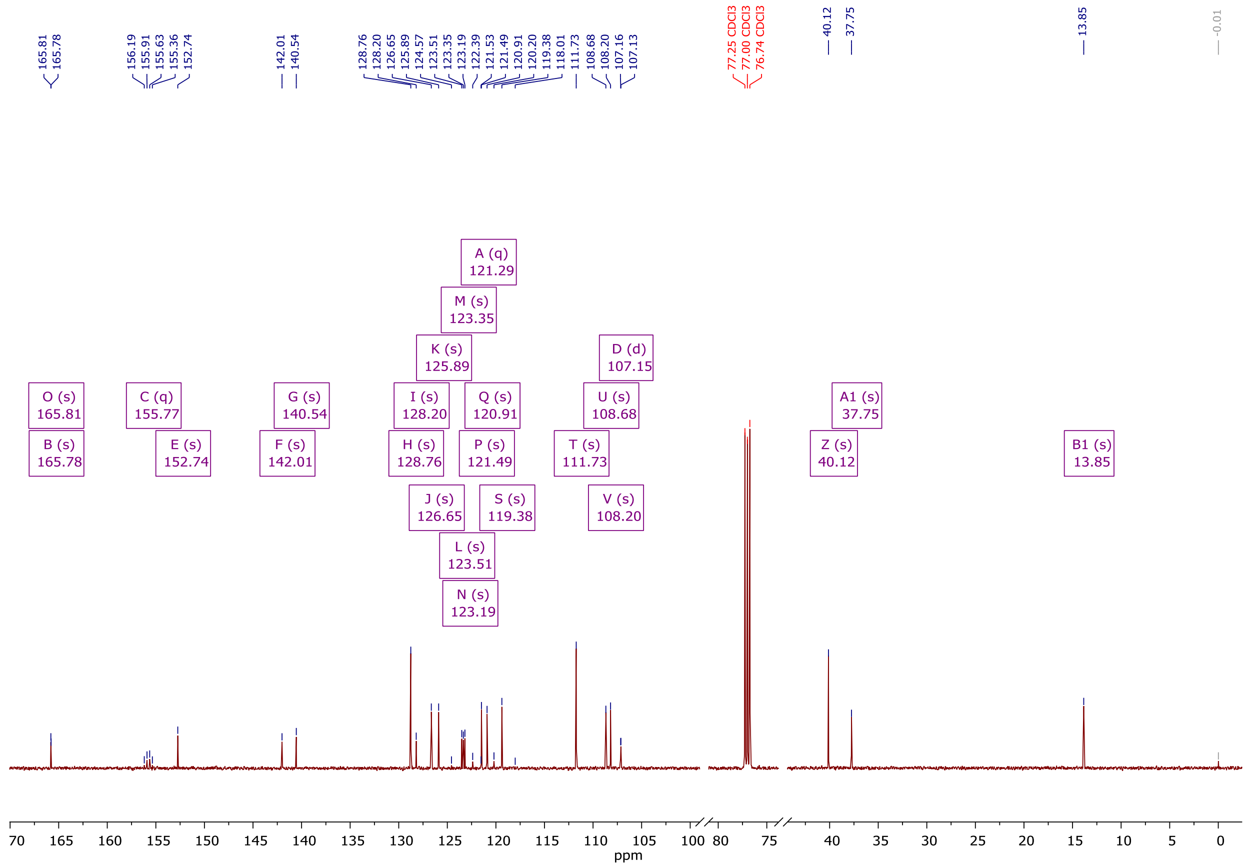

Figure S39. ^{13}C NMR (126 MHz, $CDCl_3$) spectrum of **10c**.

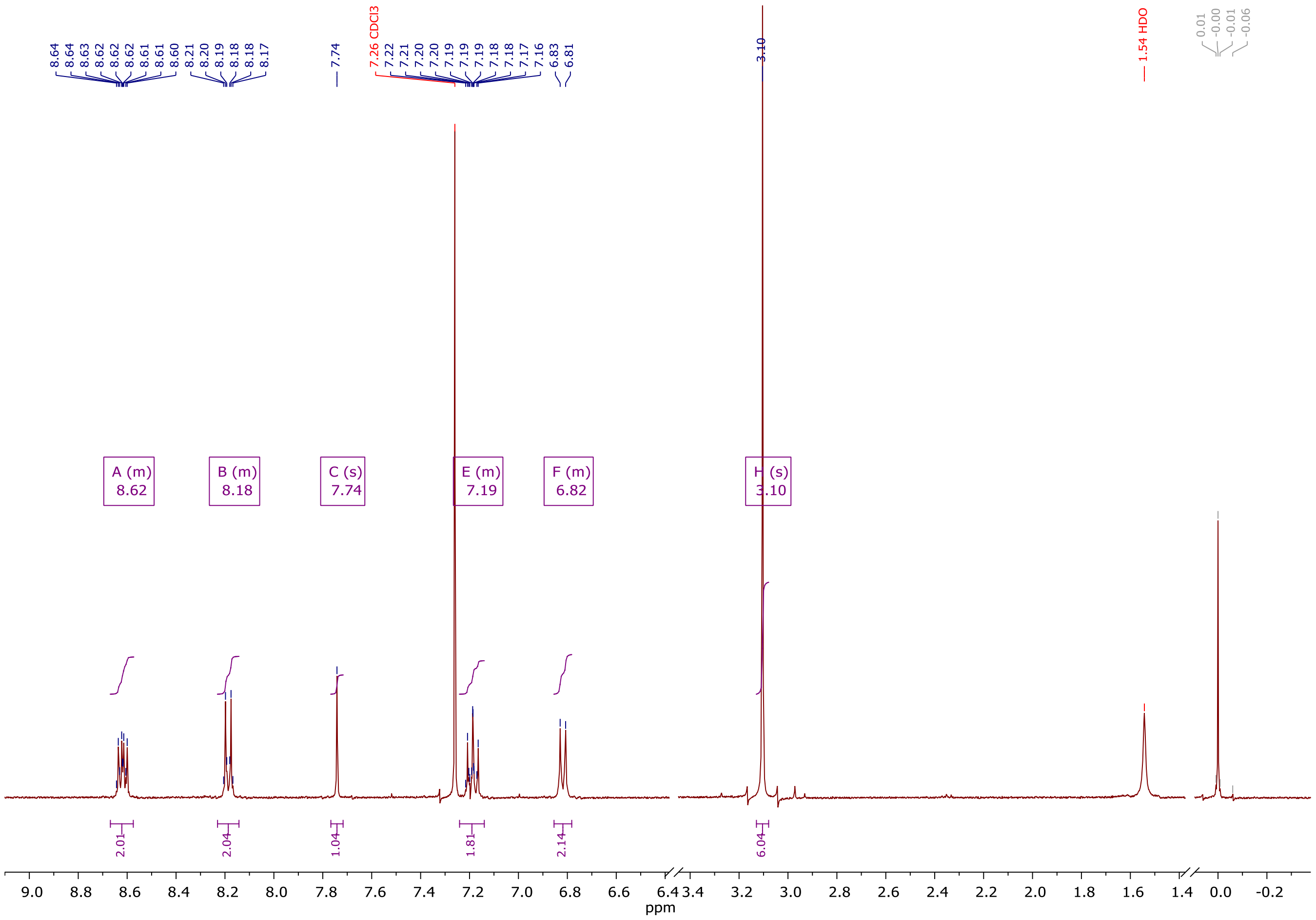

Figure S40. ^{1}H NMR (400 MHz, $CDCl_3$) spectrum of **10d**.

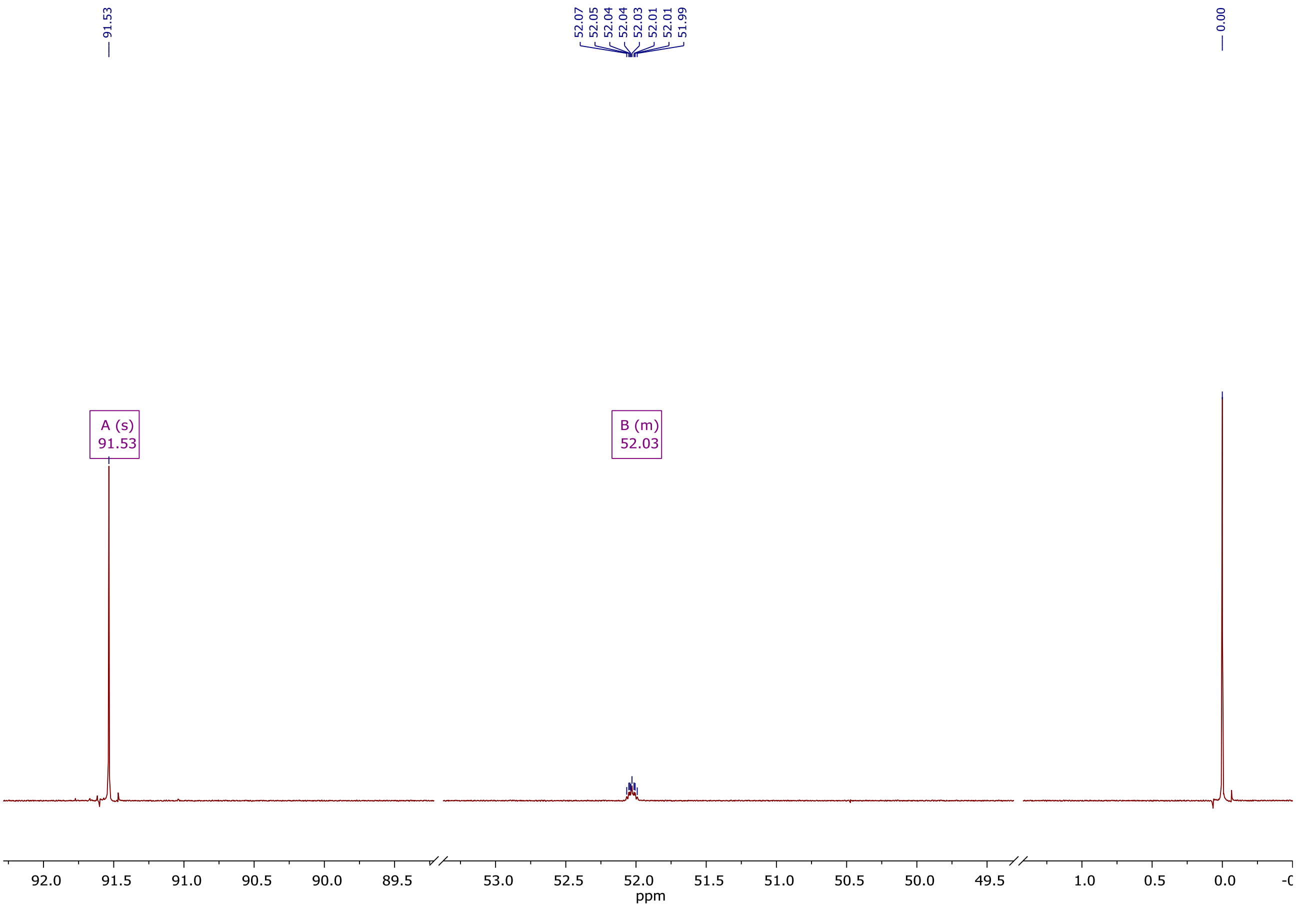

Figure S41. ^{19}F NMR (376 MHz, $CDCl_3$) spectrum of **10d**.

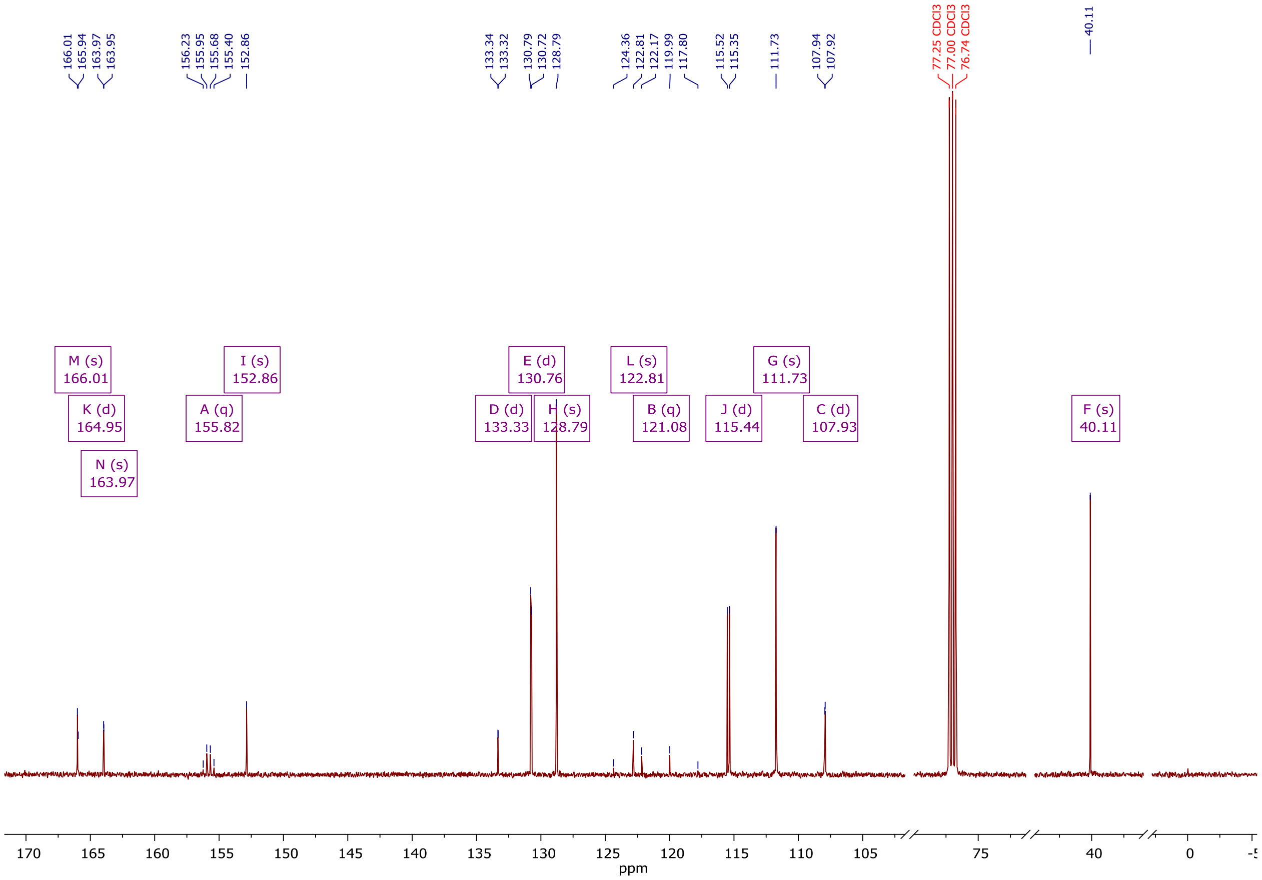

Figure S42. ^{13}C NMR (126 MHz, $CDCl_3$) spectrum of **10d**.

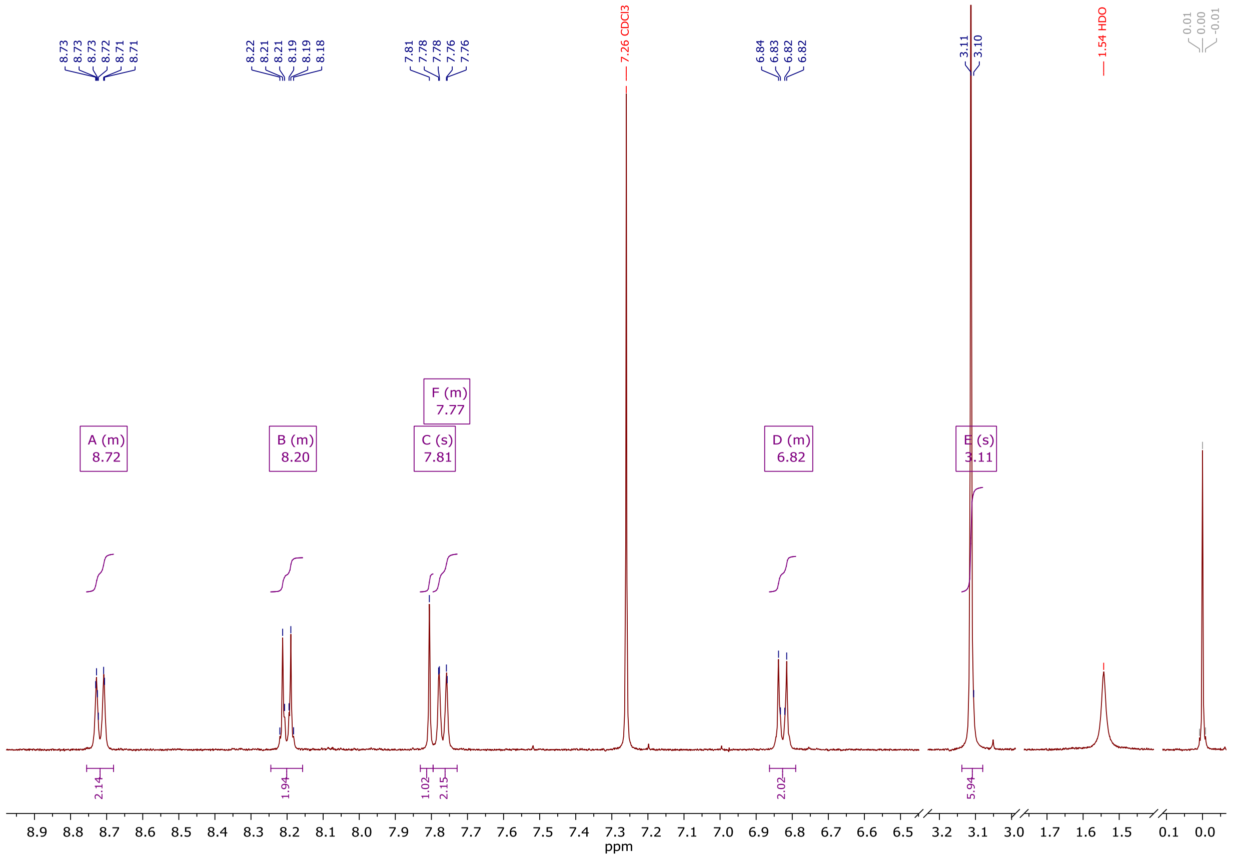

Figure S43. ^{1}H NMR (400 MHz, $CDCl_3$) spectrum of **10e**.

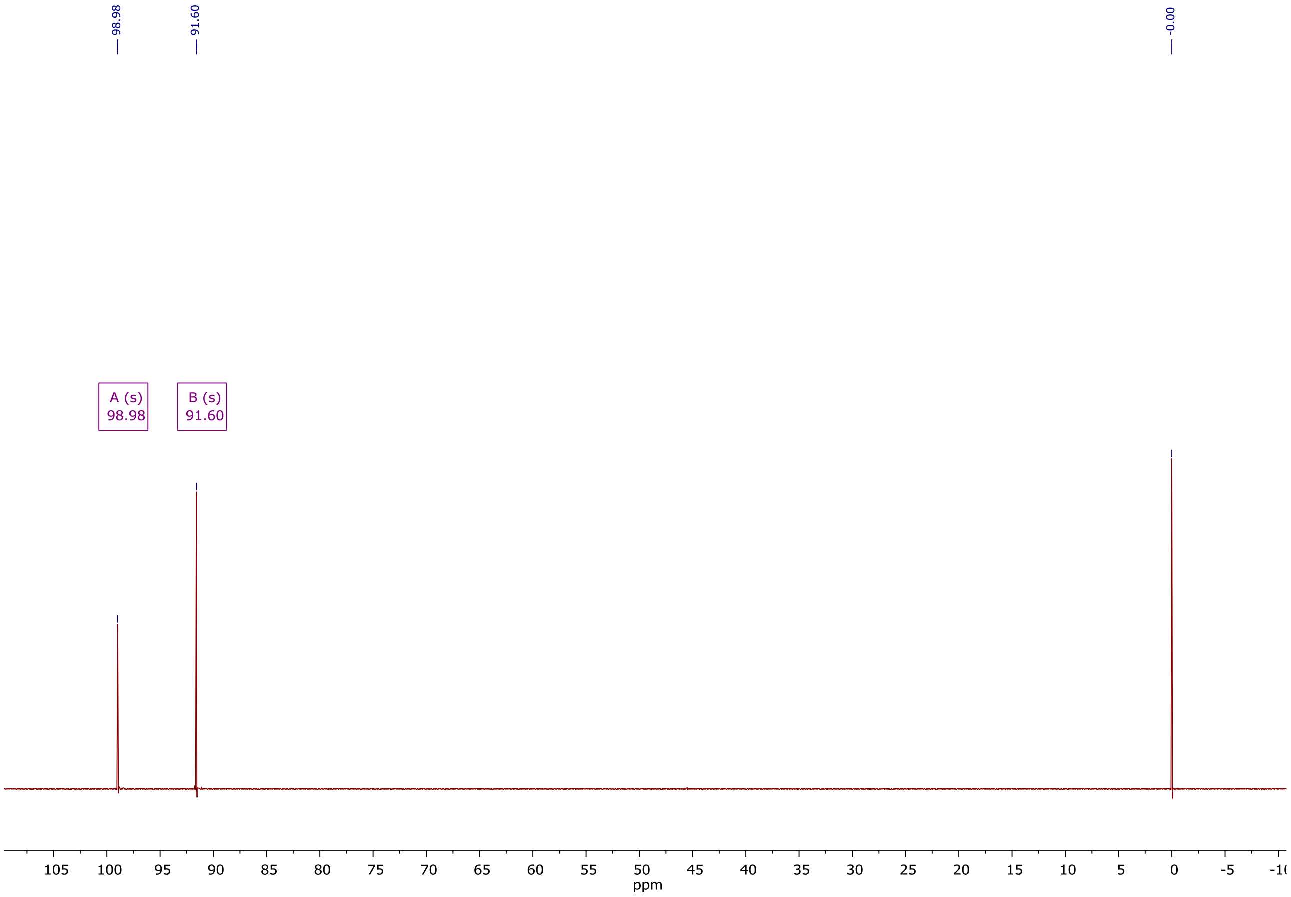

Figure S44. ^{19}F NMR (376 MHz, $CDCl_3$) spectrum of **10e**.

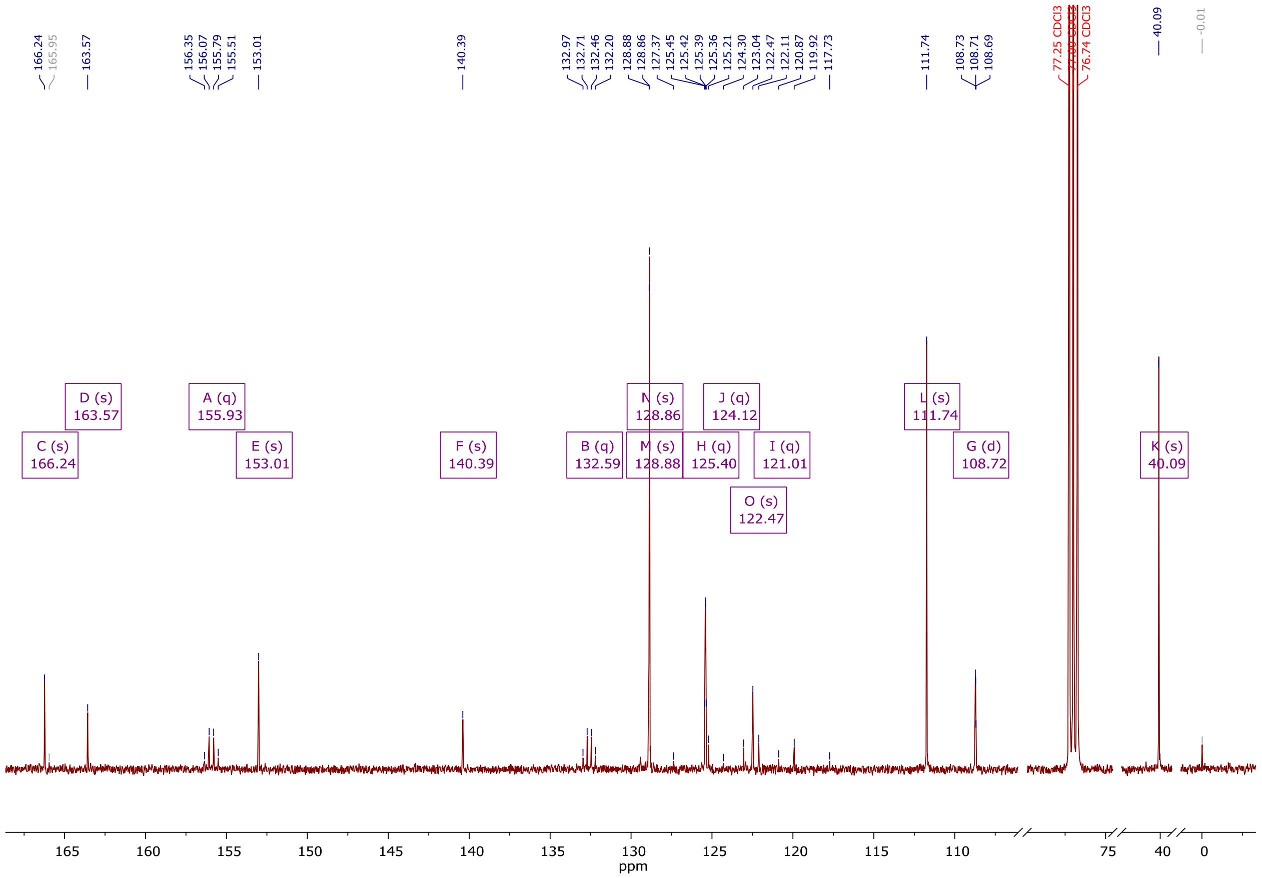

Figure S45. ^{13}C NMR (126 MHz, $CDCl_3$) spectrum of **10e**.

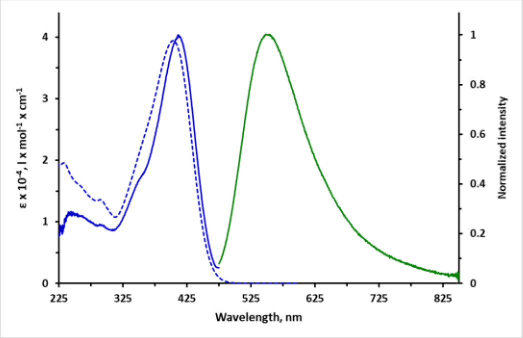

Figure S46. Absorption spectrum (--), excitation (–) and emission (–) spectra of **9a** in MeCN.

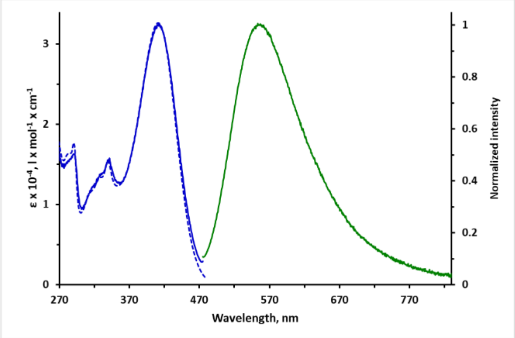

Figure S47. Absorption spectrum (--), excitation (–) and emission (–) spectra of **9b** in MeCN.

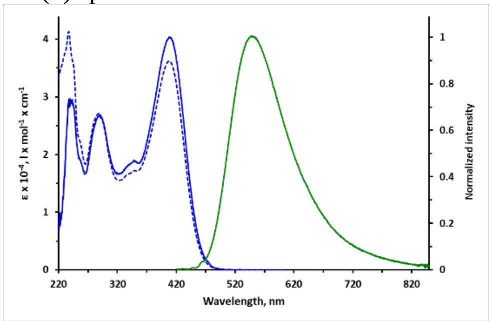

Figure S48. Absorption spectrum (--), excitation (–) and emission (–) spectra of **9c** in MeCN.

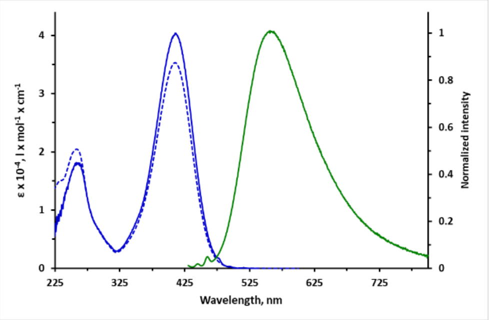

Figure S49. Absorption spectrum (--), excitation (–) and emission (–) spectra of **9d** in MeCN.

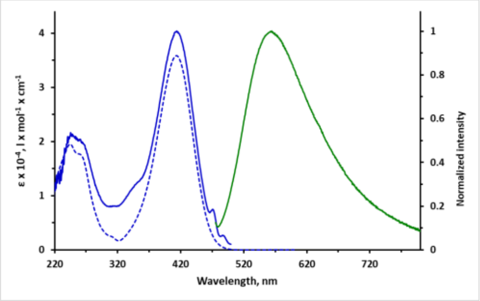

Figure S50. Absorption spectrum (--), excitation (–) and emission (–) spectra of **9e** in MeCN.

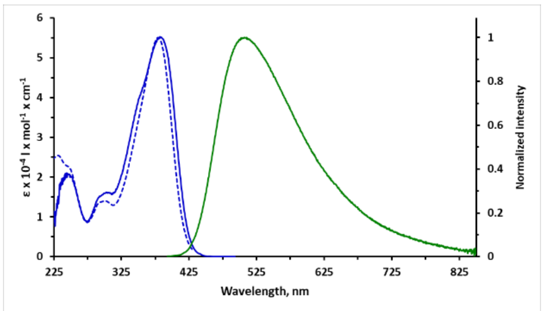

Figure S51. Absorption spectrum (--), excitation (–) and emission (–) spectra of **10a** in MeCN.

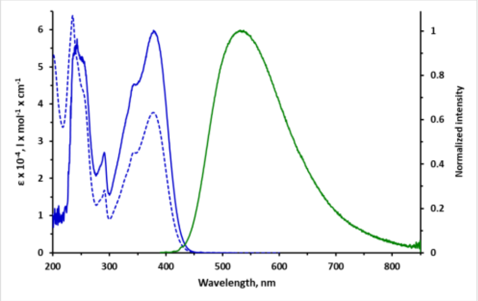

Figure S52. Absorption spectrum (--), excitation (–) and emission (–) spectra of **10b** in MeCN.

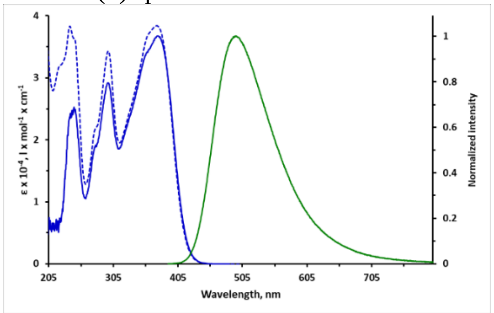

Figure S53. Absorption spectrum (--), excitation (–) and emission (–) spectra of **10c** in MeCN.

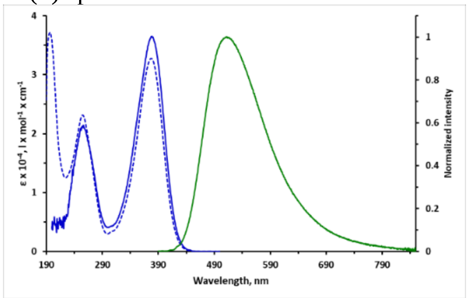

Figure S54. Absorption spectrum (--), excitation (–) and emission (–) spectra of **10d** in MeCN.

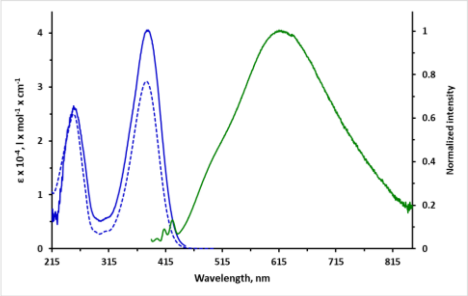

Figure S55. Absorption spectrum (--), excitation (–) and emission (–) spectra of **10e** in MeCN.

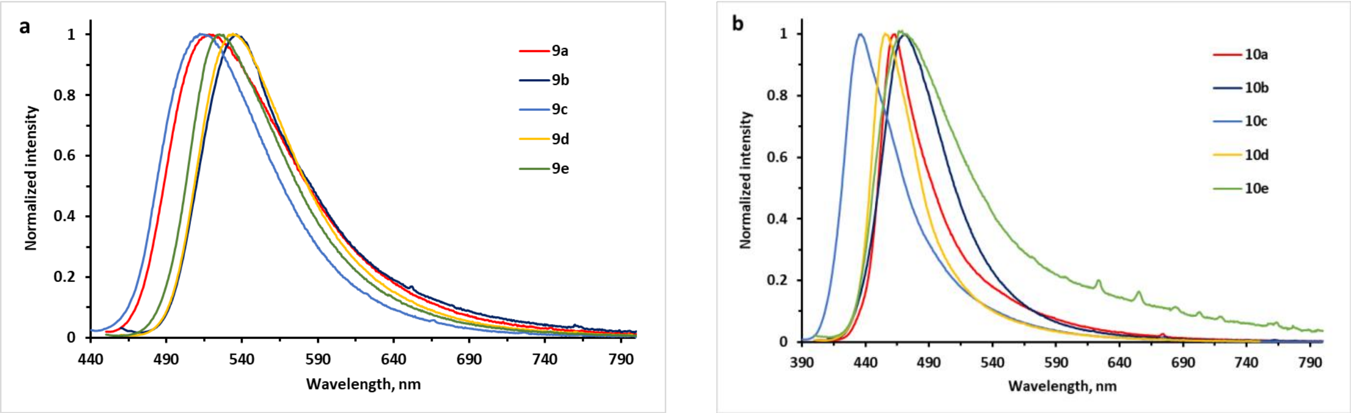

Figure S56. Emission spectra of **9a-e** in solid state.

Table S1. Detailed data of the fluorescence lifetime measurements, k_r – radiative transition rate constant ($k_r = \Phi_F/\tau_{avg}$) and k_{nr} – nonradiative transition rate constant ($k_{nr} = 1/\tau_{avg} - k_r$) values of **9a-e** and **10a-e** in MeCN: τ – lifetime, *f* - fractional contribution, τ avg – average lifetime, χ^2 - chi-squared distribution.

**Compound**	**Solution in MeCN**							k_r, s^{-1}	k_{nr}, s^{-1}
	**λ_{em} [nm]**	**τ_1, [ns]**	**f_1, %**	**τ_2, [ns]**	**f_2, %**	**τ_{avg}, [ns]**	**χ^2**		
**9a**	551	0.50	95.4	2.72	4.6	**0.60**	**1.295**	6.67×10^7	1.60×10^9
**9b**	556	0.37	96.9	3.96	3.1	**0.48**	**1.115**	9.09×10^7	1.05×10^9

**9c**	550	0.56	97.7	4.43	2.3	**0.64**	**1.309**	1.72×10^{8}	1.39×10^{9}
**9d**	556	0.35	93.9	3.30	6.1	**0.53**	**1.241**	9.43×10^{7}	1.79×10^{9}
**9e**	564	0.33	86.6	3.67	13.7	**0.78**	**1.050**	**-**	**-**
**10a**	506	0.47	41.3	2.28	58.7	**1.53**	**1.089**	3.27×10^{7}	6.21×10^{8}
**10b**	533	1.61	17.3	2.22	82.7	**2.12**	**1.044**	2.36×10^{7}	4.48×10^{8}
**10c**	495	1.52	8.70	2.46	91.3	**2.38**	**1.068**	1.39×10^{8}	2.82×10^{8}
**10d**	513	1.69	96.3	4.18	3.7	**1.78**	**1.103**	7.30×10^{7}	4.89×10^{8}
**10e**	620	0.60	94.1	3.36	5.9	**0.77**	**1.184**	1.30×10^{7}	1.29×10^{9}

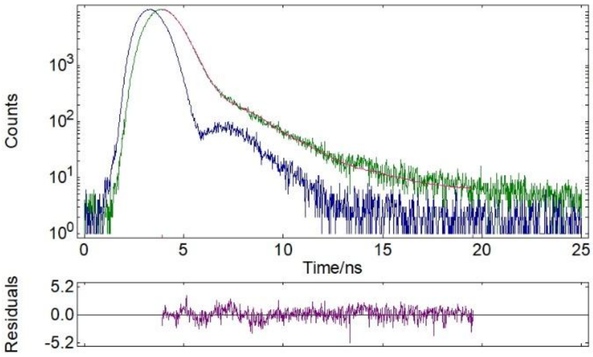

Figure S57. Time-resolved fluorescence lifetime decay profile of solution **9a** in MeCN (green), instrumental response function (IRF, blue). λ_{ex} = 300 nm, λ_{em} = 551 nm.

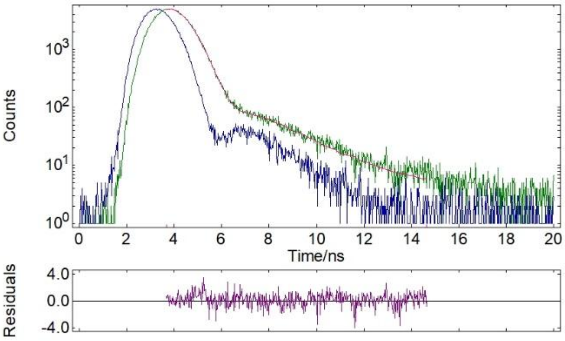

Figure S58. Time-resolved fluorescence lifetime decay profile of solution **9b** in MeCN (green), instrumental response function (IRF, blue). λ_{ex} = 300 nm, λ_{em} = 556 nm.

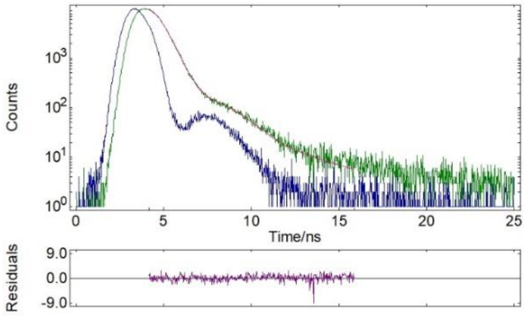

Figure S59. Time-resolved fluorescence lifetime decay profile of solution **9c** in MeCN (green), instrumental response function (IRF, blue). λ_{ex} = 300 nm, λ_{em} = 550 nm.

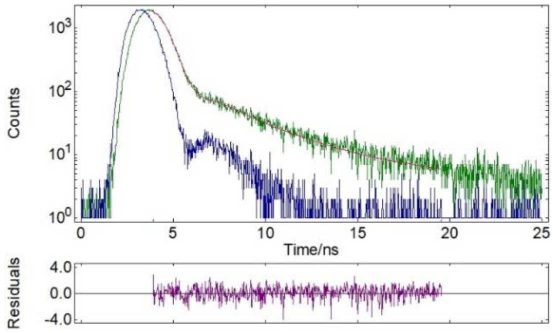

Figure S60. Time-resolved fluorescence lifetime decay profile of solution **9d** in MeCN (green), instrumental response function (IRF, blue). λ_{ex} = 300 nm, λ_{em} = 546 nm.

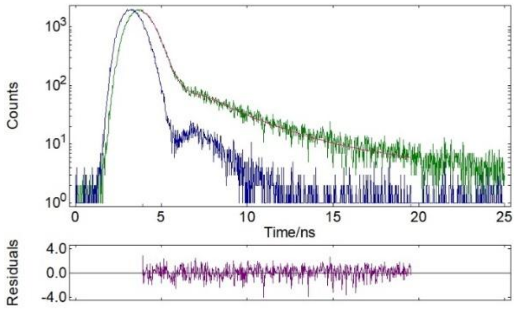

Figure S61. Time-resolved fluorescence lifetime decay profile of solution **9e** in MeCN (green), instrumental response function (IRF, blue). λ_{ex} = 300 nm, λ_{em} = 564 nm.

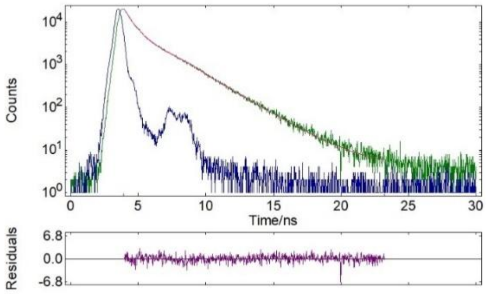

Figure S62. Time-resolved fluorescence lifetime decay profile of solution **10a** in MeCN (green), instrumental response function (IRF, blue). λ_{ex} = 375 nm, λ_{em} = 506 nm.

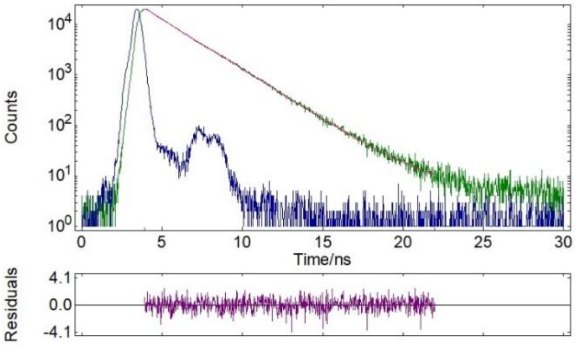

Figure S63. Time-resolved fluorescence lifetime decay profile of solution **10b** in MeCN (green), instrumental response function (IRF, blue). λ_{ex} = 375 nm, λ_{em} = 533 nm.

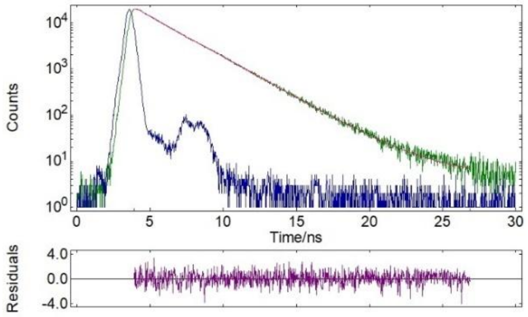

Figure S64. Time-resolved fluorescence lifetime decay profile of solution **10c** in MeCN (green), instrumental response function (IRF, blue). λ_{ex} = 375 nm, λ_{em} = 495 nm.

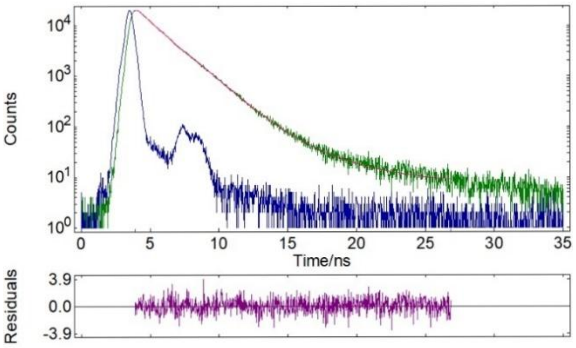

Figure S65. Time-resolved fluorescence lifetime decay profile of solution **10d** in MeCN (green), instrumental response function (IRF, blue). λ_{ex} = 375 nm, λ_{em} = 513 nm.

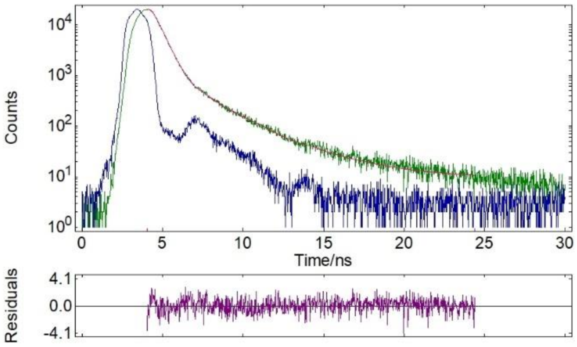

Figure S66. Time-resolved fluorescence lifetime decay profile of solution **10e** in MeCN (green), instrumental response function (IRF, blue). λ_{ex} = 375 nm, λ_{em} = 620 nm.

Table S2. Detailed data of the fluorescence lifetime measurements of solid **9a-e** and **10a-e**: τ – lifetime, *f* - fractional contribution, τ_{avg} – average lifetime, χ^2 - chi-squared distribution.

Compound	Solid									k_r, s^{-1}	k_{nr}, s^{-1}
	λ_{em} [nm]	τ_1, [ns]	f_1, %	τ_2, [ns]	f_2, %	τ_3, [ns]	f_3, %	τ_{avg}, [ns]	χ^2		
**9a**	518	1.17	34.6	4.89	44.8	17.05	20.6	**6.10**	**1.277**	1.31×10^7	1.51×10^8
**9b**	536	0.36	76.5	0.96	19.0	6.59	4.5	**0.75**	**1.179**	6.67×10^7	1.27×10^9
**9c**	515	3.62	49.3	9.89	39.6	24.54	11.1	**8.43**	**1.145**	9.49×10^6	1.09×10^8
**9d**	535	1.25	15.1	3.11	73.3	8.05	11.6	**3.40**	**1.166**	2.35×10^7	2.71×10^8
**9e**	525	1.31	23.2	3.90	59.4	11.58	17.4	**4.64**	**1.067**	2.59×10^7	1.90×10^8
**10a**	463	0.47	33.3	1.59	46.8	4.29	19.9	**1.76**	**1.074**	5.03×10^7	5.08×10^8
**10b**	470	0.95	22.5	2.15	68.0	4.71	9.5	**2.12**	**1.083**	2.83×10^7	4.43×10^8
**10c**	436	0.54	40.4	1.38	51.7	5.31	7.9	**1.36**	**1.024**	8.09×10^7	6.54×10^8
**10d**	456	1.09	52.7	2.72	35.7	10.77	11.6	**2.79**	**1.160**	2.87×10^7	3.30×10^8
**10e**	470	0.39	40.8	1.03	43.9	3.11	15.3	**1.08**	**1.065**	-	-

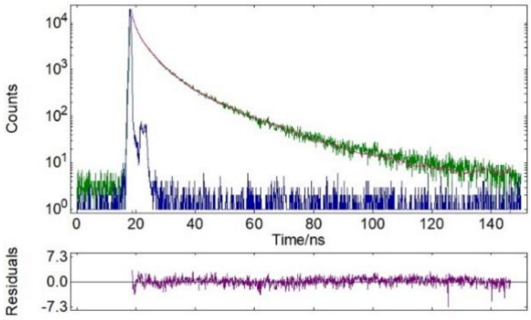

Figure S67. Time-resolved fluorescence lifetime decay profile of solid **9a** (green), instrumental response function (IRF, blue). λ_{ex} = 375 nm, λ_{em} = 518 nm.

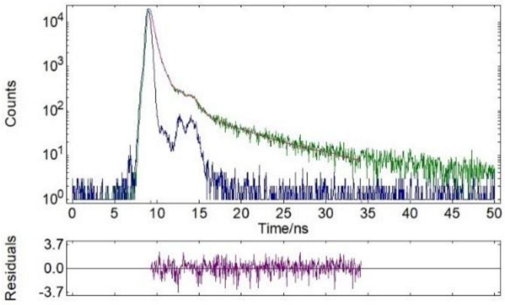

Figure S68. Time-resolved fluorescence lifetime decay profile of solid **9b** (green), instrumental response function (IRF, blue). λ_{ex} = 375 nm, λ_{em} = 536 nm.

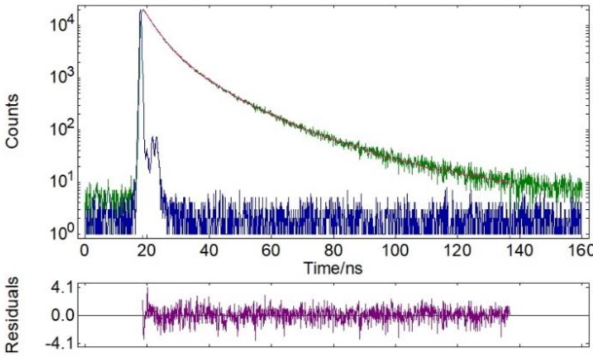

Figure S69. Time-resolved fluorescence lifetime decay profile of solid **9c** (green), instrumental response function (IRF, blue). λ_{ex} = 375 nm, λ_{em} = 515 nm.

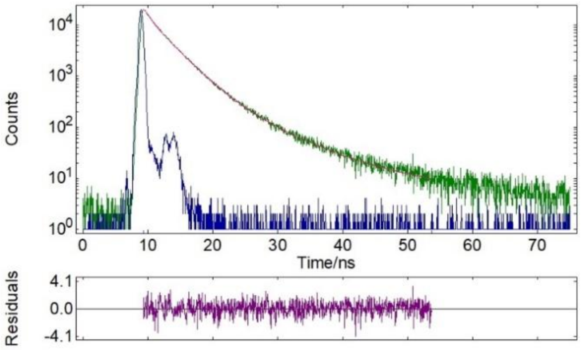

Figure S70. Time-resolved fluorescence lifetime decay profile of solid **9d** (green), instrumental response function (IRF, blue). λ_{ex} = 375 nm, λ_{em} = 535 nm.

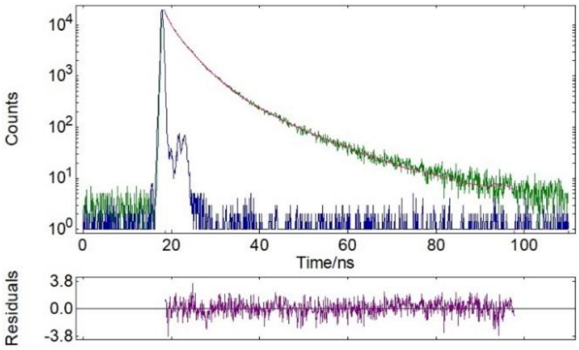

Figure S71. Time-resolved fluorescence lifetime decay profile of solid **9e** (green), instrumental response function (IRF, blue). λ_{ex} = 375 nm, λ_{em} = 525 nm.

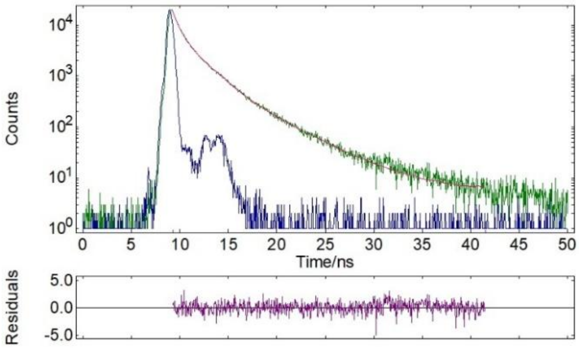

Figure S72. Time-resolved fluorescence lifetime decay profile of solid **10a** (green), instrumental response function (IRF, blue). λ_{ex} = 375 nm, λ_{em} = 463 nm.

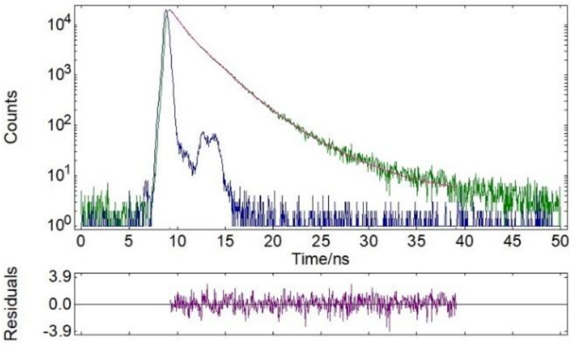

Figure S73. Time-resolved fluorescence lifetime decay profile of solid **10b** (green), instrumental response function (IRF, blue). λ_{ex} = 375 nm, λ_{em} = 470 nm.

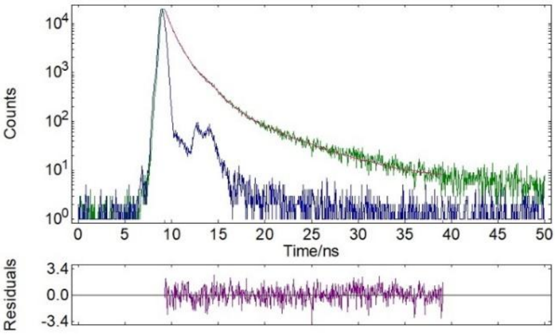

Figure S74. Time-resolved fluorescence lifetime decay profile of solid **10c** (green), instrumental response function (IRF, blue). λ_{ex} = 375 nm, λ_{em} = 436 nm.

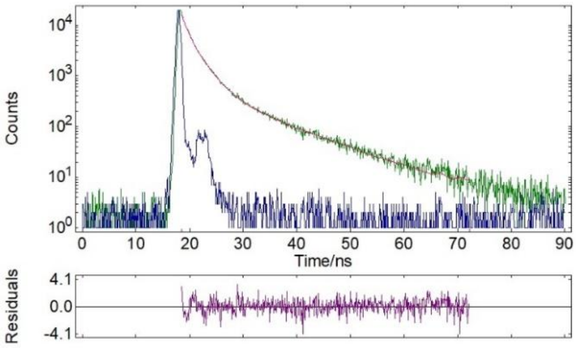

Figure S75. Time-resolved fluorescence lifetime decay profile of solid **10d** (green), instrumental response function (IRF, blue). λ_{ex} = 375 nm, λ_{em} = 456 nm.

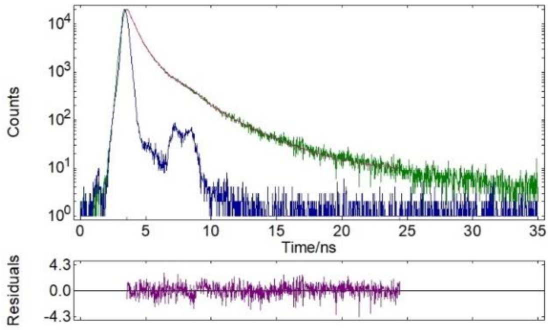

Figure S76. Time-resolved fluorescence lifetime decay profile of solid **10e** (green), instrumental response function (IRF, blue). λ_{ex} = 375 nm, λ_{em} = 470 nm.

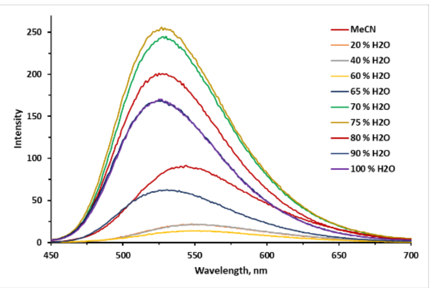

Figure S77. The fluorescence spectra of 10 μM **9a** in MeCN/H_2O mixtures with different water fractions (f_w).

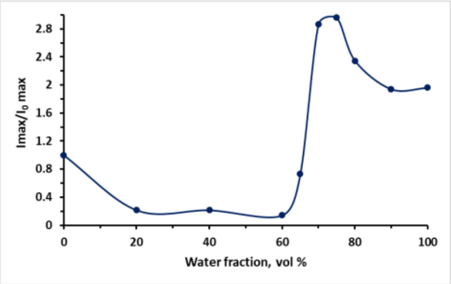

Figure S78. A plot of I/I_0 versus the composition of the MeCN/H_2O mixture for **9a** at λ = 530 nm.

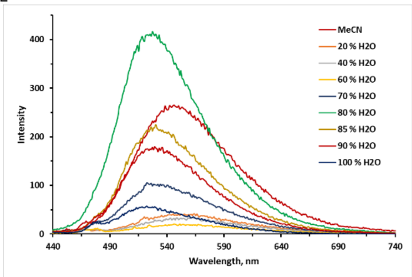

Figure S79. The fluorescence spectra of 10 μM **9b** in MeCN/H_2O mixtures with different water fractions (f_w).

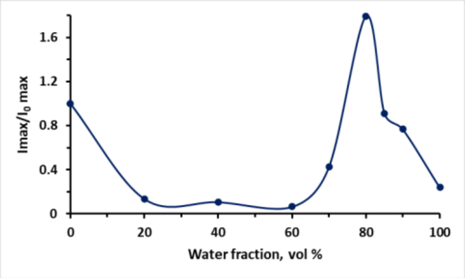

Figure S80. A plot of I/I_0 versus the composition of the MeCN/H_2O mixture for **9b** at λ = 527 nm.

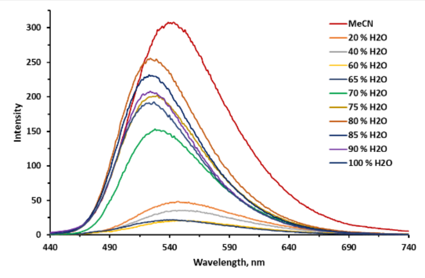

Figure S81. The fluorescence spectra of 10 μM **9c** in MeCN/H_2O mixtures with different water fractions (f_w).

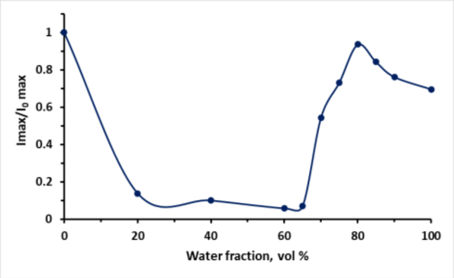

Figure S82. A plot of I/I_0 versus the composition of the MeCN/H_2O mixture for **9c** at λ = 524 nm.

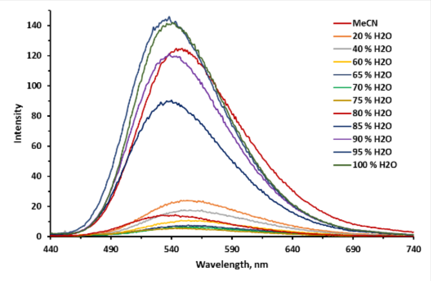

Figure S83. The fluorescence spectra of 10 μM **9d** in MeCN/H_2O mixtures with different water fractions (f_w).

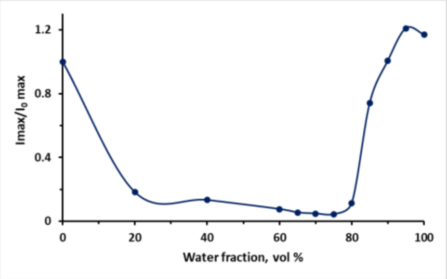

Figure S84. A plot of I/I_0 versus the composition of the MeCN/H_2O mixture for **9d** at λ = 538 nm.

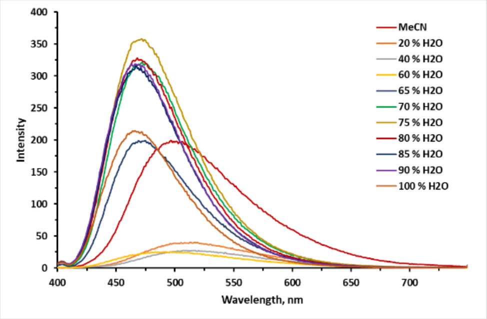

Figure S85. The fluorescence spectra of 10 μM **10a** in MeCN/H_2O mixtures with different water fractions (f_w).

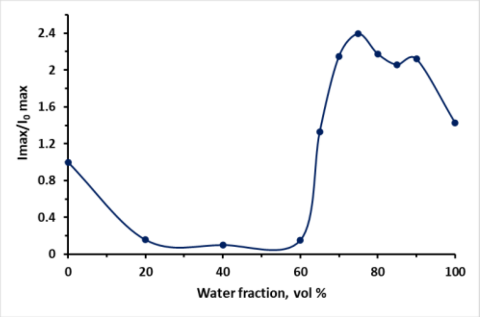

Figure S86. A plot of I/I_0 versus the composition of the MeCN/H_2O mixture for **10a** at λ = 472 nm.

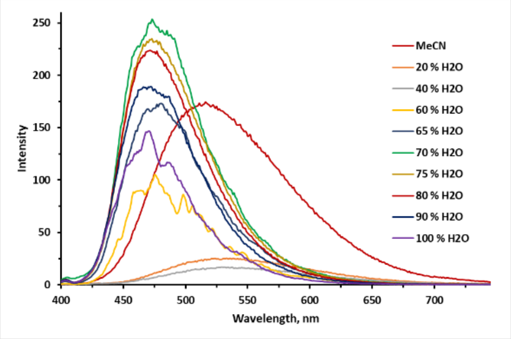

Figure S87. The fluorescence spectra of 10 μM **10b** in MeCN/H_2O mixtures with different water fractions (f_w).

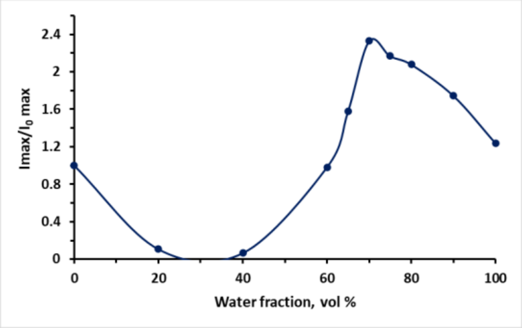

Figure S88. A plot of I/I_0 versus the composition of the MeCN/H_2O mixture for **10b** at λ = 475 nm.

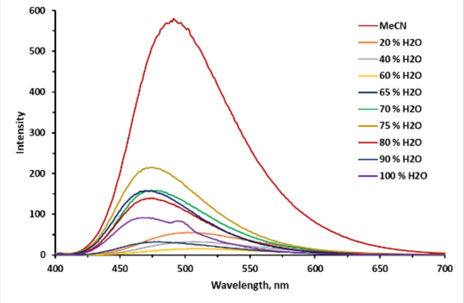

Figure S89. The fluorescence spectra of 10 μM **10c** in MeCN/H_2O mixtures with different water fractions (f_w).

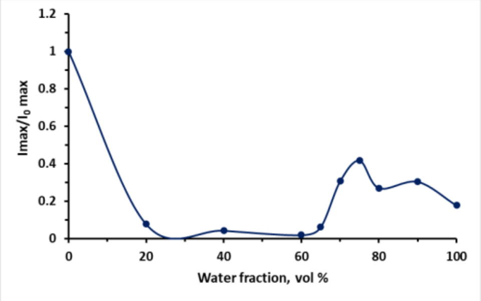

Figure S90. A plot of I/I_0 versus the composition of the MeCN/H_2O mixture for **10c** at λ = 475 nm.

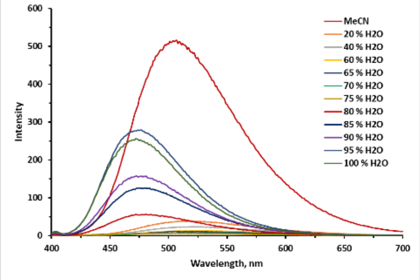

Figure S91. The fluorescence spectra of 10 μM **10d** in MeCN/H_2O mixtures with different water fractions (f_w).

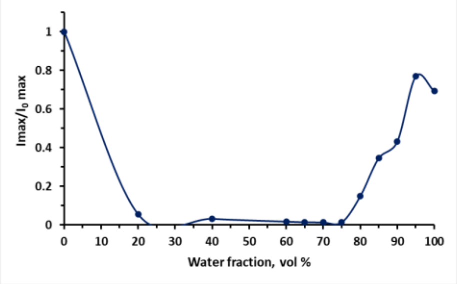

Figure S92. A plot of I/I_0 versus the composition of the MeCN/H_2O mixture for **10d** at λ = 475 nm.

Biological investigation

Cell cultivation

The *Vera*, *HaCaT*, *HEK-293t*, and *CaCo2* cell cultures were obtained from the cell culture bank of the Institute of Cytology RAS (Russia, St. Petersburg). The cell culture was maintained in culture flasks (Eppendorf, USA), in DMEM medium (Sigma Aldrich, USA) supplemented with 10% fetal bovine serum (Biolot, Russia) and 0.5% gentamicin (Biolot, Russia) in a 37 ºC incubator with a humid atmosphere of 5% CO_2.

The staining protocol

Solutions of the test substances were prepared in DMSO with concentrations of 1×10^{-3} M and diluted with complete nutrient medium to a concentration of 1×10^{-5} M. Solutions were prepared immediately before adding substances to cells. All manipulations with solutions of substances and, subsequently, with cells were carried out in a Bioinnelix laminar flow hood (BioinnLabs, Russia) under red illumination (625 nm) to minimize the effect of light on substances.

Cells were seeded into glass-bottom dishes for confocal microscopy (JetBiofil, China) at a concentration of 1×10^{4} cells/ml and incubated for 24 hours, after which a solution of the test substances in a complete nutrient medium was added to them. The cells were kept with the substance for 30 minutes in an incubator, after which they were washed three times with a nutrient medium to remove the dye that did not enter the cell and were examined using a confocal microscope. When moving the petri dishes into the confocal microscope room, the dishes were wrapped in aluminum foil to protect the cells as much as possible from light exposure.

For additional staining, the dye for lipid droplets BDP 650/665 (Lumiprob, Russia), for mitochondria MitoTracker Red (Thermo Scientific, USA) and lysosomes LumiTracker Lyso Red (Lumiprob, Russia) were used. The dyes were used according to the manufacturer's instructions and added in the required concentration (10, 20, and 50 nM, respectively) to the nutrient medium together with the test substance.

A laser scanning confocal microscope LSM 710 (Carl Zeiss, Germany) was used for the study. Cells were examined using a 40x immersion objective. The focus was first adjusted using a 633 nm laser to minimally affect the cells, then lambda (full spectrum) images were taken from other lasers.

Fluorescence spectra were extracted from lambda images using the PyLSM program (https://github.com/arteys/PyLSM) as well as ImageJ. Full color images were also reconstructed using the PyLSM program. It should be noted that band pass filters are installed in the microscope, which lead to dips in the emission graphs obtained using a confocal microscope.

Cell cytotoxicity and phototoxicity

Solutions of tested compounds in DMSO with a concentration of 1×10^{-2} M were diluted with a complete nutrient medium to achieve final concentrations of 1×10^{-4} M, 1×10^{-6} M, and 1×10^{-8} M. These solutions were prepared just before introducing the substances to the cells. All procedures involving the substance solutions and the subsequent handling of the cells were conducted in a Bioinnelix laminar flow hood (BioinnLabs, Russia) under red illumination (625 nm) to minimize the impact of light on the substances.

The study was conducted on *Vero* cells (green monkey kidney epithelial cells) obtained from the Russian cell culture collection of the Institute of Cytology of the Russian Academy of Sciences. The cells were maintained in a DMEM nutrient medium (BioinnLabs, Russia) with the addition of 10% fetal bovine serum (Biolot, Russia) in an incubator at a temperature of 37C and an atmosphere containing 5% CO_2. Before the experiments, the cells were tested for the absence of mycoplasma.

For the study, cells were seeded into a 96-well plate (JetBiofil, China) and grown until they reached 70% monolayer. Afterward, the medium was carefully collected using a multichannel dispenser and replaced with a prepared medium that included the test compound.

After 48-hour incubation with the substances, a resazurin solution (Thermo Scientific, USA) at a concentration of 0.42 mM was added to the wells of the plate in an amount of 20 μl per well. The medium was removed from the corner wells and DMSO was added for 5 minutes to kill the cells and use these wells as blanks. Then DMSO was removed, the wells were washed with phosphate buffer, and fresh medium was poured into them in a volume corresponding to the other wells. Chemidoc MP (Biorad, USA) was used to record the fluorescence intensity in three spectral ranges: Green Epi with a 605/50 green filter to measure the intensity of resazurin, Red Epi with a 695/55 filter for resazurin and Blue Epi with a 530/28 filter to obtain an image of the plate itself. The obtained images were processed using the CellProfiler (4.2.5) program[1] to segment the plate cells and measure the fluorescence intensity of each of them.

The investigation of the photodynamic effect was conducted using a 96-well plate. A specially designed box containing two Camelion Sp E27 26W light bulbs, which emit a maximum wavelength of 380 nm, served as the UV radiation source. Cytotoxicity was assessed after 72 hours using the resazurin assay.

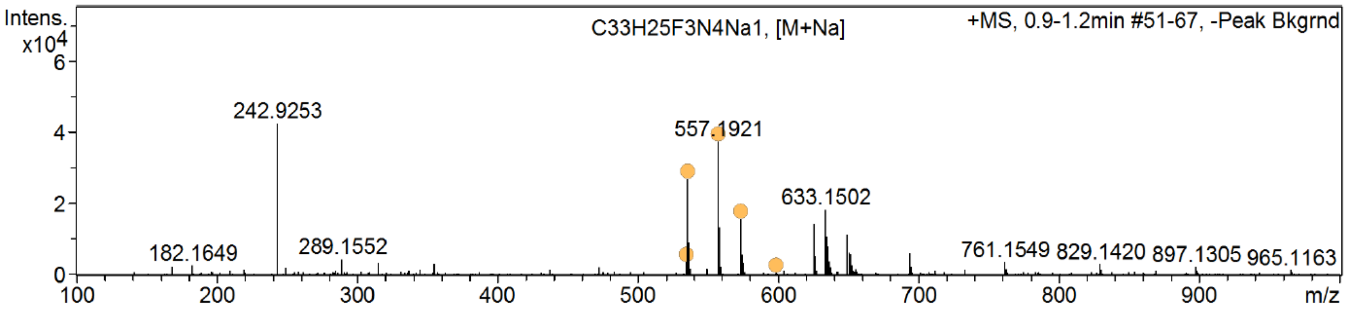

Meas. m/z	#	Ion Formula	m/z	err [ppm]	mSigma	# mSigma	Score	rdb	e^- Conf	N-Rule
534.2023	1	C33H25F3N4	534.2026	0.6	553.0	1	100.00	22.0	odd	ok
	1	C33H25F3N4	534.2026	0.6	553.0	1	100.00	22.0	odd	ok
535.2096	1	C33H26F3N4	535.2104	1.6	16.3	8	70.02	21.5	even	ok
	1	C33H26F3N4	535.2104	1.6	16.3	1	100.00	21.5	even	ok
	1	C33H26F3N4	535.2104	1.6	16.3	1	100.00	21.5	even	ok

Meas. m/z	#	Ion Formula	m/z	err [ppm]	mSigma	# mSigma	Score	rdb	e^- Conf	N-Rule
	1	C33H26F3N4	535.2104	1.6	16.3	1	100.00	21.5	even	ok
557.1921	1	C33H25F3N4Na	557.1924	0.5	8.8	1	100.00	21.5	even	ok
573.1661	1	C33H25F3KN4	573.1663	0.4	35.5	1	100.00	21.5	even	ok
598.2172	1	C35H28F3N5Na	598.2189	2.8	170.5	1	100.00	22.5	even	ok
1091.3948	1	C66H50F6N8Na	1091.3955	0.6	8.2	1	100.00	42.5	even	ok

Figure S93. HRMS spectrum of **9a**.

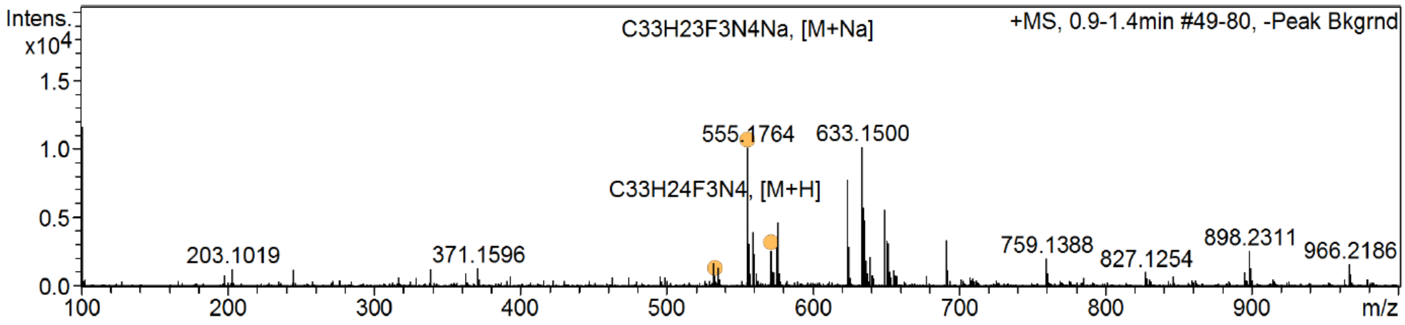

Meas. m/z	#	Ion Formula	m/z	err [ppm]	mSigma	# mSigma	Score	rdb	e^- Conf	N-Rule
533.1947	1	C33H24F3N4	533.1948	0.1	553.6	1	100.00	22.5	even	ok
555.1764	1	C32H27F3NaO4	555.1754	-1.9	20.7	1	85.84	17.5	even	ok
	2	C33H23F3N4Na	555.1767	0.5	32.3	2	100.00	22.5	even	ok
	1	C29H31F3KO5	555.1755	-1.7	37.6	1	82.86	12.5	even	ok
	2	C30H27F3KN4O	555.1769	0.7	40.5	2	100.00	17.5	even	ok
	1	C29H31F3KO5	555.1755	-1.7	37.6	1	100.00	12.5	even	ok
	1	C30H27F3KN4O	555.1769	0.7	40.5	1	100.00	17.5	even	ok
571.1503	1	C32H27F3KO4	571.1493	-1.8	134.3	1	100.00	17.5	even	ok
	1	C33H23F3KN4	571.1506	0.5	131.3	1	100.00	22.5	even	ok
1087.3632	1	C64H54F6NaO8	1087.3615	-1.5	20.5	1	100.00	34.5	even	ok
	1	C66H46F6N8Na	1087.3642	0.9	2.5	1	100.00	44.5	even	ok

Figure S94. HRMS spectrum of **9b**.

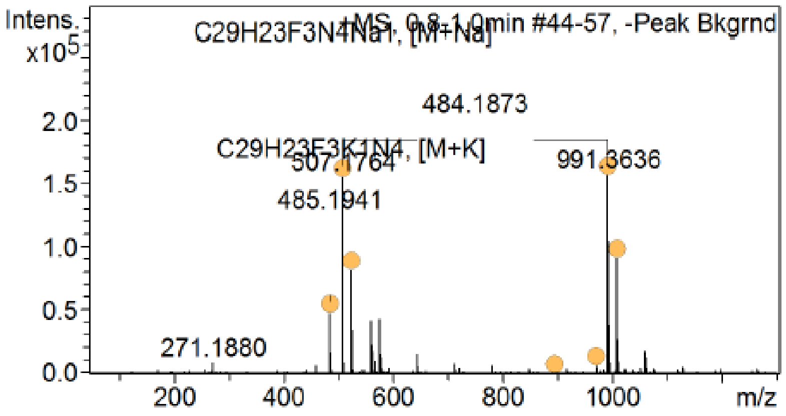

#	m/z	Res.	S/N	I	I %	FWHM
1	485.1941	20287	3537758.5	47882	30.6	0.0239
2	507.1764	22448	11419012.0	154551	98.8	0.0226
3	508.1794	20284	3660532.5	49544	31.7	0.0251
4	523.1502	21107	6025956.0	81559	52.2	0.0248
5	559.1312	21140	3109688.3	42088	26.9	0.0264
6	575.1635	20439	3198477.8	43290	27.7	0.0281
7	991.3636	28793	11554847.0	156390	100.0	0.0344
8	992.3666	26076	7710446.0	104357	66.7	0.0381
9	1007.3384	24124	6719788.5	90949	58.2	0.0418
10	1008.3411	22937	4468034.0	60473	38.7	0.0440

Figure S95. HRMS spectrum of **9c**.

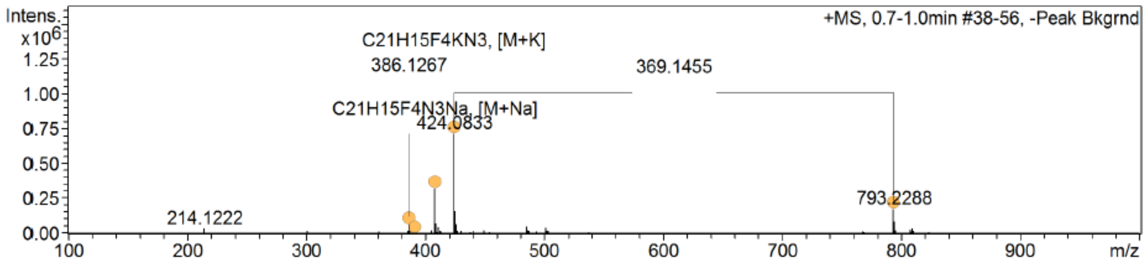

Meas. m/z	#	Ion Formula	m/z	err [ppm]	mSigma	# mSigma	Score	rdb	e$^-$ Conf	N-Rule
386.1267	1	C19H13FN9	386.1272	1.5	4.0	1	100.00	17.5	even	ok
	2	C21H16F4N3	386.1275	2.1	4.1	2	88.30	13.5	even	ok
	3	C18H17FN5O4	386.1259	-2.0	10.0	3	80.62	12.5	even	ok
	4	C15H18F2N5O5	386.1271	1.0	28.5	4	66.60	8.5	even	ok
	1	C19H13FN9	386.1272	1.5	4.0	1	100.00	17.5	even	ok

Meas. m/z	#	Ion Formula	m/z	err [ppm]	mSigma	# mSigma	Score	rdb	e$^-$ Conf	N-Rule
	2	C21H16F4N3	386.1275	2.1	4.1	2	88.30	13.5	even	ok
	3	C18H17FN5O4	386.1259	-2.0	10.0	3	80.62	12.5	even	ok
	4	C15H18F2N5O5	386.1271	1.0	28.5	4	66.60	8.5	even	ok
	1	C24H17N3NaO	386.1264	-0.8	23.0	1	100.00	17.5	even	ok
	1	C21H21KN3O2	386.1265	-0.4	38.3	1	100.00	12.5	even	ok
	1	C21H21KN3O2	386.1265	-0.4	38.3	1	100.00	12.5	even	ok
	1	C21H21KN3O2	386.1265	-0.4	38.3	1	100.00	12.5	even	ok
	1	C21H21KN3O2	386.1265	-0.4	38.3	1	100.00	12.5	even	ok
	1	C21H21KN3O2	386.1265	-0.4	38.3	1	100.00	12.5	even	ok
	1	C21H21KN3O2	386.1265	-0.4	38.3	1	100.00	12.5	even	ok
391.0829	1	C19H9FN8Na	391.0826	-0.7	282.6	1	100.00	18.5	even	ok
	1	C21H12F4N2Na	391.0829	-0.1	282.6	1	100.00	14.5	even	ok
	1	C15H14F2N4NaO5	391.0824	-1.2	269.1	1	100.00	9.5	even	ok
408.1092	1	C19H12FN9Na	408.1092	0.0	5.4	1	100.00	17.5	even	ok
	1	C21H15F4N3Na	408.1094	0.6	5.4	1	100.00	13.5	even	ok
	1	C15H17F2N5NaO5	408.1090	-0.5	26.7	1	100.00	8.5	even	ok
424.0833	1	C19H12FKN9	424.0831	-0.5	13.0	1	100.00	17.5	even	ok
	1	C21H15F4KN3	424.0834	0.1	13.0	1	100.00	13.5	even	ok
	1	C15H17F2KN5O5	424.0829	-0.9	23.0	1	100.00	8.5	even	ok
793.2288	1	C38H24F2N18Na	793.2292	0.4	4.3	1	100.00	34.5	even	ok
	1	C42H30F8N6Na	793.2296	1.0	4.3	1	100.00	26.5	even	ok
	1	C30H34F4N10NaO10	793.2288	-0.1	61.2	1	100.00	16.5	even	ok
1156.3628	1	C54H49F3N15O12	1156.3632	0.3	323.0	1	100.00	36.5	even	ok

Figure S96. HRMS spectrum of **9d**.

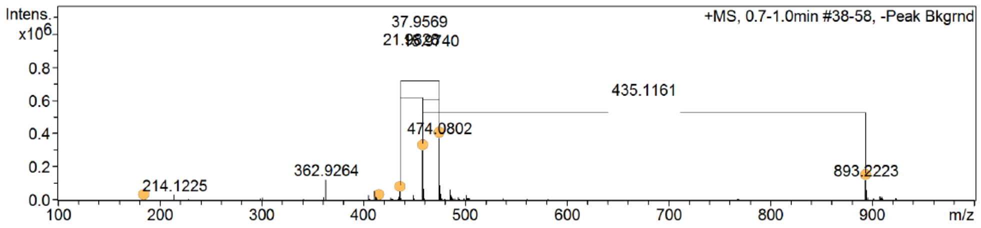

Meas. m/z	#	Ion Formula	m/z	err [ppm]	mSigma	# mSigma	Score	rdb	e⁻ Conf	N-Rule
184.0020	1	C6H3KN5	184.0020	0.1	353.8	1	100.00	7.5	even	ok
	1	C6H3KN5	184.0020	0.1	353.8	1	100.00	7.5	even	ok
415.1950	1	C17H23N10O3	415.1949	-0.3	131.0	1	100.00	11.5	even	ok
	1	C17H23N10O3	415.1949	-0.3	131.0	1	100.00	11.5	even	ok
	1	C9H24F5N10O3	415.1948	-0.6	98.0	1	100.00	0.5	even	ok
	1	C9H24F5N10O3	415.1948	-0.6	98.0	1	100.00	0.5	even	ok
	1	C9H24F5N10O3	415.1948	-0.6	98.0	1	100.00	0.5	even	ok
	1	C9H24F5N10O3	415.1948	-0.6	98.0	1	100.00	0.5	even	ok
	1	C9H24F5N10O3	415.1948	-0.6	98.0	1	100.00	0.5	even	ok
	1	C9H24F5N10O3	415.1948	-0.6	98.0	1	100.00	0.5	even	ok
436.1233	4	C15H22F4NO9	436.1225	-1.9	34.6	4	47.25	3.5	even	ok
	3	C16H18F4N5O5	436.1239	1.2	21.0	3	76.87	8.5	even	ok
	2	C20H13F3N9	436.1241	1.6	11.2	2	85.10	17.5	even	ok
	1	C19H17F3N5O4	436.1227	-1.5	4.1	1	100.00	12.5	even	ok
	3	C11H20F5N5NaO6	436.1226	-1.7	51.9	3	39.48	1.5	even	ok
	7	C6H16F6N11O5	436.1235	0.3	71.2	7	21.06	1.5	even	ok
	6	C12H23F5NO10	436.1237	0.7	53.0	6	36.10	-0.5	even	ok
	5	C10H11F5N15	436.1237	0.7	39.8	5	53.41	10.5	even	ok
	4	C15H22F4NO9	436.1225	-1.9	34.6	4	47.25	3.5	even	ok
	2	C20H13F3N9	436.1241	1.6	11.2	2	85.10	17.5	even	ok

Meas. m/z	#	Ion Formula	m/z	err [ppm]	mSigma	# mSigma	Score	rdb	e⁻ Conf	N-Rule
	1	C9H20F5KN9O3	436.1241	1.7	57.5	1	100.00	1.5	even	ok
	1	C19H17F3N5O4	436.1227	-1.5	4.1	1	100.00	12.5	even	ok
	3	C16H18F4N5O5	436.1239	1.2	21.0	3	76.87	8.5	even	ok
	1	C12H19F4KN9O2	436.1229	-0.9	45.7	1	100.00	5.5	even	ok
	2	C9H20F5KN9O3	436.1241	1.7	57.5	2	59.02	1.5	even	ok
	5	C10H11F5N15	436.1237	0.7	39.8	5	53.41	10.5	even	ok
	7	C6H16F6N11O5	436.1235	0.3	71.2	7	21.06	1.5	even	ok
	1	C9H20F5KN9O3	436.1241	1.7	57.5	1	100.00	1.5	even	ok
	1	C15H15F4N9NaO	436.1228	-1.3	20.2	1	100.00	10.5	even	ok
	1	C9H20F5KN9O3	436.1241	1.7	57.5	1	100.00	1.5	even	ok
	2	C12H16F5N9NaO2	436.1239	1.3	38.7	2	63.16	6.5	even	ok
	1	C9H20F5KN9O3	436.1241	1.7	57.5	1	100.00	1.5	even	ok
	4	C6H18F3N11NaO7	436.1235	0.3	70.4	4	27.96	1.5	even	ok
	1	C9H20F5KN9O3	436.1241	1.7	57.5	1	100.00	1.5	even	ok
	6	C12H23F5NO10	436.1237	0.7	53.0	6	36.10	-0.5	even	ok
458.1062	6	C17H13F4N9NaO	458.1071	2.1	14.2	6	54.23	13.5	even	ok
	7	C16H17F4N5NaO5	458.1058	-0.8	27.5	7	54.16	8.5	even	ok
	8	C17H22FNNaO11	458.1069	1.6	28.9	8	44.55	6.5	even	ok
	9	C30H14FN3Na	458.1064	0.5	56.5	9	26.11	24.5	even	ok
	1	C19H19F6KN3O	458.1064	0.4	36.9	1	93.65	8.5	even	ok
	2	C17H16F3KN9O	458.1061	-0.1	37.0	2	100.00	12.5	even	ok
	3	C22H18F5KN3	458.1052	-2.1	38.1	3	62.92	12.5	even	ok
	4	C17H25KNO11	458.1059	-0.6	49.1	4	64.33	5.5	even	ok
	5	C27H18FKN3O	458.1065	0.8	56.4	5	49.23	19.5	even	ok
	6	C30H17KN3	458.1054	-1.7	69.7	6	25.12	23.5	even	ok
	1	C19H19F6KN3O	458.1064	0.4	36.9	1	100.00	8.5	even	ok
	1	C17H16F3KN9O	458.1061	-0.1	37.0	1	100.00	12.5	even	ok
	1	C27H18FKN3O	458.1065	0.8	56.4	1	100.00	19.5	even	ok
	1	C30H17KN3	458.1054	-1.7	69.7	1	100.00	23.5	even	ok
	1	C30H17KN3	458.1054	-1.7	69.7	1	100.00	23.5	even	ok
	1	C30H17KN3	458.1054	-1.7	69.7	1	100.00	23.5	even	ok
	1	C18H16N7O8	458.1055	-1.5	12.3	4	86.39	14.5	even	ok
	1	C18H16N7O8	458.1055	-1.5	12.3	1	100.00	14.5	even	ok
	1	C18H9N15Na	458.1058	-0.9	4.5	1	100.00	21.5	even	ok
	1	C17H25KNO11	458.1059	-0.6	49.1	1	100.00	5.5	even	ok
	1	C20H21NNaO10	458.1058	-0.9	13.4	1	100.00	10.5	even	ok
	1	C20H12F3N9Na	458.1060	-0.4	4.5	1	100.00	17.5	even	ok
	1	C16H17F4N5NaO5	458.1058	-0.8	27.5	1	100.00	8.5	even	ok
	5	C24H14F2N5O3	458.1059	-0.6	23.5	5	84.33	19.5	even	ok
	1	C20H12F3N9Na	458.1060	-0.4	4.5	1	100.00	17.5	even	ok
	1	C16H17F4N5NaO5	458.1058	-0.8	27.5	1	100.00	8.5	even	ok
	1	C10H10F5N15Na	458.1056	-1.3	46.4	1	100.00	10.5	even	ok
	1	C12H22F5NNaO10	458.1056	-1.3	59.5	1	100.00	-0.5	even	ok
	1	C6H15F6N11NaO5	458.1054	-1.7	77.8	1	100.00	1.5	even	ok
	1	C21H15F3N5O4	458.1071	1.9	6.3	1	88.12	15.5	even	ok
	2	C19H12N11O4	458.1068	1.4	6.3	2	100.00	19.5	even	ok
	3	C20H19F3NO8	458.1057	-1.0	12.3	3	97.43	10.5	even	ok
	1	C6H15F6N11NaO5	458.1054	-1.7	77.8	1	100.00	1.5	even	ok
	4	C18H16N7O8	458.1055	-1.5	12.3	4	86.39	14.5	even	ok
	5	C24H14F2N5O3	458.1059	-0.6	23.5	5	84.33	19.5	even	ok
	6	C17H20F4NO9	458.1069	1.5	29.0	6	61.04	6.5	even	ok
	1	C21H15F3N5O4	458.1071	1.9	6.3	1	88.12	15.5	even	ok
	2	C19H12N11O4	458.1068	1.4	6.3	2	100.00	19.5	even	ok
	3	C20H19F3NO8	458.1057	-1.0	12.3	3	97.43	10.5	even	ok
	4	C18H16N7O8	458.1055	-1.5	12.3	4	86.39	14.5	even	ok
	6	C17H20F4NO9	458.1069	1.5	29.0	6	61.04	6.5	even	ok
	1	C18H9N15Na	458.1058	-0.9	4.5	1	84.53	21.5	even	ok
	2	C20H12F3N9Na	458.1060	-0.4	4.5	2	94.16	17.5	even	ok
	3	C22H15F6N3Na	458.1062	0.1	4.6	3	100.00	13.5	even	ok
	4	C21H17N5NaO6	458.1071	2.0	8.7	4	61.31	15.5	even	ok
	5	C20H21NNaO10	458.1058	-0.9	13.4	5	72.13	10.5	even	ok

Figure S97. HRMS spectrum of **9e**.

[1] D. R. Stirling, M. J. Swain-Bowden, A. M. Lucas, A. E. Carpenter, B. A. Cimini, A. Goodman. CellProfiler 4: improvements in speed, utility and usability. *BMC Bioinformatics*, **2021**, *22* (1), 433; https://doi.org/10.1186/s12859-021-04344-9.
